# Supplementary material for: The risk of bleeding and perforation from sigmoidoscopy or colonoscopy in colorectal cancer screening: A systematic review and meta-analyses
Source: PLoS One. 2023 Oct 31;18(10):e0292797. doi: 10.1371/journal.pone.0292797 (PMC10617695; doi:10.1371/journal.pone.0292797)
Supplement: S1 File — Appendix 1 –Deviations from the published protocol in the systematic review, Appendix 2 –Study eligibility, Appendix 3 –Search strategy & information sources, Appendix 4 –Reasons for all studies excluded (total list), Appendix 5 –Data extraction templates, Appendix 6 –The GRADE approach, Appendix 7 –Subcategories of bleeding, Appendix 8 –Subcategories of perforation, Appendix 9 –Study characteristics of special case studies and studies with an unscreened control group, Appendix 10 –Characteristics of additional subpopulations, Appendix 11 –Conversion factor for each procedure group, Appendix 12 –Combination of subcategories. Appendix 13 –Characteristics of procedure groups, Appendix 14 –Adequacy of harm measurement across studies for bleeding, Appendix 15 –Adequacy of harm measurement across studies for Perforation, Appendix 16 –Bias distributions across all studies that assess bleeding, Appendix 17 –Bias distributions across all studies that assess perforation, Appendix 18 –Characteristics of the external validity for bleeding, Appendix 19 –Characteristics of the external validity for perforation, Appendix 20 –The consequences of bleeding, Appendix 21 –The consequences of perforation, Appendix 22 –Factors potentially modifying occurrences of bleeding. Appendix 23 –Factors potentially modifying occurrences of perforation, Appendix 24 –Bleeding and perforation assessed in six former systematic reviews, Appendix 25 –Comparison between former systematic reviews that assess bleeding and current review, Appendix 26 –Comparison between former systematic reviews that assess perforation and current review. (PDF) [file pone.0292797.s002.pdf]

S1 – Appendices

Table of contents

1 APPENDIX 1 - DEVIATIONS FROM THE PUBLISHED PROTOCOL IN THE SYSTEMATIC REVIEW 3

2 APPENDIX 2 – STUDY ELIGIBILITY 4

3 APPENDIX 3 – SEARCH STRATEGY & INFORMATION SOURCES 5

SEARCH STRATEGIES (PUBMED, EMBASE, CINAHL, PSYCINFO AND THE COCHRANE DATABASE) 5
PUBMED MESH 5
PUBMED KEYWORD 7
EMBASE EMTREE 7
EMBASE KEYWORD 11
CINAHL 19
PSYCINFO 23
THE COCHRANE DATABASE 27

4 APPENDIX 4 - REASONS FOR ALL STUDIES EXCLUDED (TOTAL LIST) 27

5 APPENDIX 5 - DATA EXTRACTION TEMPLATES 58

TEMPLATE 1 – STUDY CHARACTERISTICS 58
TEMPLATE 2 – OUTCOME DATA 60

6 APPENDIX 6 – THE GRADE APPROACH 61

7 APPENDIX 7 – SUBCATEGORIES OF BLEEDING 65

8 APPENDIX 8 – SUBCATEGORIES OF PERFORATION 69

9 APPENDIX 9 – STUDY CHARACTERISTICS OF SPECIAL CASE STUDIES AND STUDIES WITH AN UNSCREENED CONTROL GROUP 71

10 APPENDIX 10 – CHARACTERISTICS OF ADDITIONAL SUBPOPULATIONS 73

11 APPENDIX 11 – CONVERSION FACTOR FOR EACH PROCEDURE GROUP 73

12 APPENDIX 12 – COMBINATION OF SUBCATEGORIES 74

13 APPENDIX 13 – CHARACTERISTICS OF PROCEDURE GROUPS 75

14 APPENDIX 14 - ADEQUACY OF HARM MEASUREMENT ACROSS STUDIES FOR BLEEDING 76

15 APPENDIX 15 - ADEQUACY OF HARM MEASUREMENT ACROSS STUDIES FOR PERFORATION 76

16 APPENDIX 16 - BIAS DISTRIBUTIONS ACROSS ALL STUDIES THAT ASSESS BLEEDING 77

17 APPENDIX 17 - BIAS DISTRIBUTIONS ACROSS ALL STUDIES THAT ASSESS PERFORATION 79

18 APPENDIX 18 – CHARACTERISTICS OF THE EXTERNAL VALIDITY FOR BLEEDING 80

19 APPENDIX 19 – CHARACTERISTICS OF THE EXTERNAL VALIDITY FOR PERFORATION 91

20 APPENDIX 20 – THE CONSEQUENCES OF BLEEDING 99

21 APPENDIX 21 – THE CONSEQUENCES OF PERFORATION 99

22 APPENDIX 22 - FACTORS POTENTIALLY MODIFYING OCCURRENCES OF BLEEDING 100

23 APPENDIX 23 - FACTORS POTENTIALLY MODIFYING OCCURRENCES OF PERFORATION 100

24 APPENDIX 24 – BLEEDING AND PERFORATION ASSESSED IN SIX FORMER SYSTEMATIC REVIEWS **101**

25 APPENDIX 25 – COMPARISON BETWEEN FORMER SYSTEMATIC REVIEWS THAT ASSESS BLEEDING AND CURRENT REVIEW. **101**

___ = *NO HARM ESTIMATES IN THE RESPECTIVE PROCEDURE* 102

26 APPENDIX 26 – COMPARISON BETWEEN FORMER SYSTEMATIC REVIEWS THAT ASSESS PERFORATION AND CURRENT REVIEW. **102**

REFERENCE LIST: **103**

1 Appendix 1 - Deviations from the published protocol in the systematic review

This appendix provides an account of the deviations from the registered protocol in the conduct of the systematic review with reasons for these. The protocol for the review was initially registered on PROSPERO, September 22nd, 2017, and subsequently amended with minor clarifications of the wording on November 3rd, 2017. During the review process, we encountered circumstances that necessitated adjustments to the original plan for the review.

The first deviation pertains to the planned supplementation of our database search with a targeted grey literature search in Google Scholar. Due to the extensive volume of evidence yielded by our systematic search, encompassing 134 studies eligible for review, we decided against conducting the intended targeted grey literature search. This alteration in the protocol was made in consideration of the comprehensive evidence obtained through our systematic search.

The second deviation concerns the scope of our review, which specifically focus on the diagnostic step of the screening cascade. Consequently, we excluded the assessment of potential harm occurring prior to the diagnostic screening procedures, e.g., adverse events associated with bowel preparation, and adverse events following screening procedures, such as further diagnostic work-up or surgery. This approach aligns with the methodology employed in previous systematic reviews conducted in this field.

In addition to the aforementioned deviations, we decided to present the review findings in separate publications. The reasons for this was that we discovered much more evidence than expected. Moreover, we encountered an unexpected assortment of physical harms associated with colorectal cancer screening procedures (CRCSPs). The studies included for review had considerable heterogeneity and frequently suffered from inadequate quality in terms of definitions, assessments, and reporting of harms. Surprisingly, previous systematic reviews had paid limited attention to these issues, in our view. Consequently, we concluded that the most appropriate course of action was to report the findings of the review in a series of separate publications, which would allow adequate detail and attention to the findings. Thus, this particular publication exclusively focuses on reporting the review findings pertaining to studies

that assessed the two most frequently reported, and most severe, procedure-related physical harms associated with CRCSPs: bleeding and perforation. Findings on other aspects identified in the review are reported in other pending publications (1).

2 Appendix 2 – Study eligibility

Appendix 2: In- and exclusion criteria.

	**Inclusion**	**Exclusion**
**Population**	We include all studies whose study population is aged 40-80 years.<br><br>Average risk of colorectal cancer and is asymptomatic regarding signs of colorectal cancer.<br><br>Studies with mixed populations in cases where data and analysis are stratified, so that it is possible to separate relevant and irrelevant data regarding our target group.	Studies that include people at higher risk of colorectal cancer than average, such as people with a genetic predisposition, people recruited based on personal or family history to colorectal cancer,<br><br>Non-screening population: people with a current or previous colorectal cancer diagnosis or people with other diseases that may increase the risk of developing colorectal cancer, such as inflammatory bowel disease.
**Setting**	Settings comparable to clinical screening settings in real life.	Screening settings that diverge from normal screening settings, so comparison between the settings is not possible. This includes general practice or specialized endoscopic treatment centers.
**Intervention**	Colorectal screening techniques whether it is a first line or second line procedure: faecal occult blood test (guaiac or immunochemical tests), sigmoidoscopy and colonoscopy, and any type of combination.	Colorectal screening techniques: stool testing such as in-office digital rectal exam (DRE). Genetic testing for increased risk of colorectal cancer. In addition, blood tests (septins), stool DNA test and capsule endoscopy or CT colonography.<br><br>Any interventions after the screening – i.e., people referred for further work-up/treatment or people participating in surveillance programs due to former identification of cancer or cancer precursors, i.e., polyps.<br><br>Alternative treatment meant to reduce harm, e.g., pain medication, music therapy etc.

**Outcomes**	Physical harm related to colorectal cancer screening resulting from sigmoidoscopy and/or colonoscopy with or without polypectomy	Studies not reporting about physical harms in title, abstract or full text.<br><br>Reporting expected harms related to colorectal cancer screening or reporting harms based on the opinion of physicians or other health professionals. Including simulated statistics of harm in microsimulation studies.
**Study design**	Studies of any type which reports original research: RCTs, CCTs, cohort studies, case-control studies, cross-sectional studies, case series and case reports.	Studies which are not reporting original data/research: journalism, editorials, narrative reviews, and opinions such as letters or comments.<br><br>Studies that report harms and compare it in relation to screening methods or techniques, such as types of colonoscope, different anaesthetic procedures, different bowel preparation schemes or different polypectomy techniques.<br><br>Systematics reviews.<br>Qualitative studies.<br>Ongoing studies.

3 Appendix 3 – Search strategy & information sources

Search strategies (Pubmed, Embase, Cinahl, PsycInfo and the Cochrane database)
CRC screening AND physical harm

CRC screening = ((Screening AND laxative) OR (Screening AND CRC) OR CRC screening technologies)
Search strategies were performed via use of index words in databases supplemented by searches via selected keywords in separate searches. The original search was developed for Medline/Pubmed and following translated to the terminology used in other databases. The search stratgeies are presented below for Medline, Pubmed and Embase. The Cochrane library use the same terminology as in Medline and is therefore not described separately below.

Pubmed MeSH
((("Colonoscopy"[Mesh] OR "Sigmoidoscopy"[Mesh] OR "Occult Blood"[Mesh] OR "Colonic Neoplasms/surgery"[Mesh] OR "Intestinal Polyps/surgery"[Mesh] OR "Colorectal

Neoplasms/prevention and control"[Mesh])) OR (((("intestinal polyps"[MeSH Terms] OR "cecal neoplasms"[MeSH Terms] OR "colorectal neoplasms"[MeSH Terms]) OR ("adenoma"[MeSH Terms] AND ("Intestine, Large"[Mesh] OR "Intestinal Neoplasms"[Mesh])))) AND ("Early Diagnosis"[Mesh] OR "Secondary Prevention"[Mesh] OR "Early Detection of Cancer"[Mesh] OR "Mass Screening"[Mesh] OR "Preventive Health Services"[Mesh] OR "Early Medical Intervention"[Mesh]))) OR ((((("Colonoscopy"[Mesh] OR "Sigmoidoscopy"[Mesh] OR "Occult Blood"[Mesh] OR "Colonic Neoplasms/surgery"[Mesh] OR "Intestinal Polyps/surgery"[Mesh] OR "Colorectal Neoplasms/prevention and control"[Mesh])) OR ("Early Diagnosis"[Mesh] OR "Secondary Prevention"[Mesh] OR "Early Detection of Cancer"[Mesh] OR "Mass Screening"[Mesh] OR "Preventive Health Services"[Mesh] OR "Early Medical Intervention"[Mesh]))) AND ("Cathartics"[Mesh] OR "Laxatives"[Mesh]))

AND

"Postoperative Complications"[Mesh] OR "Intraoperative Complications"[Mesh] OR "Death"[Mesh] OR "Pain"[Mesh] OR "Pain Measurement"[Mesh] OR "Signs and Symptoms, Digestive"[Mesh] OR "Infection"[Mesh] OR "Blood Loss, Surgical"[Mesh] OR "Heart Diseases"[Mesh] OR "Hypovolemia"[Mesh] OR "Ischemia"[Mesh] OR "Hypersensitivity"[Mesh] OR "Inflammation"[Mesh] OR "Shock"[Mesh] OR "Ulcer"[Mesh] OR "Intestinal Perforation"[Mesh] OR "Abdominal Injuries"[Mesh] OR "Iatrogenic Disease"[Mesh] OR "Medical Errors"[Mesh] OR "Safety"[Mesh] OR "Long Term Adverse Effects"[Mesh] OR "Water-Electrolyte Imbalance"[Mesh] OR "Colon/injuries"[Mesh] OR "Early Diagnosis/adverse effects"[Mesh] OR "Early Diagnosis/mortality"[Mesh] OR "Early Detection of Cancer/adverse effects"[Mesh] OR "Early Detection of Cancer/mortality"[Mesh] OR "Mass Screening/adverse effects"[Mesh] OR "Mass Screening/mortality"[Mesh] OR "Preventive Health Services/adverse effects"[Mesh] OR "Preventive Health Services/complications"[Mesh] OR "Preventive Health Services/mortality"[Mesh] OR "Colonoscopy/adverse effects"[Mesh] OR "Colonoscopy/complications"[Mesh] OR "Colonoscopy/mortality"[Mesh] OR "Sigmoidoscopy/adverse effects"[Mesh] OR "Sigmoidoscopy/complications"[Mesh] OR "Sigmoidoscopy/mortality"[Mesh] OR "Occult Blood/adverse effects"[Mesh] OR "Cathartics/adverse effects"[Mesh] OR "Cathartics/poisoning"[Mesh] OR "Cathartics/toxicity"[Mesh] OR "Laxatives/adverse effects"[Mesh] OR "Laxatives/poisoning"[Mesh] OR "Laxatives/toxicity"[Mesh]

Pubmed keyword

(((((((fecal immunochemical test*[tiab] OR fobt[tiab] OR colonoscop*[tiab] OR sigmoidoscop*[tiab] OR occult blood[tiab] OR hemoccult*[tiab] OR polypect*[tiab])) OR (early detect*[tiab] OR early diagnos*[tiab] OR prevent*[tiab] OR screen*[tiab] OR early intervent*[tiab]))) AND (laxativ*[tiab] OR Cathartic*[tiab] OR purgativ*[tiab] OR bowel prepa*[tiab] OR colon cleans*[tiab]))) OR (((colorectal neoplasm*[tiab] OR colorectal adenoma*[tiab] OR colorectal carcinoma*[tiab] OR colorectal carcinogenesis[tiab] OR colorectal adenocarcinoma*[tiab] OR colorectal tumor*[tiab] OR colorectal polyp*[tiab] OR colorectal cancer*[tiab] OR colon adenoma*[tiab] OR colon carcinoma*[tiab] OR colon carcinogenesis[tiab] OR colon adenocarcinoma*[tiab] OR colon tumor*[tiab] OR colon polyp*[tiab] OR colon cancer*[tiab] OR colon neoplasm*[tiab] OR bowel cancer*[tiab] OR intestine cancer*[tiab] OR intestinal adenoma*[tiab] OR cecal neoplasm*[tiab] OR intestinal polyp*[tiab])) AND (early detect*[tiab] OR early diagnos*[tiab] OR prevent*[tiab] OR screen*[tiab] OR early intervent*[tiab]))) OR (fecal immunochemical test*[tiab] OR fobt[tiab] OR colonoscop*[tiab] OR sigmoidoscop*[tiab] OR occult blood[tiab] OR hemoccult*[tiab] OR polypect*[tiab])

AND

"intraoperative complication*"[tiab] OR "adverse outcome*"[tiab] OR "adverse event*"[tiab] OR "postoperative complication*"[tiab] OR post procedure infection*[tiab] OR "symptomatic complaint*"[tiab] OR "physical complaint*"[tiab] OR discomfort*[tiab] OR transmural burn syndrome[tiab] OR Postpolypectomy syndrome[tiab] OR "gastrointestinal event*"[tiab] OR "cardiopulmonary event*"[tiab] OR gas explosion[tiab] OR intracolonic explosion[tiab] OR perforation[tiab] OR perforations[tiab] OR "colon injury"[tiab] OR "physical symptom*"[tiab] OR "physical discomfort*"[tiab] OR "physical harm*"[tiab] OR "colonoscopy burden" OR "intraoperative bleeding"[tiab] OR "postoperative bleeding"[tiab] OR "intraoperative haemorrhage"[tiab] OR "postoperative haemorrhage"[tiab]

Embase Emtree

1. early diagnosis.sh.
2. early cancer diagnosis.sh.
3. cancer prevention.sh.
4. secondary prevention.sh.
5. prophylactic surgical procedure.sh.
6. primary prevention.sh.

7. screening test.sh.
8. early intervention.sh.
9. mass screening.sh.
10. screening.sh.
11. rescreening.sh.
12. cancer screening.sh.
13. 1 or 2 or 3 or 4 or 5 or 6 or 7 or 8 or 9 or 10 or 11 or 12
14. (colonoscopy or colonoscope or flexible colonoscope).sh.
15. (sigmoidoscope or flexible sigmoidoscope or rigid sigmoidoscope).sh.
16. (sigmoidoscopy or occult blood).sh.
17. exp occult blood test/
18. exp polypectomy/
19. exp endoscopic biopsy/
20. exp endoscopic mucosal resection/
21. exp endoscopic polypectomy/
22. gastrointestinal endoscopy/
23. gastrointestinal biopsy/
24. rectum biopsy/
25. intestine biopsy/
26. colon biopsy/
27. 14 or 15 or 16 or 17 or 18 or 19 or 20 or 21 or 22 or 23 or 24 or 25 or 26
28. 13 and 27
29. cecum tumor/
30. cecum cancer/
31. cecum carcinoma/
32. colon tumor/
33. colon cancer/
34. colon carcinoma/
35. colon adenoma/
36. colon polyp/
37. colon adenocarcinoma/
38. colorectal tumor/

39. colorectal cancer/
40. colorectal carcinoma/
41. colorectal polyp/
42. colorectal adenoma/
43. intestine tumor/
44. intestine cancer/
45. intestine carcinoma/
46. intestine polyp/
47. rectum tumor/
48. rectum cancer/
49. rectum carcinoma/
50. rectum polyp/
51. rectum adenoma/
52. large intestine tumor/
53. large intestine cancer/
54. sigmoid carcinoma/
55. gastrointestinal tumor/
56. gastrointestinal carcinoma/
57. digestive system tumor/
58. digestive system cancer/
59. 29 or 30 or 31 or 32 or 33 or 34 or 35 or 36 or 37 or 38 or 39 or 40 or 41 or 42 or 43 or 44 or 45 or 46 or 47 or 48 or 49 or 50 or 51 or 52 or 53 or 54 or 55 or 56 or 57 or 58
60. 13 and 59
61. laxative/
62. exp laxative/
63. exp intestine contraction stimulating agent/
64. intestine preparation/
65. 61 or 62 or 63 or 64
66. 13 and 65
67. 28 or 60 or 66
68. colonoscope/am [Adverse Device Effect]

69. gastrointestinal endoscopy/ae [Adverse Drug Reaction]
70. sigmoidoscope/am [Adverse Device Effect]
71. sigmoidoscopy/ae [Adverse Drug Reaction]
72. early diagnosis/ae [Adverse Drug Reaction]
73. prophylaxis/co [Complication]
74. mass screening/ae [Adverse Drug Reaction]
75. endoscopic surgery/ae [Adverse Drug Reaction]
76. anesthesia complication/
77. lung complication/
78. disease exacerbation/
79. infection complication/
80. infectious complication/
81. neurological complication/
82. hemoperitoneum/
83. mucosal bleeding/
84. operative blood loss/
85. postoperative hemorrhage/
86. peroperative complication/
87. postoperative complication/
88. postoperative cognitive dysfunction/
89. postoperative delirium/
90. postoperative ileus/
91. postoperative infection/
92. exp "postoperative nausea and vomiting"/
93. postoperative pain/
94. postoperative thrombosis/
95. iatrogenic disease/
96. colon perforation/
97. intestine perforation/
98. rectum perforation/
99. large intestine perforation/
100. digestive system perforation/

101. medical error/

102. surgical error/

103. therapeutic error/

104. abdominal discomfort/

105. abdominal cramp/

106. bloating/

107. epigastric discomfort/

108. abdominal bleeding/

109. patient harm/

110. patient risk/

111. harm reduction/

112. operative blood loss/

113. exp abdominal pain/

114. 68 or 69 or 70 or 71 or 72 or 73 or 74 or 75 or 76 or 77 or 78 or 79 or 80 or 81 or 82 or 83 or 84 or 85 or 86 or 87 or 88 or 89 or 90 or 91 or 92 or 93 or 94 or 95 or 96 or 97 or 98 or 99 or 100 or 101 or 102 or 103 or 104 or 105 or 106 or 107 or 108 or 109 or 110 or 111 or 112 or 113

115. laxative/ae, to [Adverse Drug Reaction, Drug Toxicity]

116. intestine contraction stimulating agent/ae [Adverse Drug Reaction]

117. 114 or 115 or 116

118. 67 and 117

119. limit 118 to yr="2017 -Current"

Embase keyword

1. early detection of cancer.tw.

2. screening.tw.

3. population screening.tw.

4. health screening.tw.

5. early diagnosis.tw.

6. early cancer diagnosis.tw.

7. early detection of cancer.tw.

8. cancer prevention*.tw.

9. secondary prevention.tw.

10. preventive therap*.tw.

11. preventive treatment*.tw.

12. prophylactic treatment*.tw.

13. prophylactic therap*.tw.

14. prophylactic surgical procedure*.tw.

15. primary prevention.tw.

16. cancer screening.tw.

17. screening test*.tw.

18. Early Medical Intervention*.tw.

19. mass screening.tw.

20. rescreening.tw.

21. 1 or 2 or 3 or 4 or 5 or 6 or 7 or 8 or 9 or 10 or 11 or 12 or 13 or 14 or 15 or 16 or 17 or 18 or 19 or 20

22. purgativ*.tw.

23. laxativ*.tw.

24. cathartic*.tw.

25. bowel prepar*.tw.

26. intestine prepar*.tw.

27. 22 or 23 or 24 or 25 or 26

28. colonoscop*.tw.

29. sigmoidoscop*.tw.

30. occult blood.tw.

31. bowel biopsy.tw.

32. colonic biopsy.tw.

33. intestinal biopsy.tw.

34. sigmoid biopsy.tw.

35. occult blood test.tw.

36. polypectomy.tw.

37. endoscopic biopsy.tw.

38. endoscopic mucosal resection.tw.

39. endoscopic polypectomy.tw.

40. gastrointestinal biopsy.tw.

41. rectum biopsy.tw.
42. intestine biopsy.tw.
43. colon biopsy.tw.
44. 28 or 29 or 30 or 31 or 32 or 33 or 34 or 35 or 36 or 37 or 38 or 39 or 40 or 41 or 42 or 43
45. 21 and 27
46. bowel tumor.tw.
47. bowel tumour.tw.
48. bowel cancer.tw.
49. bowel carcinoma.tw.
50. bowel polyp.tw.
51. bowel adenoma.tw.
52. bowel adenocarcinoma.tw.
53. bowel neoplasm.tw.
54. cecum tumour.tw.
55. cecum polyp.tw.
56. cecum adenoma.tw.
57. cecum adenocarcinoma.tw.
58. cecum neoplasm.tw.
59. caecal tumor.tw.
60. caecal tumour.tw.
61. caecal cancer.tw.
62. caecal carcinoma.tw.
63. caecal polyp.tw.
64. caecal adenoma.tw.
65. caecal adenocarcinoma.tw.
66. ceacal neoplasm.tw.
67. cecal tumor.tw.
68. cecal tumour.tw.
69. cecal cancer.tw.
70. cecal carcinoma.tw.
71. cecal polyp.tw.

72. cecal adenoma.tw.
73. cecal adenocarcinoma.tw.
74. cecal neoplasm.tw.
75. coecum tumor.tw.
76. coecum tumour.tw.
77. coecum cancer.tw.
78. coecum carcinoma.tw.
79. coecum polyp.tw.
80. coecum adenoma.tw.
81. coecum adenocarcinoma.tw.
82. coecum neoplasm.tw.
83. colon tumour.tw.
84. colon neoplasm.tw.
85. colonic tumor.tw.
86. colonic tumour.tw.
87. colonic cancer.tw.
88. colonic carcinoma.tw.
89. colonic polyp.tw.
90. colonic adenoma.tw.
91. colonic adenocarcinoma.tw.
92. colonic neoplasm.tw.
93. colorectal tumour.tw.
94. colorectal adenocarcinoma.tw.
95. colorectal neoplasm.tw.
96. intestine tumour.tw.
97. intestine adenoma.tw.
98. intestine neoplasm.tw.
99. intestinal tumor.tw.
100. intestinal tumour.tw.
101. intestinal cancer.tw.
102. intestinal carcinoma.tw.
103. intestinal polyp.tw.

104. intestinal adenoma.tw.
105. intestinal adenocarcinoma.tw.
106. intestinal neoplasm.tw.
107. rectum tumour.tw.
108. rectum adenocarcinoma.tw.
109. rectum neoplasm.tw.
110. rectal tumor.tw.
111. rectal tumour.tw.
112. rectal cancer.tw.
113. rectal carcinoma.tw.
114. rectal polyp.tw.
115. rectal adenoma.tw.
116. rectal adenocarcinoma.tw.
117. rectal neoplasm.tw.
118. large intestine tumour.tw.
119. large intestine carcinoma.tw.
120. large intestine polyp.tw.
121. large intestine adenoma.tw.
122. large intestine adenocarcinoma.tw.
123. large intestine neoplasm.tw.
124. large bowel tumor.tw.
125. large bowel tumour.tw.
126. large bowel cancer.tw.
127. large bowel carcinoma.tw.
128. large bowel polyp.tw.
129. large bowel adenoma.tw.
130. large bowel adenocarcinoma.tw.
131. large bowel neoplasm.tw.
132. sigmoid tumor.tw.
133. sigmoid tumour.tw.
134. sigmoid cancer.tw.
135. sigmoid polyp.tw.

136. sigmoid adenoma.tw.
137. sigmoid adenocarcinoma.tw.
138. sigmoid neoplasm.tw.
139. gastrointestinal tumour.tw.
140. gastrointestinal cancer.tw.
141. gastrointestinal polyp.tw.
142. gastrointestinal adenoma.tw.
143. gastrointestinal adenocarcinoma.tw.
144. gastrointestinal neoplasm.tw.
145. digestive system tumour.tw.
146. digestive system carcinoma.tw.
147. digestive system polyp.tw.
148. digestive system adenoma.tw.
149. digestive system adenocarcinoma.tw.
150. digestive system neoplasm.tw.
151. cecum tumor*.tw.
152. cecum cancer*.tw.
153. cecum carcinoma*.tw.
154. colon tumor*.tw.
155. colon cancer*.tw.
156. colon carcinoma*.tw.
157. colon adenoma*.tw.
158. colon polyp*.tw.
159. colon adenocarcinoma*.tw.
160. colorectal tumor*.tw.
161. colorectal cancer*.tw.
162. colorectal carcinoma*.tw.
163. colorectal polyp*.tw.
164. colorectal adenoma*.tw.
165. intestine tumor*.tw.
166. intestine cancer*.tw.
167. intestine carcinoma*.tw.

168. intestine polyp*.tw.
169. rectum tumor*.tw.
170. rectum cancer*.tw.
171. rectum carcinoma*.tw.
172. rectum polyp*.tw.
173. rectum adenoma*.tw.
174. large intestine tumor*.tw.
175. large intestine cancer*.tw.
176. sigmoid carcinoma*.tw.
177. gastrointestinal tumor*.tw.
178. gastrointestinal carcinoma*.tw.
179. digestive system tumor*.tw.
180. digestive system cancer*.tw.
181. 46 or 47 or 48 or 49 or 50 or 51 or 52 or 53 or 54 or 55 or 56 or 57 or 58 or 59 or 60 or 61 or 62 or 63 or 64 or 65 or 66 or 67 or 68 or 69 or 70 or 71 or 72 or 73 or 74 or 75 or 76 or 77 or 78 or 79 or 80 or 81 or 82 or 83 or 84 or 85 or 86 or 87 or 88 or 89 or 90 or 91 or 92 or 93 or 94 or 95 or 96 or 97 or 98 or 99 or 100 or 101 or 102 or 103 or 104 or 105 or 106 or 107 or 108 or 109 or 110 or 111 or 112 or 113 or 114 or 115 or 116 or 117 or 118 or 119 or 120 or 121 or 122 or 123 or 124 or 125 or 126 or 127 or 128 or 129 or 130 or 131 or 132 or 133 or 134 or 135 or 136 or 137 or 138 or 139 or 140 or 141 or 142 or 143 or 144 or 145 or 146 or 147 or 148 or 149 or 150 or 151 or 152 or 153 or 154 or 155 or 156 or 157 or 158 or 159 or 160 or 161 or 162 or 163 or 164 or 165 or 166 or 167 or 168 or 169 or 170 or 171 or 172 or 173 or 174 or 175 or 176 or 177 or 178 or 179 or 180
182. 21 and 181
183. 44 or 45 or 182
184. intraoperative complication*.tw.
185. (postoperative nausea and vomiting).tw.
186. bowel perforation.tw.
187. cecum perforation.tw.
188. colonic perforation.tw.
189. intestinal perforation.tw.

190. sigmoid perforation.tw.
191. gastrointestinal perforation.tw.
192. extracolonic finding*.tw.
193. Postpolypectomy syndrome.tw.
194. transmural burn syndrome.tw.
195. Harms of colonoscopy.tw.
196. Harms of sigmoidoscopy.tw.
197. Harms of bowel preparation.tw.
198. physical harm*.tw.
199. procedure related harm*.tw.
200. "anesthesia complication".tw.
201. lung complication*.tw.
202. infectious complication*.tw.
203. neurological complication*.tw.
204. hemoperitoneum.tw.
205. mucosal bleeding.tw.
206. operative blood loss.tw.
207. postoperative hemorrhage.tw.
208. peroperative complication*.tw.
209. postoperative complication*.tw.
210. postoperative cognitive dysfunction.tw.
211. postoperative delirium.tw.
212. postoperative ileus.tw.
213. postoperative infection*.tw.
214. postoperative pain.tw.
215. postoperative thrombosis.tw.
216. iatrogenic disease.tw.
217. colon perforation.tw.
218. intestine perforation.tw.
219. rectum perforation.tw.
220. large intestine perforation.tw.
221. digestive system perforation.tw.

222. medical error.tw.

223. surgical error.tw.

224. therapeutic error.tw.

225. abdominal cramp.tw.

226. epigastric discomfort.tw.

227. abdominal bleeding.tw.

228. patient harm.tw.

229. patient risk.tw.

230. harm reduction.tw.

231. surgical blood loss.tw.

232. 184 or 185 or 186 or 187 or 188 or 189 or 190 or 191 or 192 or 193 or 194 or 195 or 196 or 197 or 198 or 199 or 200 or 201 or 202 or 203 or 204 or 205 or 206 or 207 or 208 or 209 or 210 or 211 or 212 or 213 or 214 or 215 or 216 or 217 or 218 or 219 or 220 or 221 or 222 or 223 or 224 or 225 or 226 or 227 or 228 or 229 or 230 or 231

233. 183 and 232

234. limit 233 to yr="2017 - 2022"

Cinahl
<u>Index search:</u>

S127 S76 AND S126

S126 S98 AND S125

S125 S99 OR S100 OR S101 OR S102 OR S103 OR S104 OR S105 OR S106 OR S107 OR S108 OR S109 OR S110 OR S111 OR S112 OR S113 OR S114 OR S115 OR S116 OR S117 OR S118 OR S119 OR S120 OR S121 OR S122 OR S123 OR S124

S124 (MH "Fluid-Electrolyte Imbalance")

S123 (MH "Treatment Complications, Delayed") OR (MH "Adverse Health Care Event")

S122 (MH "Safety")

S121 (MH "Health Care Errors") OR (MH "Treatment Errors") OR (MH "Medication Errors")

S120 (MH "Iatrogenic Disease")

S119 (MH "Abdominal Injuries")

S118 (MH "Intestinal Perforation")

S117 (MH "Ulcer")

S116	(MH "Shock") OR (MH "Toxic Shock Syndrome") OR (MH "Shock, Septic") OR (MH "Shock, Hemorrhagic") OR (MH "Shock, Traumatic") OR (MH "Shock, Surgical") OR (MH "Shock, Cardiogenic")
S115	(MH "Inflammation")
S114	(MH "Hypersensitivity")
S113	(MH "Myocardial Ischemia")
S112	(MH "Heart Diseases")
S111	(MH "Gastrointestinal Hemorrhage")
S110	(MH "Blood Loss, Surgical")
S109	(MH "Surgical Wound Infection")
S108	(MH "Infection")
S107	(MH "Signs and Symptoms, Digestive")
S106	(MH "Pain")
S105	(MH "Death, Sudden, Cardiac")
S104	(MH "Death")
S103	(MH "Intraoperative Complications")
S102	(MH "Postoperative Pain")
S101	(MH "Postoperative Hemorrhage")
S100	(MH "Postoperative Complications")
S99	(MH "Treatment Complications, Delayed")
S98	S80 OR S96 OR S97
S97	S86 AND S92
S96	(S93 OR S94) AND (S80 AND S95)
S95	S93 OR S94
S94	laxatives
S93	cathartic
S92	S87 OR S88 OR S89 OR S90 OR S91
S91	intestinal neoplasm
S90	adenoma
S89	colorectal neoplasms
S88	cecal neoplasms
S87	intestinal polyps

S86	S81 OR S82 OR S83 OR S84 OR S85
S85	Preventive Health Services
S84	mass screening or screening
S83	early detection or early diagnosis or early identification
S82	secondary prevention
S81	early diagnosis or early intervention
S80	S77 OR S78 OR S79
S79	Occult Blood
S78	sigmoidoscopy
S77	colonoscopy

Keyword search:

S76	S46 AND S74
S75	S46 AND S74
S74	S47 OR S48 OR S49 OR S50 OR S51 OR S52 OR S53 OR S54 OR S55 OR S56 OR S57 OR S58 OR S59 OR S60 OR S61 OR S62 OR S63 OR S64 OR S65 OR S66 OR S67 OR S68 OR S69 OR S70 OR S71 OR S72 OR S73
S73	postoperative haemorrhage
S72	intraoperative haemorrhage
S71	postoperative bleeding
S70	intraoperative bleeding
S69	colonoscopy burden
S68	physical harm*
S67	physical discomfort*
S66	physical symptom*
S65	colon injury
S64	perforations
S63	perforation
S62	intracolonic explosion
S61	intracolonic explosion
S60	gas explosion
S59	cardiopulmonary event*

S58	gastrointestinal event*
S57	Postpolypectomy syndrome
S56	transmural burn syndrome
S55	transmural burn syndrome
S54	discomfort*
S53	physical complaint*
S52	symptomatic complaint*
S51	post procedure infection*
S50	postoperative complication*
S49	adverse event*
S48	adverse outcome*
S47	intraoperative complication*
S46	S8 OR S21 OR S45
S45	S43 AND S44
S44	S8 OR S14
S43	S22 OR S23 OR S24 OR S25 OR S26 OR S27 OR S28 OR S29 OR S30 OR S31 OR S32 OR S33 OR S34 OR S35 OR S36 OR S37 OR S38 OR S39 OR S40 OR S41 OR S42
S42	intestinal polyp*
S41	cecal neoplasm*
S40	intestinal adenoma*
S39	intestine cancer*
S38	bowel cancer*
S37	colon neoplasm*
S36	colon cancer*
S35	colon polyp*
S34	colon tumor*
S33	colon adenocarcinoma*
S32	colon carcinogenesis
S31	colon carcinoma*
S30	colon adenoma*
S29	colorectal cancer*

S28	colorectal polyp*
S27	colorectal tumor*
S26	colorectal adenocarcinoma*
S25	colorectal carcinogenesis
S24	colorectal carcinoma*
S23	colorectal adenoma*
S22	colorectal neoplasm*
S21	S14 AND S20
S20	S15 OR S16 OR S17 OR S18 OR S19
S19	colon cleans*
S18	bowel prepa*
S17	purgativ*
S16	Cathartic*
S15	laxativ*
S14	S9 OR S10 OR S11 OR S12 OR S13
S13	early intervent*
S12	screen*
S11	prevent*
S10	early diagnos*
S9	early detect*
S8	S1 OR S2 OR S3 OR S4 OR S5 OR S6 OR S7
S7	polypect*
S6	hemoccult*
S5	occult blood
S4	sigmoidoscop*
S3	colonoscop*
S2	fobt
S1	fecal immunochemical test*

PsycInfo
<u>Index search:</u>

S110S97 AND S108Limiters - Publication Year: 2017-2022

S109S97 AND S108

S108S98 OR S99 OR S100 OR S101 OR S102 OR S103 OR S104 OR S105 OR S106 OR S107

S107DE "Patient Safety"

S106DE "Shock"

S105DE "Inflammation"

S104DE "Ischemia"

S103DE "Infectious Disorders"

S102DE "Digestive System Disorders"

S101DE "Heart Disorders"

S100DE "Death and Dying"

S99DE "Pain"

S98DE "Postsurgical Complications"

S97S79 OR S95 OR S96

S96S85 AND S91

S95(S92 OR S93) AND (S79 AND S94)

S94S92 OR S93

S93laxatives

S92cathartic

S91S86 OR S87 OR S88 OR S89 OR S90

S90intestinal neoplasm

S89adenoma

S88colorectal neoplasms

S87cecal neoplasms

S86intestinal polyps

S85S80 OR S81 OR S82 OR S83 OR S84

S84Preventive Health Services

S83mass screening or screening

S82early detection or early diagnosis or early identification

S81secondary prevention

S80early diagnosis or early intervention

S79S76 OR S77 OR S78

S78Occult Blood

S77sigmoidoscopy

S76colonoscopy

Keyword search:

S75 S46 AND S74 Limiters - Published Date: 20170401-20220131

S74 S47 OR S48 OR S49 OR S50 OR S51 OR S52 OR S53 OR S54 OR S55 OR S56 OR S57 OR S58 OR S59 OR S60 OR S61 OR S62 OR S63 OR S64 OR S65 OR S66 OR S67 OR S68 OR S69 OR S70 OR S71 OR S72 OR S73

S73postoperative haemorrhage

S72intraoperative haemorrhage

S71postoperative bleeding

S70intraoperative bleeding

S69colonoscopy burden

S68physical harm*

S67physical discomfort*

S66physical symptom*

S65colon injury

S64perforations

S63perforation

S62intracolonic explosion

S61intracolonic explosion

S60gas explosion

S59cardiopulmonary event*

S58gastrointestinal event*

S57Postpolypectomy syndrome

S56transmural burn syndrome

S55transmural burn syndrome

S54discomfort*

S53physical complaint*

S52symptomatic complaint*

S51post procedure infection*

S50postoperative complication*

S49adverse event*

S48adverse outcome*

S47intraoperative complication*

S46 S8 OR S21 OR S45

S45 S43 AND S44

S44 S8 OR S14

S43 S22 OR S23 OR S24 OR S25 OR S26 OR S27 OR S28 OR S29 OR S30 OR S31 OR S32 OR S33 OR S34 OR S35 OR S36 OR S37 OR S38 OR S39 OR S40 OR S41 OR S42

S42intestinal polyp*

S41cecal neoplasm*

S40intestinal adenoma*

S39intestine cancer*

S38bowel cancer*

S37colon neoplasm*

S36colon cancer*

S35colon polyp*

S34colon tumor*

S33colon adenocarcinoma*

S32colon carcinogenesis

S31colon carcinoma*

S30colon adenoma*

S29colorectal cancer*

S28colorectal polyp*

S27colorectal tumor*

S26colorectal adenocarcinoma*

S25colorectal carcinogenesis

S24colorectal carcinoma*

S23colorectal adenoma*

S22colorectal neoplasm*

S21S14 AND S20

S20 S15 OR S16 OR S17 OR S18 OR S19

S19colon cleans*

S18bowel prepa*

S17purgativ*

S16Cathartic*

S15laxativ*

S14 S9 OR S10 OR S11 OR S12 OR S13

S13early intervent*

S12screen*

S11prevent*

S10early diagnos*

S9early detect*

S8 S1 OR S2 OR S3 OR S4 OR S5 OR S6 OR S7

S7polypect*

S6hemoccult*

S5occult blood

S4sigmoidoscop*

S3colonoscop*

S2fobt

S1fecal immunochemical test*

The Cochrane database
Identical to the Pubmed MeSh and Pubmed keyword search

4 Appendix 4 - Reasons for all studies excluded (total list)

Appendix 4: Reasons for all studies excluded at full text level.

Author	Journal	Year	Title	Note
A. T. George, S. Aggarwal, S. Dharmavaram, A. Menon, M. Dube, M. Vogler, et al.	Lancet	2018	Regional variations in UK colorectal cancer screening and mortality	Not fitting defined screening population
Addley, J Mitchell, R M Johnston, S Mainie, I	Ir Med J	2013	Is unsedated colonoscopy the way forward?	Not fitting defined screening population
Akhtar, A. J. Padda, M. S.	Ethn Dis	2011	Safety and efficacy of colonoscopy in the elderly: experience in an innercity community hospital	Mixed population - Data not stratified.

			serving African American and Hispanic patients	
Allen, P.<br>Shaw, E.<br>Jong, A.<br>Behrens, H.<br>Skinner, I.	Journal of Clinical Nursing	2015	Severity and duration of pain after colonoscopy and gastroscopy: a cohort study	Mixed population - Data not stratified.
Alonso, S.<br>Dorcaratto, D.<br>Pera, M.<br>Seoane, A.<br>Dedeu, J. M.<br>Pascual, M.<br>Jose Gil, M.<br>Courtier, R.<br>Bory, F.<br>Grande, L.	Cir Esp	2010	[Incidence of iatrogenic perforation during colonoscopy and their treatment in a university hospital]	Not fitting defined screening population
Anderson, M. L.<br>Pasha, T. M.<br>Leighton, J. A.	Am J Gastroenterol	2000	Endoscopic perforation of the colon: lessons from a 10-year study	Mixed population - Data not stratified
Anna Forsberga, Ulf Hammarb, Anders Ekboma and Rolf Hultcrantzc	SCANDINAVIAN JOURNAL OF GASTROENTEROLOGY	2017	A register-based study: adverse events in colonoscopies performed in Sweden 2001–2013	Not fitting defined screening population
Araghizadeh, F. Y.<br>Timmcke, A. E.<br>Opelka, F. G.<br>Hicks, T. C.<br>Beck, D. E.	Dis Colon Rectum	2001	Colonoscopic perforations	Mixed population - Data not stratified
Aras, A.<br>Oran, E.<br>Seyit, H.<br>Karabulut, M.<br>Gok, I.<br>Alis, H.	Surg Laparosc Endosc Percutan Tech	2016	Colonoscopic Perforations, What is Our Experience in a Training Hospital?	Mixed population - Data not stratified
Araujo, S. E.<br>Seid, V. E.<br>Caravatto, P. P.<br>Dumarco, R.	Hepatogastroenterology	2009	Incidence and management of colonoscopic colon perforations: 10 years' experience	Not fitting defined screening population
Arigbabu, A. O.<br>Odesanmi, W. O.	Dis Colon Rectum	1985	Colonoscopy. First experience in Nigeria	Not fitting defined screening population
Arora, G.<br>Mannalithara, A.<br>Singh, G.<br>Gerson, L. B.<br>Triadafilopoulos, G.	Gastrointest Endosc	2009	Risk of perforation from a colonoscopy in adults: a large population-based study	Mixed population - Data not stratified
Askar Chukmaitov, Bassam Dahman, Cathy J. Bradley	Springer-Verlag GmbH Germany	2019	Outpatient facility volume, facility type, and the risk of serious colonoscopy-related adverse events in patients with comorbid conditions: a population-based study	Not fitting defined screening population

Assaad, B.<br>Sesi, V. K.<br>Figari, R.<br>Schultz, L.<br>Thummala, N.<br>Rehman, M.<br>Chandok, A.<br>Silverman, A.<br>Silver, B.	J Stroke Cerebrovasc Dis	2012	Antithrombotic management of stroke patients before colonoscopy	Mixed population - Data not stratified
Audrius Dulskas, Tomas Poskus, Inga Kildusiene, Ausvydas Patasius, Rokas Stulpinas, Arvydas Laurinavicius, Laura Mašalaite, Gabriele ̇ Milaknyte, Ieva Stundiene, Lina Venceviciene, Kestutis Strupas, Narimantas E. Samalavicius and Giedre Smailyte	MDPI	2021	National Colorectal Cancer Screening Program in Lithuania: Description of the 5-Year Performance on Population Level	No harms investigated
B. Bielawska, L. C. Hookey, R. Sutradhar, M. Whitehead, J. Xu, L. F. Paszat, et al.	Gastroenterology	2018	Anesthesia Assistance in Outpatient Colonoscopy and Risk of Aspiration Pneumonia, Bowel Perforation, and Splenic Injury	Not fitting defined screening population
B. Jahn, G. Sroczynski, M. Bundo, N. Muhlberger, S. Puntscher, J. Todorovic, et al.	BMC Gastroenterol	2019	Effectiveness, benefit harm and cost effectiveness of colorectal cancer screening in Austria	No original data
Baloun, B<br>Amundson, W<br>Khoruts, A	Nature Publishing Group	2011	How painful is a community screening or surveillance colonoscopy?	Mixed population - Data not stratified
Baudet, J. S.<br>Aguirre-Jaime, A.	Endoscopy	2013	Factors related to the development of cat scratch colon during colonoscopy	Not fitting defined screening population
Baudet, J. S.<br>Diaz-Bethencourt, D.<br>Aviles, J.<br>Aguirre-Jaime, A.	European Journal of Gastroenterology Hepatology	2009	Minor adverse events of colonoscopy on ambulatory patients: the impact of moderate sedation	Not fitting defined screening population
Bazalinski, D<br>Kaczmarska, D<br>Bujalski, D	Wspolczesna Onkologia	2010	Pain and anxiety in patients undergoing preventive colon endoscopy	Mixed population - Data not stratified.
Beckers, A. B.<br>Vork, L.<br>Fikree, A.	Therap Adv Gastroenterol	2020	Colonoscopy is safe and not associated with higher pain scores in patients with	Not fitting defined screening population

de Ridder, R.<br>Aziz, Q.<br>Masclee, A.<br>Keszthelyi, D.			hypermobility spectrum disorder: results from an exploratory prospective study	
Benazzato L.; Fedato C.; Fantin A.; Guzzinati S.; Zorzi M.		2018	Analysis of complications after colonoscopy in a population-based colorectal cancer screening programme in veneto.	Conference abstract of another study included
Benter, T.<br>Kurz, C.<br>Schuler, M.<br>Vinis, E.<br>Gottschalk, U.<br>Koop, H.	Dtsch Med Wochenschr	2009	[Splenic injury after colonoscopy: a retrospective study and review of the literature]	Not fitting defined screening population
Berger, D.<br>Boelke, E.<br>Stanescu, A.<br>Buttenschoen, K.<br>Vasilescu, C.<br>Seidelmann, M.<br>Beger, H. G.	Endoscopy	1995	Endotoxemia and mediator release during colonoscopy	Not fitting defined screening population
Bhangu, A.<br>Bowley, D. M.<br>Horner, R.<br>Baranowski, E.<br>Raman, S.<br>Karandikar, S.	British Journal of Surgery	2012	Volume and accreditation, but not specialty, affect quality standards in colonoscopy	Mixed population - Data not stratified.
Biandrate, F.<br>Piccolini, M.<br>Francia, L.<br>Quarone, M.<br>Rosa, C.<br>Battaglia, A.<br>Pandolfi, U.	Chir Ital	2003	[Colonic perforation after colonoscopy: our experience]	Not fitting defined screening population
Blacker, D. J.<br>Wijdicks, E. F.<br>McClelland, R. L.	Neurology	2003	Stroke risk in anticoagulated patients with atrial fibrillation undergoing endoscopy	Not fitting defined screening population
Blotiere, P. O.<br>Weill, A.<br>Ricordeau, P.<br>Alla, F.<br>Allemand, H.	Clin Res Hepatol Gastroenterol	2014	Perforations and haemorrhages after colonoscopy in 2010: a study based on comprehensive French health insurance data (SNIIRAM)	Not fitting defined screening population
Bondonio, A.<br>Sanesi, A.<br>Scarfo, V.<br>Borio, G.<br>Picardi, D.	Minerva Gastroenterol Dietol	1994	[A prospective study on cardiovascular and respiratory complications during colonoscopy]	Specialized endoscopic treatment center
Bowles, C. J.<br>Leicester, R.<br>Romaya, C.<br>Swarbrick, E.	Gut	2004	A prospective study of colonoscopy practice in the UK today: are we adequately prepared for	Not fitting defined screening population

Williams, C. B.<br>Epstein, O.			national colorectal cancer screening tomorrow?	
Braykov, N<br>Iordanov, V L	J buon	2007	Large bowel endoscopy and management of colorectal cancer and its premalignant forms--endoscopy survey	Not fitting defined screening population
Bretthauer, M.<br>Hoff, G.<br>Severinsen, H.<br>Erga, J.<br>Sauar, J.<br>Huppertz-Hauss, G.	Tidsskr Nor Laegeforen	2004	[Systematic quality control programme for colonoscopy in an endoscopy centre in Norway]	Not fitting defined screening population
Britton, D C<br>Tregoning, D<br>Bone, G<br>McKelvey, S T	Br Med J	1977	Colonoscopy in surgical practice	Not fitting defined screening population
Brynitz, S.<br>Kjaergard, H.<br>Struckmann, J.	Ann Chir Gynaecol	1986	Perforations from colonoscopy during diagnosis and treatment of polyps	Not fitting defined screening population
Buron, A<br>Posso, M<br>Sivilla, J<br>Grau, J<br>Guayta, R<br>Castells, X<br>Castells, A<br>Macia, F	Gastroenterol Hepatol	2017	Analysis of participant satisfaction in the Barcelona colorectal cancer screening programme: Positive evaluation of the community pharmacy	No harms investigated
Butt, Sk<br>Defoe, H<br>Besherdas, K	Gut	2012	Quality of flexible sigmoidoscopy for colorectal cancer screening: Are we there yet?	Not fitting defined screening population
Chan, A. O.<br>Lee, L. N.<br>Chan, A. C.<br>Ho, W. N.<br>Chan, Q. W.<br>Lau, S.<br>Chan, J. W.	Hong Kong Med J	2015	Predictive factors for colonoscopy complications	Mixed population - Data not stratified
Chukmaitov, A<br>Siangphoe, U<br>Dahman, B<br>Bradley, C J<br>BouHaidar, D	Dis Colon Rectum	2016	Patient Comorbidity and Serious Adverse Events after Outpatient Colonoscopy: Population-based Study From Three States, 2006 to 2009	Mixed population - Data not stratified.
Chung, Y. W.<br>Han, D. S.<br>Yoo, K. S.<br>Park, C. K.	Dig Liver Dis	2007	Patient factors predictive of pain and difficulty during sedation-free colonoscopy: a prospective study in Korea	Not fitting defined screening population
Cobb, A N<br>Eckerman, A<br>Pruthi, R K	American Society of Hematology	2013	Outcomes of colonoscopies in patients with bleeding disorders	Not fitting defined screening population

Cobb, W. S.<br>Heniford, B. T.<br>Sigmon, L. B.<br>Hasan, R.<br>Simms, C.<br>Kercher, K. W.<br>Matthews, B. D.	Am Surg	2004	Colonoscopic perforations: incidence, management, and outcomes	Mixed population - Data not stratified
Condon, A.<br>Graff, L.<br>Elliot, L.<br>Ilnyckyj, A.	Can J Gastroenterol	2008	Acceptance of colonoscopy requires more than test tolerance	Not fitting defined screening population
Cooper, G. S.<br>Kou, T. D.<br>Rex, D. K.	JAMA Intern Med	2013	Complications following colonoscopy with anesthesia assistance: a population-based analysis	Mixed population - Data not stratified
D. Burtea, A. Dimitriu, A. Maloș, I. Cherciu and A. Săftoiu	Curr Health Sci J	2019	Assessment of the Quality of Outpatient Endoscopic Procedures by Using a Patient Satisfaction Questionnaire	Not fitting defined screening population
Dafnis, G.<br>Ekbom, A.<br>Pahlman, L.<br>Blomqvist, P.	Gastrointest Endosc	2001	Complications of diagnostic and therapeutic colonoscopy within a defined population in Sweden	Not fitting defined screening population
de Jonge, V.<br>Sint Nicolaas, J.<br>van Baalen, O.<br>Brouwer, J. T.<br>Stolk, M. F.<br>Tang, T. J.<br>van Tilburg, A. J.<br>van Leerdam, M. E.<br>Kuipers, E. J.	Am J Gastroenterol	2012	The incidence of 30-day adverse events after colonoscopy among outpatients in the Netherlands	Not fitting defined screening population
Del Río, A S<br>Baudet, J S<br>Fernández, O A<br>Morales, I<br>Socas Mdel, R<br>Del Rio, A S<br>Baudet, J S<br>Fernandez, O A<br>Morales, I<br>Socas Mdel, R	Eur J Gastroenterol Hepatol	2007	Evaluation of patient satisfaction in gastrointestinal endoscopy	Not fitting defined screening population
Denis, B.<br>Weiss, A. M.<br>Peter, A.<br>Bottlaender, J.<br>Chiappa, P.	Gastroenterol Clin Biol	2004	Quality assurance and gastrointestinal endoscopy: an audit of 500 colonoscopic procedures	Mixed population - Data not stratified
Denters, Mj<br>Deutekom, M<br>Fockens, P<br>Bossuyt, Pm<br>Dekker, E	BMC Gastroenterol	2012	Implementation of population screening for colorectal cancer by repeated fecal occult blood test in the Netherlands	Description of trial

deRoux, S. J.<br>Sgarlato, A.	Forensic Sci Med Pathol	2012	Upper and lower gastrointestinal endoscopy mortality: the medical examiner's perspective	Judged equal to case study - not able to assess risk of adverse events
Di Fabio, Francesco<br>Moran, Brendan	Colorectal Disease	2017	Is it time to improve colorectal cancer surveillance from 'one size fits all' to personalized care planning?	No original data/Comparison of technical aspects of interventions/Ongoing trial
Dik, Vk<br>Gralnek, Im<br>Segol, O<br>Suissa, A<br>Moons, Lm<br>Domanov, S<br>Segev, M<br>Rex, Dk<br>Siersema, Pd	Gastroenterology	2014	Comparing standard colonoscopy with endoringsTM colonoscopy: A randomized, multicenter tandem colonoscopy study-interim results of the clever study	Not reporting physical harms
DiPrima, R. E.<br>Barkin, J. S.<br>Blinder, M.<br>Goldberg, R. I.<br>Phillips, R. S.	Am J Gastroenterol	1988	Age as a risk factor in colonoscopy: fact versus fiction	Mixed population - Data not stratified
Dolce, P.<br>Gourdeau, M.<br>April, N.<br>Bernard, P. M.	Am J Infect Control	1995	Outbreak of glutaraldehyde-induced proctocolitis	Not fitting defined screening population
Dorta, G.<br>Hammer, B.	Schweiz Med Wochenschr	1986	[Continuous ECG in colonoscopy]	Not fitting defined screening population
E. Derbyshire, P. Hungin, C. Nickerson and M. D. Rutter	Endoscopy	2017	Post-polypectomy bleeding in the English National Health Service Bowel Cancer Screening Programme	work-up interventions
E. Gorgun, O. Isik, I. Sapci, E. Aytac, M. A. Abbas, G. Ozuner, et al.	Surg Endosc	2018	Colonoscopy-induced acute diverticulitis: myth or reality?	Not fitting defined screening population
E. Swei, J. C. Heller, F. Scott and A. Attwell	Dig Dis Sci	2021	Adverse Event Fatalities Related to GI Endoscopy	Not fitting defined screening population
Eckardt, A. J.<br>Swales, C.<br>Bhattacharya, K.<br>Wassef, W. Y.<br>Phelan, N. P.<br>Zubair, S.<br>Martins, N.<br>Patel, S.<br>Moquin, B.<br>Anwar, N.<br>Leung, K.<br>Levey, J. M.	Endoscopy	2008	Open access colonoscopy in the training setting: which factors affect patient satisfaction and pain?	Mixed population - Data not stratified
Ecker, M D<br>Goldstein, M	Gastroenterology	1977	Benign pneumoperitoneum after fiberoptic	Mixed population - Data not stratified

Hoexter, B<br>Hyman, R A<br>Naidich, J B<br>Stein, H L			colonoscopy. A prospective study of 100 patients	
Ekkelenkamp, V E<br>Dowler, K<br>Valori, R M<br>Dunckley, P	World J Gastroenterol	2013	Patient comfort and quality in colonoscopy	Mixed population - Data not stratified.
Elphick, D. A.<br>Donnelly, M. T.<br>Smith, K. S.<br>Riley, S. A.	Eur J Gastroenterol Hepatol	2009	Factors associated with abdominal discomfort during colonoscopy: a prospective analysis	Not fitting defined screening population
Ettersperger, L.<br>Zeitoun, P.<br>Thiefin, G.	Gastroenterol Clin Biol	1995	[Colonic perforations complicating colonoscopy. Apropos of 15 consecutive cases observed over 16 years]	Not fitting defined screening population
Feagins, L. A.<br>Iqbal, R.<br>Harford, W. V.<br>Halai, A.<br>Cryer, B. L.<br>Dunbar, K. B.<br>Davila, R. E.<br>Spechler, S. J.	Clin Gastroenterol Hepatol	2013	Low rate of postpolypectomy bleeding among patients who continue thienopyridine therapy during colonoscopy	Mixed population - Data not stratified
Feagins, L. A.<br>Uddin, F. S.<br>Davila, R. E.<br>Harford, W. V.<br>Spechler, S. J.	Digestive Diseases and Sciences	2011	The rate of post-polypectomy bleeding for patients on uninterrupted clopidogrel therapy during elective colonoscopy is acceptably low	Mixed population - Data not stratified
Fennerty, M. B.<br>Earnest, D. L.<br>Hudson, P. B.<br>Sampliner, R. E.	Gastrointest Endosc	1990	Physiologic changes during colonoscopy	Mixed population - Data not stratified
Forsberg, Anna M<br>Hammar, Ulf<br>Ekbom, Anders<br>Hultcrantz, Rolf	Gastrointest Endosc	2016	Is the Risk of Serious Adverse Events in Colonoscopy Underestimated: Results From a Large Swedish Population-Based Study	Mixed population - Data not stratified
Fox, Elaine<br>O'Boyle, Ciaran A<br>Lennon, John	Stress medicine	1987	Stress responses to two invasive medical investigations: Left-sided colonoscopy and sigmoidoscopy	Mixed population - Data not stratified
Fox, J. P.<br>Burkardt, D. D.<br>Ranasinghe, I.<br>Gross, C. P.	Med Care	2014	Hospital-based acute care after outpatient colonoscopy: implications for quality measurement in the ambulatory setting	Not fitting defined screening population
Fruhmorgen, P<br>Demling, L	Endoscopy	1979	Complications of diagnostic and therapeutic colonoscopy in the Federal	Not fitting defined screening population

			Republic of Germany. Results of an inquiry	
Fukuzawa, M.<br>Uematsu, J.<br>Kono, S.<br>Suzuki, S.<br>Sato, T.<br>Yagi, N.<br>Tsuji, Y.<br>Yagi, K.<br>Kusano, C.<br>Gotoda, T.<br>Kawai, T.<br>Moriyasu, F.	World J Gastroenterol	2015	Clinical impact of endoscopy position detecting unit (UPD-3) for a non-sedated colonoscopy	Mixed population - Data not stratified.
Garbay, J. R.<br>Suc, B.<br>Rotman, N.<br>Fourtanier, G.<br>Escat, J.	British journal of surgery	1996	Multicentre study of surgical complications of colonoscopy	Not fitting defined screening population
Garcia Martinez, M. T.<br>Ruano Poblador, A.<br>Galan Raposo, L.<br>Gay Fernandez, A. M.<br>Casal Nunez, J. E.	Revista Espanola De Enfermedades Digestivas	2007	[Perforation after colonoscopy: our 16-year experience]	Not fitting defined screening population
Gatto, N. M.<br>Frucht, H.<br>Sundararajan, V.<br>Jacobson, J. S.<br>Grann, V. R.<br>Neugut, A. I.	J Natl Cancer Inst	2003	Risk of perforation after colonoscopy and sigmoidoscopy: a population-based study	Mixed population - Data not stratified.
Gavaruzzi, T.<br>Carnaghi, A.<br>Lotto, L.<br>Rumiati, R.<br>Meggiato, T.<br>Polato, F.<br>De Lazzari, F.	British journal of health psychology	2010	Recalling pain experienced during a colonoscopy: pain expectation and variability	Mixed population - Data not stratified.
Gavin, D. R.<br>Valori, R. M.<br>Anderson, J. T.<br>Donnelly, M. T.<br>Williams, J. G.<br>Swarbrick, E. T.	Gut	2013	The national colonoscopy audit: a nationwide assessment of the quality and safety of colonoscopy in the UK	Mixed population - Data not stratified.
Geenen, J. E.<br>Schmitt, M. G., Jr.<br>Wu, W. C.<br>Hogan, W. J.	The American Journal of Digestive Diseases	1975	Major complications of coloscopy: bleeding and perforation	Not fitting defined screening population
Gerson, L B<br>Michaels, L<br>Ullah, N<br>Gage, B<br>Williams, L	Gastrointest Endosc	2010	Adverse events associated with anticoagulation therapy in the periendoscopic period	Not fitting defined screening population

Gibbs, D. H. Opelka, F. G. Beck, D. E. Hicks, T. C. Timmcke, A. E. Gathright, J. B., Jr.	Dis Colon Rectum	1996	Postpolypectomy colonic hemorrhage	Not fitting defined screening population
Goldman, G. D. Miller, S. A. Furman, D. S. Brock, D. Ryan, J. L. McCallum, R. W.	Am J Gastroenterol	1985	Does bacteremia occur during flexible sigmoidoscopy?	Mixed population - Data not stratified
Grasset, D. Morfoisse, J. J. Seigneuric, C.	Gastroenterol Clin Biol	2000	[Conditions of practice and results of colonoscopy in non-university hospitals. Results of a cross sectional, multicenter ANGH study (1)]	Mixed population - Data not stratified
Gregorios A. Paspatis, Maria Fragaki, Magdalini Velegraki, Afroditi Mpitouli, Pinelopi Nikolaou, Georgios Tribonias, Evangelos Voudoukis, Konstantinos Karmiris, Angeliki Theodoropoulou, Emmanouil Vardas	Thieme	2021	Paradigm shift in management of acute iatrogenic colonic perforations: 24-year retrospective comprehensive study	Not fitting defined screening population
Guihan, M Hayman, A V Fisher, M J Anaya, B C Parachuri, R Bentrem, D J	Topics in Spinal Cord Injury Rehabilitation	2011	Colonoscopy is safe for veterans with spinal cord injury or disorder (SCI&D)	Not fitting defined screening population
Gupta, S. C. Gopalswamy, N. Sarkar, A. Suryaprasad, A. G. Markert, R. J.	Mil Med	1990	Cardiac arrhythmias and electrocardiographic changes during upper and lower gastrointestinal endoscopy	Mixed population - Data not stratified
H. J. Goong, B. M. Ko and M. S. Lee	Dig Dis Sci	2021	Adverse Events Associated with Routine Colonoscopy in Patients with End-Stage Renal Disease	Not fitting defined screening population
H. Saito, S. E. Kudo, N. Takahashi, S. Yamamoto, K. Kodama, K. Nagata, et al.	Int J Colorectal Dis	2020	Efficacy of screening using annual fecal immunochemical test alone versus combined with one-time colonoscopy in reducing colorectal cancer mortality: the Akita Japan population-based	Ongoing trial

			colonoscopy screening trial (Akita pop-colon trial)	
Hagel, A. F.<br>Boxberger, F.<br>Dauth, W.<br>Kessler, H. P.<br>Neurath, M. F.<br>Raithel, M.	Colorectal Dis	2012	Colonoscopy-associated perforation: a 7-year survey of in-hospital frequency, treatment and outcome in a German university hospital	Not fitting defined screening population
Halter, F.	Schweiz Med Wochenschr	1978	[Indications for and dangers of methods for gastroenterological endoscopy]	Not fitting defined screening population
Hayman, A. V.<br>Guihan, M.<br>Fisher, M. J.<br>Murphy, D.<br>Anaya, B. C.<br>Parachuri, R.<br>Rogers, T. J.<br>Bentrem, D. J.	J Spinal Cord Med	2013	Colonoscopy is high yield in spinal cord injury	Mixed population - Data not stratified.
Herman, L. L.<br>Kurtz, R. C.<br>McKee, K. J.<br>Sun, M.<br>Thaler, H. T.<br>Winawer, S. J.	Gastrointest Endosc	1993	Risk factors associated with vasovagal reactions during colonoscopy	Not fitting defined screening population
Heuss, L. T.<br>Sughanda, S. P.<br>Degen, L. P.	Swiss Med Wkly	2012	Endoscopy teams' judgment of discomfort among patients undergoing colonoscopy: "How bad was it really?"	Mixed population - Data not stratified.
Hilsden, R J<br>Dube, C<br>Heitman, S J<br>Bridges, R<br>McGregor, S E<br>Rostom, A	Gastrointest Endosc	2015	The association of colonoscopy quality indicators with the detection of screen-relevant lesions, adverse events, and postcolonoscopy cancers in an asymptomatic Canadian colorectal cancer screening population	Mixed population - Data not stratified.
Hoff, G.<br>Thiis-Evensen, E.<br>Grotmol, T.<br>Sauar, J.<br>Vatn, M. H.<br>Moen, I. E.	Eur J Cancer Prev	2001	Do undesirable effects of screening affect all-cause mortality in flexible sigmoidoscopy programmes? Experience from the Telemark Polyp Study 1983-1996	Unclear which interventions people receive
Hoff, Geir<br>de Lange, Thomas<br>Bretthauer, Michael<br>Buset, Magne<br>Dahler, Stein<br>Halvorsen, Fred-Arne A.	Endoscopy	2017	Patient-reported adverse events after colonoscopy in Norway	Not fitting defined screening population

Halwe, Jörg Michael<br>Heibert, Mathis<br>Høie, Ole<br>Kjellevold, Øystein<br>Moritz, Volker<br>Sandvei, Per<br>Seip, Birgitte<br>Aabakken, Lars<br>Holme, Øyvind				
Hoffman, M. S.<br>Butler, T. W.<br>Shaver, T.	J Clin Gastroenterol	1998	Colonoscopy without sedation	Not fitting defined screening population
Holm, C.<br>Christensen, M.<br>Rasmussen, V.<br>Schulze, S.<br>Rosenberg, J.	Scand J Gastroenterol	1998	Hypoxaemia and myocardial ischaemia during colonoscopy	Not fitting defined screening population
Holme, O.<br>Bretthauer, M.<br>de Lange, T.<br>Seip, B.<br>Huppertz-Hauss, G.<br>Hoie, O.<br>Sandvei, P.<br>Ystrom, C. M.<br>Hoff, G.	Endoscopy	2013	Risk stratification to predict pain during unsedated colonoscopy: results of a multicenter cohort study	Not fitting defined screening population
Holme, O.<br>Moritz, V.<br>Bretthauer, M.<br>Seip, B.<br>Glomsaker, T.<br>de Lange, T.<br>Aabakken, L.<br>Stallemo, A.<br>Hoie, O.<br>Dahler, S.<br>Sandvei, P. K.<br>Stray, N.<br>Ystrom, C. M.<br>Hoff, G.	Tidsskr Nor Laegeforen	2013	[Pain in connection with colonoscopy in Norway]	Not fitting defined screening population
Holme, O.<br>Hoie, O.<br>Matre, J.<br>Stallemo, A.<br>Garborg, K.<br>Hasund, A.<br>Wiig, H.<br>Hoff, G.<br>Bretthauer, M.	Gastrointest Endosc	2011	Magnetic endoscopic imaging versus standard colonoscopy in a routine colonoscopy setting: a randomized, controlled trial	Not fitting defined screening population
Hsieh, T. K.<br>Hung, L.<br>Kang, F. C.<br>Lan, K. M.<br>Poon, P. W.<br>So, E. C.	Acta Anaesthesiol Taiwan	2009	Anesthesia does not increase the rate of bowel perforation during colonoscopy: a retrospective study	Not fitting defined screening population

Hull, T.<br>Church, J. M.	Surg Endosc	1994	Colonoscopy--how difficult, how painful?	Not fitting defined screening population
Imai, N.<br>Takeda, K.<br>Kuzuya, T.<br>Utsunomiya, S.<br>Takahashi, H.<br>Kasuga, H.<br>Asai, M.<br>Yamada, M.<br>Tanikawa, Y.<br>Goto, H.	Clin Gastroenterol Hepatol	2010	High incidence of colonic perforation during colonoscopy in hemodialysis patients with end-stage renal disease	Not fitting defined screening population
Iqbal, C. W.<br>Cullinane, D. C.<br>Schiller, H. J.<br>Sawyer, M. D.<br>Zietlow, S. P.<br>Farley, D. R.	Archives of Surgery	2008	Surgical management and outcomes of 165 colonoscopic perforations from a single institution	Not fitting defined screening population
Isbister, W H	Aust N Z J Surg	1986	Colorectal polyps: an endoscopic experience	Not fitting defined screening population
J. Lachter, H. Nickowitz and E. E. Half	Gastrointestinal Endoscopy	2020	POST-COLONOSCOPY COMPLICATIONS WITHIN ONE MONTH; PATIENT SATISFACTION, AND PATIENT EXPERIENCE	Mixed population
Jaroslaw Regula, M.D., Maciej Rupinski, M.D., Ewa Kraszewska, M.Sc.,<br>Marcin Polkowski, M.D., Jacek Pachlewski, M.D., Janina Orlowska, M.D.,<br>Marek P. Nowacki, M.D., and Eugeniusz Butruk, M.D.	The new england journal o f medicine	2006	Colonoscopy in Colorectal-Cancer Screening<br>for Detection of Advanced Neoplasia	high risk - 20% family history of colorectal cancer
Jelsness-Jorgensen, L. P.<br>Lerang, F.<br>Sandvei, P.<br>Soberg, T.<br>Henriksen, M.	Scand J Gastroenterol	2013	Magnetic endoscopic imaging during colonoscopy is associated with less pain and decreased need of analgesia and sedation--results from a randomized controlled trial	Not fitting defined screening population
Jovanovic, I.<br>Zimmermann, L.<br>Fry, L. C.<br>Monkemuller, K.	Gastrointest Endosc	2011	Feasibility of endoscopic closure of an iatrogenic colon perforation occurring during colonoscopy	Mixed population - Data not stratified.

K. A. Lambert, A. Hamed and A. Hamed	American Family Physician	2019	Flexible sigmoidoscopy or fecal occult blood testing for colorectal cancer screening in asymptomatic adults	Review
Kamath, A. S.<br>Iqbal, C. W.<br>Sarr, M. G.<br>Cullinane, D. C.<br>Zietlow, S. P.<br>Farley, D. R.<br>Sawyer, M. D.	J Gastrointest Surg	2009	Colonoscopic splenic injuries: incidence and management	Not fitting defined screening population
Kang, H. Y.<br>Kang, H. W.<br>Kim, S. G.<br>Kim, J. S.<br>Park, K. J.<br>Jung, H. C.<br>Song, I. S.	Digestion	2008	Incidence and management of colonoscopic perforations in Korea	Not fitting defined screening population
Karajeh, M. A.<br>Sanders, D. S.<br>Hurlstone, D. P.	Endoscopy	2006	Colonoscopy in elderly people is a safe procedure with a high diagnostic yield: a prospective comparative study of 2000 patients	Not fitting defined screening population
Kelley, C. J.<br>Ingoldby, C. J.<br>Blenkharn, J. I.<br>Wood, C. B.	Surg Gynecol Obstet	1985	Colonoscopy related endotoxemia	Not fitting defined screening population
Ker, T. S.<br>Wasserberg, N.<br>Beart, R. W., Jr.	The American Journal of Surgery	2004	Colonoscopic perforation and bleeding of the colon can be treated safely without surgery	Not fitting defined screening population
Kilaru, S M<br>Oza, S S<br>Gautam, S<br>Wolf, J L	Colonoscopies are not safe in patients with type 4 ehlers-danlos syndrome but appear to be safe with minimally increased rate of perforation in patients with other types of ehlers-danlos or marfan syndrome	2015	Gastroenterology	Not fitting defined screening population
Kim, D H<br>Lee, S Y<br>Choi, K S<br>Lee, H J<br>Park, S C<br>Kim, J<br>Han, C J<br>Kim, Y C	The usefulness of colonoscopy as a screening test for detecting colorectal polyps	2007	Hepatogastroenterology	No harms investigated
Kim, H H<br>Kye, B H<br>Kim, H J<br>Cho, H M	Annals of Coloproctology	2014	Prompt management is most important for colonic perforation after colonoscopy	Not fitting defined screening population
Kim, H. S.<br>Kim, T. I.	American journal of gastroenterology	2003	Risk factors for immediate postpolypectomy bleeding	Not fitting defined screening population

Kim, W. H.<br>Kim, Y. H.<br>Kim, H. J.<br>Yang, S. K.<br>Myung, S. J.<br>Byeon, J. S.<br>Lee, M. S.<br>Chung, I. K.<br>Jung, S. A.<br>Jeen, Y. T.<br>Choi, J. H.<br>Choi, K. Y.<br>Choi, H.<br>Han, D. S.<br>Song, J. S.			of the colon: a multicenter study	
Kim, W. H.<br>Cho, Y. J.<br>Park, J. Y.<br>Min, P. K.<br>Kang, J. K.<br>Park, I. S.	Gastrointest Endosc	2000	Factors affecting insertion time and patient discomfort during colonoscopy	Mixed population - Data not stratified
Kirby, E.	Can J Rural Med	2004	Colonoscopy procedures at a small rural hospital	Mixed population - Data not stratified
Kiss, A.<br>Ferenci, P.<br>Graninger, W.<br>Pamperl, H.<br>Potzi, R.<br>Meryn, S.	Endoscopy	1983	Endotoxaemia following colonoscopy	Not fitting defined screening population
Ko, C. W.<br>Riffle, S.<br>Michaels, L.<br>Morris, C.<br>Holub, J.<br>Shapiro, J. A.<br>Ciol, M. A.<br>Kimmey, M. B.<br>Seeff, L. C.<br>Lieberman, D.	Clin Gastroenterol Hepatol	2010	Serious complications within 30 days of screening and surveillance colonoscopy are uncommon	Mixed population - Data not stratified.
Ko, C. W.<br>Riffle, S.<br>Shapiro, J. A.<br>Saunders, M. D.<br>Lee, S. D.<br>Tung, B. Y.<br>Kuver, R.<br>Larson, A. M.<br>Kowdley, K. V.<br>Kimmey, M. B.	Gastrointest Endosc	2007	Incidence of minor complications and time lost from normal activities after screening or surveillance colonoscopy	Mixed population - Data not stratified
Kolber, M R<br>Wong, C K<br>Fedorak, R N<br>Rowe, B H	PLoS One	2013	Prospective Study of the Quality of Colonoscopies Performed by Primary Care Physicians: The Alberta Primary Care Endoscopy (APC-Endo) Study	Mixed population - Data not stratified.

Korman, L. Y.<br>Overholt, B. F.<br>Box, T.<br>Winker, C. K.	Gastrointest Endosc	2003	Perforation during colonoscopy in endoscopic ambulatory surgical centers	Mixed population - Data not stratified.
Krishna, S. G.<br>Rao, B. B.<br>Thirumurthi, S.<br>Lee, J. H.<br>Ramireddy, S.<br>Guindani, M.<br>Ross, W. A.	Gastrointest Endosc	2014	Safety of endoscopic interventions in patients with thrombocytopenia	Not fitting defined screening population
Kronborg, O<br>Fenger, C<br>Worm, J<br>Pedersen, S A<br>Hem, J<br>Bertelsen, K<br>Olsen, J	Scand J Gastroenterol	1992	Causes of death during the first 5 years of a randomized trial of mass screening for colorectal cancer with fecal occult blood test	No harms investigated
Kumar, S.<br>Abcarian, H.<br>Prasad, M. L.<br>Lakshmanan, S.	Dis Colon Rectum	1983	Bacteremia associated with lower gastrointestinal endoscopy: fact or fiction? II. Proctosigmoidoscopy	Not fitting defined screening population
Kumar, S.<br>Abcarian, H.<br>Prasad, M. L.<br>Lakshmanan, S.	Dis Colon Rectum	1982	Bacteremia associated with lower gastrointestinal endoscopy, fact or fiction? I. Colonoscopy	Not fitting defined screening population
L. B. Grossberg, K. Papamichael, D. A. Leffler, M. S. Sawhney and J. D. Feuerstein	Dig Dis Sci	2021	Patients over Age 75 Are at Increased Risk of Emergency Department Visit and Hospitalization Following Colonoscopy	Mixed population
La Torre, M.<br>Velluti, F.<br>Giuliani, G.<br>Di Giulio, E.<br>Ziparo, V.<br>La Torre, F.	Colorectal Disease	2012	Promptness of diagnosis is the main prognostic factor after colonoscopic perforation	Mixed population - Data not stratified.
Ladabaum, Uri	Endoscopy	2021	Doing our best to do no harm	No original data/Comparison of technical aspects of interventions/Ongoing trial
Lazovic, R.<br>Krivokapic, Z.	Acta Chir Iugosl	2004	[Endoscopic perforations of colona and rectum]	Not fitting defined screening population
Lee, Y. C.<br>Wang, H. P.<br>Chiu, H. M.<br>Lin, C. P.<br>Huang, S. P.<br>Lai, Y. P.<br>Wu, M. S.<br>Chen, M. F.<br>Lin, J. T.	J Gastroenterol Hepatol	2006	Factors determining post-colonoscopy abdominal pain: prospective study of screening colonoscopy in 1000 subjects	Mixed population - data not stratified
Levin, T. R.<br>Zhao, W.<br>Conell, C.<br>Seeff, L. C.	Ann Intern Med	2006	Complications of colonoscopy in an integrated health care delivery system	Mixed population - data not stratified

Manninen, D. L.<br>Shapiro, J. A.<br>Schulman, J.				
Lieberman, D A<br>Faigel, D O<br>Logan, J R<br>Mattek, N<br>Holub, J<br>Eisen, G<br>Morris, C<br>Smith, R<br>Nadel, M	Gastrointest Endosc	2009	Assessment of the quality of colonoscopy reports: results from a multicenter consortium	Not fitting defined screening population
Lippert, E.<br>Herfarth, H. H.<br>Grunert, N.<br>Endlicher, E.<br>Klebl, F.	Int J Colorectal Dis	2015	Gastrointestinal endoscopy in patients aged 75 years and older: risks, complications, and findings--a retrospective study	Not fitting defined screening population
Llach, J.<br>Elizalde, J. I.<br>Bordas, J. M.<br>Gines, A.<br>Almela, M.<br>Sans, M.<br>Mondelo, F.<br>Pique, J. M.	Gastrointest Endosc	1999	Prospective assessment of the risk of bacteremia in cirrhotic patients undergoing lower intestinal endoscopy	Not fitting defined screening population
Lo, A. Y.<br>Beaton, H. L.	Journal of the American College of Surgeons	1994	Selective management of colonoscopic perforations	Not fitting defined screening population
Lohsiriwat, V.<br>Sujarittanakarn, S.<br>Akaraviputh, T.<br>Lertakyamanee, N.<br>Lohsiriwat, D.<br>Kachinthorn, U.	BMC gastroenterology	2009	What are the risk factors of colonoscopic perforation?	Mixed population - Data not stratified.
Lohsiriwat, V.<br>Sujarittanakarn, S.<br>Akaraviputh, T.<br>Lertakyamanee, N.<br>Lohsiriwat, D.<br>Kachinthorn, U.	World J Gastroenterol	2008	Colonoscopic perforation: A report from World Gastroenterology Organization endoscopy training center in Thailand	Not fitting defined screening population
London, M. T.<br>Chapman, B. A.<br>Faoagali, J. L.<br>Cook, H. B.	New Zealand Medical Journal	1986	Colonoscopy and bacteraemia: an experience in 50 patients	Not fitting defined screening population
Lorenzo-Zuniga, V.<br>Moreno de Vega, V.<br>Domenech, E.<br>Manosa, M.<br>Planas, R.<br>Boix, J.	Colorectal Dis	2010	Endoscopist experience as a risk factor for colonoscopic complications	Not fitting defined screening population
Low, D. E.<br>Shoenut, J. P.<br>Kennedy, J. K.<br>Sharma, G. P.	Digestive Diseases and Sciences	1987	Prospective assessment of risk of bacteremia with colonoscopy and polypectomy	Not fitting defined screening population

Harding, G. K.<br>Den Boer, B.<br>Micflikier, A. B.				
Luning, T. H.<br>Keemers-Gels, M. E.<br>Barendregt, W. B.<br>Tan, A. C.<br>Rosman, C.	Surg Endosc	2007	Colonoscopic perforations: a review of 30,366 patients	Not fitting defined screening population
Laanani, M.<br>Weill, A.<br>Carbonnel, F.<br>Pouchot, J.<br>Coste, J.	Am J Gastroenterol	2020	Incidence of and Risk Factors for Systemic Adverse Events After Screening or Primary Diagnostic Colonoscopy: A Nationwide Cohort Study	Not fitting defined screening population
M. Hoffmeister	The Lancet Gastroenterology and Hepatology	2022	Interim evaluation of the colorectal cancer screening programme in the Netherlands	No original data
M. J. Sewitch, V. Marquez Azalgara, M. Fon Sing, V. M. Azalgara and M. F. Sing	Gastroenterology nursing	2018	Screening Indication Associated With Lower Likelihood of Minor Adverse Events in Patients Undergoing Outpatient Colonoscopy	Mixed population
M. Laanani, A. Weill, P. O. Blotiere, J. Pouchot, F. Carbonnel and J. Coste	United European Gastroenterology Journal	2019	Factors associated with mechanical and systemic adverse events after colonoscopy (France , 2010-2015)	Not fitting defined screening population
M. Laanani, J. Coste, P. O. Blotière, F. Carbonnel and A. Weill	Clin Gastroenterol Hepatol	2019	Patient, Procedure, and Endoscopist Risk Factors for Perforation, Bleeding, and Splenic Injury After Colonoscopies	Mixed population
M. M. Khaing, P. Kellar, F. Hartnell, J. Croese, R. Hodgson, T. James, et al.	Future Healthc J	2020	Patient reported outcome measures (PROMS) - 30-day mortality and adverse events post colonoscopy: A prospective observational study from a metropolitan Australian hospital	Not fitting defined screening population
Macrae, F. A.<br>Tan, K. G.<br>Williams, C. B.	Gut	1983	Towards safer colonoscopy: a report on the complications of 5000 diagnostic or therapeutic colonoscopies	Mixed population - Data not stratified.
Mai, C. M.<br>Wen, C. C.<br>Wen, S. H.<br>Hsu, K. F.<br>Wu, C. C.	Int J Colorectal Dis	2010	Iatrogenic colonic perforation by colonoscopy: a fatal complication for patients with a high anesthetic risk	Not fitting defined screening population

Jao, S. W.<br>Hsiao, C. W.				
Mansmann, U<br>Crispin, A<br>Henschel, V<br>Adrion, C<br>Augustin, V<br>Birkner, B<br>Munte, A	Deutsches Arzteblatt	2008	Epidemiology and quality control of 245 000 outpatient colonoscopies	Mixed population - Data not stratified.
Marino, M<br>Berretti, D<br>Maieron, R<br>Rossitti, P<br>Zilli, M	Gut	2011	Colonoscopic adverse events during bowel cancer screening programme in north-east Italy: A preliminary report	Preliminary report of another study included
Marquez Azalgara, V.<br>Sewitch, M. J.<br>Joseph, L.<br>Barkun, A. N.	Can J Gastroenterol Hepatol	2014	Rates of minor adverse events and health resource utilization postcolonoscopy	Mixed population - Data not stratified
Matharoo, G. S.<br>Goldfarb, M. A.	Am Surg	2012	Treatment and outcomes of iatrogenic colon perforations at a community teaching hospital	Mixed population - Data not stratified
McCarthy, B. D.<br>Moskowitz, M. A.	J Gen Intern Med	1993	Screening flexible sigmoidoscopy: patient attitudes and compliance	Mixed population - Data not stratified
Meester, R. G.<br>Doubeni, C. A.<br>Lansdorp-Vogelaar, I.<br>Jensen, C. D.<br>van der Meulen, M. P.<br>Levin, T. R.<br>Quinn, V. P.<br>Schottinger, J. E.<br>Zauber, A. G.<br>Corley, D. A.<br>van Ballegooijen, M.	JAMA	2015	Variation in Adenoma Detection Rate and the Lifetime Benefits and Cost of Colorectal Cancer Screening: A Microsimulation Model	No original data
Miller, R. E.<br>Bossart, P. M.<br>Tiszenkel, H. I.<br>Kimmelstiel, F. M.	Surg Laparosc Endosc Percutan Tech	1991	Surgical management of complications of fiberoptic colonoscopy	Not fitting defined screening population
Misra, T.<br>Lalor, E.<br>Fedorak, R. N.	Can J Gastroenterol	2004	Endoscopic perforation rates at a Canadian university teaching hospital	Not fitting defined screening population
Morgan, J.<br>Roufeil, L.<br>Kaushik, S.<br>Bassett, M.	Gastrointest Endosc	1998	Influence of coping style and precolonoscopy information on pain and anxiety of colonoscopy	Not fitting defined screening population
Moser, B.	Leber Magen Darm	1987	[Colonoscopy in advanced age: cardiac arrhythmias]	Not fitting defined screening population

Mukewar, S.<br>Costedio, M.<br>Wu, X.<br>Bajaj, N.<br>Lopez, R.<br>Brzezinski, A.<br>Shen, B.	Inflammatory Bowel Diseases	2014	Severe adverse outcomes of endoscopic perforations in patients with and without IBD	Not fitting defined screening population
N. Lahodzich, A. Varabei and A. Dybau	Colorectal Disease	2019	Complications of colonoscopic polypectomy: Experience of one center	Not fitting defined screening population
N. Merza, B. P. Rutledge, A. Al-Hillan, M. Shaaeli, M. Saadaldin and T. Naguib	American Journal of Gastroenterology	2021	Colonoscopy adverse events in urban versus rural hospitals: Retrospective cohort analysis from nationwide data	Not fitting defined screening population
Nahas, S C<br>Bringel, R W<br>Sobrado Junior, C W<br>Nahas, C S<br>Borba, M R<br>Araujo, S E<br>Habr-Gama, A	Arq gastroenterology	1999	Diagnostic colonoscopy	Mixed population - Data not stratified
Naumann, D. N.<br>Potter-Concannon, S.<br>Karandikar, S.	Frontline Gastroenterol	2019	interobserver variability in comfort scores for screening colonoscopy	No harms investigated
Nelson, R. L.<br>Abcarian, H.<br>Prasad, M. L.	Dis Colon Rectum	1982	Iatrogenic perforation of the colon and rectum	Not fitting defined screening population
Nicholson, F. B.<br>Korman, M. G.	J Med Screen	2005	Acceptance of flexible sigmoidoscopy and colonoscopy for screening and surveillance in colorectal cancer prevention	Mixed population - Data not stratified
Nijjar, U K<br>Edwards, J A<br>Short, M W	J Am Board Fam Med	2011	Patient satisfaction with family physician colonoscopists	Mixed population - Data not stratified
Niv, Y.<br>Bogolavski, I.<br>Ilani, S.<br>Avni, I.<br>Gal, E.<br>Vilkin, A.<br>Levi, Z.'	Eur J Gastroenterol Hepatol	2012	Impact of colonoscopy on quality of life	Mixed population - Data not stratified
Niv, Y.<br>Gershtansky, Y.<br>Kenett, R. S.<br>Tal, Y.<br>Birkenfeld, S.	Eur J Gastroenterol Hepatol	2011	Complications in colonoscopy: analysis of 7-year physician-reported adverse events	Not fitting defined screening population

Norfleet, R. G.	Gastrointest Endosc	1982	Colonoscopy and polypectomy in nonhospitalized patients	Not fitting defined screening population
Novis, B H<br>Bank, S<br>Brown, A A	South African Medical Journal	1976	A review of colonoscopy in 200 patients	Mixed population - Data not stratified
Omar Sadiq, Maryam Alimirah, Sandra Naffouj, Firas Askar, Yousuf Siddiqui, Raxitkumar Jinjuvadia	The American Journal of GASTROENTEROLOGY	2019	Increased Frequency of Colonoscopy Complications in Patients With Positive Multitarget Stool DNA Testing for Colorectal Cancer Screening	unconventional screening intervention
Ono, S.<br>Fujishiro, M.<br>Kodashima, S.<br>Takahashi, Y.<br>Minatsuki, C.<br>Mikami-Matsuda, R.<br>Asada-Hirayama, I.<br>Konno-Shimizu, M.<br>Tsuji, Y.<br>Mochizuki, S.<br>Niimi, K.<br>Yamamichi, N.<br>Kaneko, M.<br>Yatomi, Y.<br>Koike, K.	J Gastroenterol	2012	Evaluation of safety of endoscopic biopsy without cessation of antithrombotic agents in Japan	Not fitting defined screening population
Orsoni, P.<br>Berdah, S.<br>Verrier, C.<br>Caamano, A.<br>Sastre, B.<br>Boutboul, R.<br>Grimaud, J. C.<br>Picaud, R.	Endoscopy	1997	Colonic perforation due to colonoscopy: a retrospective study of 48 cases	Not fitting defined screening population
Ostborg, J.<br>Skjolingstad, R.<br>Bakkevold, K.	Tidsskrift for den Norske laegeforening : tidsskrift for praktisk medicin, ny raekke	1994	[Quality control and use of resources in colonoscopy. A prospective evaluation of 1000 colonoscopies]	Mixed population - Data not stratified
Ozsoy, M<br>Celep, B<br>Ersen, O<br>Ozkececi, T<br>Bal, A<br>Yilmaz, S<br>Arikan, Y	Ulus Cerrahi Derg	2014	Our results of lower gastrointestinal endoscopy: evaluation of 700 patients	Not fitting defined screening population
Park, D. I.<br>Kim, H. J.<br>Park, J. H.<br>Cho, Y. K.<br>Sohn, C. I.	Eur J Gastroenterol Hepatol	2007	Factors affecting abdominal pain during colonoscopy	Not fitting defined screening population

Jeon, W. K.<br>Kim, B. I.<br>Ryu, S. H.<br>Sung, I. K.				
Paspatis, G. A.<br>Vardas, E.<br>Theodoropoulou, A.<br>Manolaraki, M. M.<br>Charoniti, I.<br>Papanikolaou, N.<br>Chroniaris, N.<br>Chlouverakis, G.	Dig Liver Dis	2008	Complications of colonoscopy in a large public county hospital in Greece. A 10-year study	Mixed population - Data not stratified
Pearl, J. P.<br>McNally, M. P.<br>Elster, E. A.<br>DeNobile, J. W.	Mil Med	2006	Benign pneumoperitoneum after colonoscopy: a prospective pilot study	Mixed population - Data not stratified
Plummer, J. M.<br>Mitchell, D. I.<br>Ferron-Boothe, D.<br>Meeks-Aitken, N.<br>Reid, M.	West Indian Med J	2012	Colonoscopy in central Jamaica: results and implications	Mixed population - Data not stratified
Poddutoori, Padma<br>Gachpaz, Babak<br>Duvvuri, Abhiram<br>Vennelaganti, Sreekar<br>Jegadeesan, Ramprasad<br>Vennalaganti, Prashanth<br>Aziz, Muhammad<br>Vittal, Anusha<br>Singh, Pratiksha<br>Kennedy, Kevin F.<br>Rai, Tarun<br>Parasa, Sravanthi<br>Choudhary, Abhishek<br>Bansal, Ajay<br>Gupta, Neil<br>Sharma, Prateek	Gastrointestinal Endoscopy	2017	Sa1059 Low Rates of 30-Day Post Colonoscopy Related Major Adverse Events: Data From More Than 5000 Procedures in a Large Tertiary Care Medical Center...Digestive Disease Week (DDW) 2017 American Society for Gastrointestinal Endoscopy (ASGE) Program and Abstracts, Chicago, Illinois, 6–9 May 2017	Not fitting defined screening population
R. B. Coser, M. B. Dalio, L. C. P. Martins, G. F. Alvarenga, C. A. Cruz, A. R. Imperiale, et al	Rev Col Bras Cir	2018	Colonoscopy complications: experience with 8968 consecutive patients in a single institution	Not fitting defined screening population
R. Jover	Endoscopy	2017	Colorectal cancer screening: with pain, no gain	No original data
R. Kumar and N. Choudhary	Am Surg	2018	Colonoscopy in Rural General Surgery Practice: Challenges, Outcomes, and Quality Measures	General practice

R. Ri Cha, C. Yoon Ha, H. Jin Kim and O.-J. Lee	Journal of Gastroenterology and Hepatology	2019	Clinical characteristics and outcome of iatrogenic colonic perforation related to diagnostic vs. therapeutic colonoscopy	Not fitting defined screening population
Rabeneck, L.<br>Paszat, L. F.<br>Hilsden, R. J.<br>Saskin, R.<br>Leddin, D.<br>Grunfeld, E.<br>Wai, E.<br>Goldwasser, M.<br>Sutradhar, R.<br>Stukel, T. A.	Gastroenterology	2008	Bleeding and perforation after outpatient colonoscopy and their risk factors in usual clinical practice	Mixed population - Data not stratified
Rabeneck, L.<br>Saskin, R.<br>Paszat, L. F.	Gastrointest Endosc	2011	Onset and clinical course of bleeding and perforation after outpatient colonoscopy: a population-based study	Mixed population - Data not stratified
Radaelli, F.<br>Meucci, G.<br>Minoli, G.	Dig Liver Dis	2008	Colonoscopy practice in Italy: a prospective survey on behalf of the Italian Association of Hospital Gastroenterologists	Mixed population - Data not stratified
Rathgaber, S. W.<br>Wick, T. M.	Gastrointest Endosc	2006	Colonoscopy completion and complication rates in a community gastroenterology practice	Mixed population - Data not stratified
Rathore, F.<br>Sultan, N.<br>Byrne, D.	Ir Med J	2014	Tolerance of colonoscopy and questioning its utility in the elderly population	Mixed population - Data not stratified
Reed, D. N., Jr.<br>Collins, J. D.<br>Wyatt, W. J.<br>Hull, J. E.<br>Patton, M. L.<br>Dahm, S. O.<br>Dabideen, H. H.<br>Hudson, J. C.<br>Allen, D. B.	Am J Surg	1992	Can general surgeons perform colonoscopy safely?	Not fitting defined screening population
Riegert, Monica<br>Nandwani, Monica<br>Thul, Bonny<br>Chiu, Angela Chang<br>Khashab, Mouen A.<br>Kalloo, Anthony N.	Gastrointestinal Endoscopy	2018	Experience of nurse practitioners performing colonoscopy after endoscopic training in over 1,000 patients.	General practice/Specialized endoscopic treatment center/other
Ringel, Y<br>Dalton, C B<br>Brandt, L J<br>Hu, Y<br>Jia, H	Gastrointest Endosc	2002	Flexible sigmoidoscopy: the patients' perception	Mixed population - Data not stratified

Bangdiwala, S<br>Drossman, D A				
Ristikankare, M.<br>Hartikainen, J.<br>Heikkinen, M.<br>Janatuinen, E.<br>Julkunen, R.	J Clin Gastroenterol	2001	The effects of gender and age on the colonoscopic examination	Not fitting defined screening population
Robinson, J. C.<br>Brown, T. T.<br>Whaley, C.<br>Finlayson, E.	JAMA Intern Med	2015	Association of Reference Payment for Colonoscopy With Consumer Choices, Insurer Spending, and Procedural Complications	Mixed population - Data not stratified.
Robinson, R. J.<br>Stone, M.<br>Mayberry, J. F.	Eur J Gastroenterol Hepatol	1996	Sigmoidoscopy and rectal biopsy: a survey of current UK practice	Not fitting defined screening population
S. Junna, T. R.<br>Golden, S.<br>Ghazala, R.<br>Wadeea, M.<br>Gonzalez, C. V. G.<br>Tirambulo, et al.	Gastroenterology	2018	FRAILTY PREDICTS COLONOSCOPY OUTCOMES IN PATIENTS UNDERGOING SCREENING	Conference abstract of another study included
Sabbagh, S<br>Romdhane, H<br>Ennaifer, R<br>Hefaiedh, R<br>Ben Nejma, H<br>Belhadj, N	European Surgery - Acta Chirurgica Austriaca	2013	Characteristics of colorectal cancer in a digestive endoscopy unit experience of mongi slim university hospital Tunesia	No harms investigated
Sadiq, Omar<br>Alimirah, Maryam<br>Askar, Firas<br>Siddiqui, Yousuf<br>Jinjuvadia, Raxitkumar<br>Naffouj, Sandra	American Journal of Gastroenterology	2019	Increased Frequency of Colonoscopy Complications in Patients With Positive Multitarget Stool DNA Testing for Colorectal Cancer Screening	No relevant intervention/work-up interventions/unconventional screening intervention
Sagawa, T.<br>Kakizaki, S.<br>Iizuka, H.<br>Onozato, Y.<br>Sohara, N.<br>Okamura, S.<br>Mori, M.	World J Gastroenterol	2012	Analysis of colonoscopic perforations at a local clinic and a tertiary hospital	Mixed population - Data not stratified.
Salmon, P<br>Shah, R<br>Berg, S<br>Williams, C	Endoscopy	1994	Evaluating customer satisfaction with colonoscopy	Not fitting defined screening population
Samalavicius, N. E.<br>Kazanavicius, D.<br>Lunevicius, R.<br>Poskus, T.<br>Valantinas, J.<br>Stanaitis, J.<br>Grigaliunas, A.<br>Gradauskas, A.<br>Venskutonis, D.<br>Samuolis, R.	Surg Endosc	2013	Incidence, risk, management, and outcomes of iatrogenic full-thickness large bowel injury associated with 56,882 colonoscopies in 14 Lithuanian hospitals	Not fitting defined screening population

Sniuolis, P.<br>Gajauskas, M.<br>Kaselis, N.<br>Leipus, R.<br>Radziunas, G.				
Sander, R.<br>Posl, H.<br>Weber, W.<br>Spuhler, A.	Leber Magen Darm	1979	[Endoscopic polypectomy in the colon, a calculated risk (author's transl)]	Not fitting defined screening population
Sarkar, S.<br>Geraghty, J.<br>Moore, A. R.<br>Lal, S.<br>Ramesh, J.<br>Bodger, K.	Eur J Gastroenterol Hepatol	2012	A multicentre study to determine the incidence, demographics, aetiology and outcomes of 6-day emergency readmission following day-case endoscopy	Not fitting defined screening population
Sasha Taylor, Farhad Salimi, Arul Earnest, Alexander G Heriot, John R Zalcberg, Susannah Ahern	MJA	2021	The short to medium term benefits of the Australian colorectal cancer screening program	No relevant intervention
Schmilovitz-Weiss, H.<br>Weiss, A.<br>Boaz, M.<br>Levin, I.<br>Chervinski, A.<br>Shemesh, E.	J Clin Gastroenterol	2007	Predictors of failed colonoscopy in nonagenarians: a single-center experience	Mixed population - Data not stratified.
Segnan, N.<br>Senore, C.<br>Andreoni, B.<br>Aste, H.<br>Bonelli, L.<br>Crosta, C.<br>Ferraris, R.<br>Gasperoni, S.<br>Penna, A.<br>Risio, M.<br>Rossini, F. P.<br>Sciallero, S.<br>Zappa, M.<br>Atkin, W. S.	J Natl Cancer Inst	2002	Baseline findings of the Italian multicenter randomized controlled trial of "once-only sigmoidoscopy"--SCORE	Mixed population - Data not stratified
Shah, S. G.<br>Brooker, J. C.<br>Thapar, C.<br>Williams, C. B.<br>Saunders, B. P.	Endoscopy	2002	Patient pain during colonoscopy: an analysis using real-time magnetic endoscope imaging	Mixed population - Data not stratified.
Shi, X.<br>Shan, Y.<br>Yu, E.<br>Fu, C.<br>Meng, R.<br>Zhang, W.<br>Wang, H.	Surg Endosc	2014	Lower rate of colonoscopic perforation: 110,785 patients of colonoscopy performed by colorectal surgeons in a large teaching hospital in China	Mixed population - Data not stratified.

Liu, L.<br>Hao, L.<br>Wang, H.<br>Lin, M.<br>Xu, H.<br>Xu, X.<br>Gong, H.<br>Lou, Z.<br>He, H.<br>Xing, J.<br>Gao, X.<br>Cai, B.				
Sieg, A.<br>Hachmoeller-Eisenbach, U.<br>Eisenbach, T.	Gastrointest Endosc	2001	Prospective evaluation of complications in outpatient GI endoscopy: a survey among German gastroenterologists	Not fitting defined screening population
Sieg, A.<br>Fur Die, B. N. G. Studiengruppe	Z Gastroenterol	2003	[Screening colonoscopy among persons 50 to 60 years of age with and without familial risk of colorectal cancer - a prospective multicenter trial]	Not fitting defined screening population
Singal, Amit G.<br>Gupta, Samir<br>Skinner, Celette Sugg<br>Ahn, Chul<br>Santini, Noel O.<br>Agrawal, Deepak<br>Mayorga, Christian A.<br>Murphy, Caitlin<br>Tiro, Jasmin A.<br>McCallister, Katharine<br>Sanders, Joanne M.<br>Bishop, Wendy Pechero<br>Loewen, Adam C.<br>Halm, Ethan A.	JAMA	2017	Effect of Colonoscopy Outreach vs Fecal Immunochemical Test Outreach on Colorectal Cancer Screening Completion: A Randomized Clinical Trial	Harm in view of health personnel
Singh, H.<br>Penfold, R. B.<br>DeCoster, C.<br>Kaita, L.<br>Proulx, C.<br>Taylor, G.<br>Bernstein, C. N.<br>Moffatt, M.	Gastrointest Endosc	2009	Colonoscopy and its complications across a Canadian regional health authority	Mixed population - Data not stratified.
Smith, L. E.	Dis Colon Rectum	1976	Fiberoptic colonoscopy: complications of colonoscopy and polypectomy	Not fitting defined screening population

Soo-Kyung Park, Min-Gu Lee, Seok Hyeon Jeong, Hyo-Joon Yang, Yoon Suk Jung, Kyu Yong Choi, Hungdai Kim, Hyung Ook Kim, Kyung Uk Jeong, Ho-Kyung Chun, Dong Il Park	Springer	2017	Prospective Analysis of Minor Adverse Events After Colon Polypectomy	Not fitting defined screening population
Soo-Kyung Park, Jeong Yeon Seo, Min-Gu Lee, Hyo-Joon Yang, Yoon Suk Jung, Kyu Yong Choi, Hungdai Kim, Hyung Ook Kim, Kyung Uk Jung, Ho-Kyung Chun, Dong Il Park	Springer	2018	Prospective analysis of delayed colorectal post-polypectomy bleeding	Not fitting defined screening population
Speroni, K. G. Hannah, J. Atherton, M. Corriher, J.	Gastroenterol Nurs	2005	Evaluation of demographic, behavioral, and procedural factors on pain perception by patients undergoing colonoscopy and moderate sedation	Not fitting defined screening population
Steffenssen, M. W. Al-Najami, I. Zimmermann-Nielsen, E. Baatrup, G.	Acta Oncol	2019	Patient-reported complications related to colonoscopy: a prospective feasibility study of an email-based survey	Not fitting defined screening population
Steine, S.	Radiology	1994	Which hurts the most? A comparison of pain rating during double-contrast barium enema examination and colonoscopy	Mixed population - Data not stratified.
Stuart, M. Failes, D. Killingback, M. De Luca, C.	Medical Journal of Australia	1979	Fibreoptic colonoscopy. Indications, results and complications	Not fitting defined screening population
Su, M Y Hsu, C M Lin, C J Ho, Y P Chiu, C T Chen, P C Lien, J M Wu, C S Tung, S Y	Dig Dis Sci	2008	Endoscopic treatment of colorectal neoplasms: a simple and safe procedure to lower the incidence of colorectal cancers	Not fitting defined screening population
Suchanek, S Majek, O Zavoral, M Seifert, B	Klin Onkol	2014	[Results of the Czech National Colorectal Cancer Screening Programme -	No harms investigated

Ngo, O<br>Dusek, L			colonoscopy examinations]	
Suissa, A<br>Bentur, O S<br>Lachter, J<br>Yassin, K<br>Chermesh, I<br>Gralnek, I<br>Karban, A<br>Khamaysi, I<br>Naveh, Y<br>Tamir, A<br>Shahbari, A<br>Eliakim, R	Diagnostic and Therapeutic Endoscopy	2012	Outcome and complications of colonoscopy: A prospective multicenter study in Northern Israel	Not fitting defined screening population
Swan, J. S.<br>Kong, C. Y.<br>Hur, C.<br>Halpern, E. F.<br>Itauma, O.<br>Williams, O.<br>Motazedi, T.<br>Lee, J. M.	J Am Coll Radiol	2015	Comparing morbidities of testing with a new index: screening colonoscopy versus core-needle breast biopsy	No original data
T. Decassian and M. Dauer	Z Gastroenterol	2019	[APP (Amberg-perforation-project) - development and evaluation of an interdisciplinary, systematic approach for endoscopic management of iatrogenic perforation in a German secondary referral center]	Unable to contact authors to clarify whether the population is a screening population
T. Mullaney and T. Eglinton	New Zealand Medical Journal	2019	The cost of colorectal complications in new zealand	No original data
Takahashi, Y.<br>Tanaka, H.<br>Kinjo, M.<br>Sakumoto, K.	Dis Colon Rectum	2005	Sedation-free colonoscopy	Mixed population - Data not stratified.
Takahashi, Y.<br>Tanaka, H.<br>Kinjo, M.<br>Sakumoto, K.	Dis Colon Rectum	2005	Prospective evaluation of factors predicting difficulty and pain during sedation-free colonoscopy	Mixed population - Data not stratified.
Tam, M. S.<br>Abbas, M. A.	Perm J	2013	Perforation following colorectal endoscopy: what happens beyond the endoscopy suite?	Mixed population - Data not stratified.
Tanaka, M.<br>Kawamura, M.<br>Tani, M.	Nihon Ronen Igakkai Zasshi	2000	[Comparison of complications in colonoscopic treatment in elderly and young subjects]	Mixed population - Data not stratified.
Tanigawa, K.<br>Yamashita, S.<br>Maeda, Y.	Chinese Medical Journal	1995	Endoscopic polypectomy for pacemaker patients	Not fitting defined screening population

Morita, S.<br>Tezuka, H.<br>Ohtsubo, T.<br>Nagataki, S.<br>Maeda, R.				
Taylor, K. M.<br>Arajs, K.<br>Rouse, T.<br>Harris, A. W.	Endoscopy	2008	Prospective audit of colonoscopy quality in Kent and Medway, UK	Mixed population - Data not stratified.
Teshima, C. W.<br>Zepeda-Gomez, S.<br>AlShankiti, S. H.<br>Sandha, G. S.	World J Gastroenterol	2014	Magnetic imaging-assisted colonoscopy vs conventional colonoscopy: a randomized controlled trial	Mixed population - Data not stratified.
Thiis-Evensen, E<br>Hoff, Gs<br>Sauar, J<br>Vatn, Mh	Gastrointest Endosc	2000	Patient tolerance of colonoscopy without sedation during screening examination for colorectal polyps	Unclear which interventions people receive
Thompson, A. M.<br>Park, K. G.<br>Kerr, F.<br>Munro, A.	British Journal of Surgery	1992	Safety of fibreoptic endoscopy: analysis of cardiorespiratory events	Mixed population - Data not stratified.
Timothy, S. K.<br>Hicks, T. C.<br>Opelka, F. G.<br>Timmcke, A. E.<br>Beck, D. E.	Dis Colon Rectum	2001	Colonoscopy in the patient requiring anticoagulation	Not fitting defined screening population
Torbjörn Thulin ,<br>Ulf Hammar,<br>Anders Ekbom,<br>Rolf Hultcrantz and<br>Anna M Forsberg	United European Gastroenterology Journal	2018	Perforations and bleeding in a population-based cohort of all registered colonoscopies in Sweden from 2001 to 2013	Not fitting defined screening population/Mixed population
Tran, D. Q.<br>Rosen, L.<br>Kim, R.<br>Riether, R. D.<br>Stasik, J. J.<br>Khubchandani, I. T.	The American Surgeon	2001	Actual colonoscopy: what are the risks of perforation?	Not fitting defined screening population
Traul, D. G.<br>Davis, C. B.<br>Pollock, J. C.<br>Scudamore, H. H.	Dis Colon Rectum	1983	Flexible fiberoptic sigmoidoscopy--the Monroe Clinic experience. A prospective study of 5000 examinations	Not fitting defined screening population
Tulchinsky, H.<br>Madhala-Givon, O.<br>Wasserberg, N.<br>Lelcuk, S.<br>Niv, Y.	World J Gastroenterol	2006	Incidence and management of colonoscopic perforations: 8 years' experience	Not fitting defined screening population
Ure, T.<br>Dehghan, K.<br>Vernava, A. M., 3rd<br>Longo, W. E.	Surg Endosc	1995	Colonoscopy in the elderly. Low risk, high yield	Mixed population - Data not stratified

Andrus, C. A.<br>Daniel, G. L.				
Ussui, V. M.<br>Silva, A. L.<br>Borges, L. V.<br>Silva, J. G.<br>Zeitune, J. M.<br>Hashimoto, C. L.	Arq gastroenterology	2013	What are the most important factors regarding acceptance to the colonoscopy?: study of related tolerance parameters	Mixed population - Data not stratified.
Viiala, C. H.<br>Zimmerman, M.<br>Cullen, D. J.<br>Hoffman, N. E.	Intern Med J	2003	Complication rates of colonoscopy in an Australian teaching hospital environment	Not fitting defined screening population
Villa, E.<br>Pasquinelli, C.<br>Rigo, G.<br>Ferrari, A.<br>Perini, M.<br>Ferretti, I.<br>Gandolfo, M.<br>Rubbiani, L.<br>Antonioli, A.<br>Barchi, T.<br>et al.,	Gastrointest Endosc	1984	Gastrointestinal endoscopy and HBV infection: no evidence for a causal relationship. A prospective controlled study	Not fitting defined screening population
Von Wagner, C.<br>Knight, K.<br>Halligan, S.<br>Atkin, W.<br>Lilford, R.<br>Morton, D.<br>Wardle, J.	Br J Radiol	2009	Patient experiences of colonoscopy, barium enema and CT colonography: a qualitative study	Not fitting defined screening population
Warren, J. L.<br>Klabunde, C. N.<br>Mariotto, A. B.<br>Meekins, A.<br>Topor, M.<br>Brown, M. L.<br>Ransohoff, D. F.	Ann Intern Med	2009	Adverse events after outpatient colonoscopy in the Medicare population	Mixed population - Data not stratified.
Waye, J. D.<br>Lewis, B. S.<br>Yessayan, S.	J Clin Gastroenterol	1992	Colonoscopy: a prospective report of complications	Not fitting defined screening population
Wexner, S. D.<br>Garbus, J. E.<br>Singh, J. J.	Surg Endosc	2001	A prospective analysis of 13,580 colonoscopies. Reevaluation of credentialing guidelines	Not fitting defined screening population
Winawer, Sj J<br>Miller, C<br>Lightdale, C<br>Herbert, E<br>Ephram, Rc C<br>Gordon, L<br>Miller, D	Cancer	1987	Patient response to sigmoidoscopy. A randomized, controlled trial of rigid and flexible sigmoidoscopy	Not fitting defined screening population
Wu, K.<br>Titzer, D.<br>Soetikno, R.	Gastrointest Endosc	2003	Use of a colonoscope instead of a sigmoidoscope to screen asymptomatic adults for colorectal cancer	Mixed population - Data not stratified.

Triadafilopoulos, G.				
Xirasagar, S<br>Hurley, T G<br>Sros, L<br>Hebert, J R<br>Xirasagar, Sudha<br>Hurley, Thomas G<br>Sros, Lekhena<br>Hebert, James R	Med Care	2010	Quality and safety of screening colonoscopies performed by primary care physicians with standby specialist support	General Practice
Y. C. Lee, T. H. Chiang, H. M. Chiu, M. S. Wu, Y. P. Yeh and T. Hsiu-Hsi Chen	Gastroenterology	2021	Community-Based Gastric Cancer Screening Coupled With a National Colorectal Cancer Screening Program: baseline Results	No harms investigated/Expected harm, not experienced/Harm in view of health personnel
Ylinen, E. R.<br>Vehvilainen-Julkunen, K.<br>Pietila, A. M.	Gastroenterol Nurs	2011	The Colorado Behavioral Numerical Pain Scale in assessing medication-free colonoscopy patients' pain	Not fitting defined screening population
Ylinen, E. R.<br>Vehvilainen-Julkunen, K.<br>Pietila, A. M.<br>Hannila, M. L.<br>Heikkinen, M.	J Adv Nurs	2009	Medication-free colonoscopy--factors related to pain and its assessment	Not fitting defined screening population
Ylinen, Eeva-Riitta R<br>Vehviläinen-Julkunen, Katri<br>Pietilä, Anna-Maija<br>Vehvilainen-Julkunen, K<br>Pietila, A M<br>Vehviläinen-Julkunen, Katri<br>Pietilä, Anna-Maija	J Clin Nurs	2009	Effects of patients' anxiety, previous pain experience and non-drug interventions on the pain experience during colonoscopy	Not fitting defined screening population
Yoshida, N.<br>Mano, Y.<br>Matsuda, T.<br>Sano, Y.<br>Inoue, K.<br>Hirose, R.<br>Dohi, O.<br>Itoh, Y.<br>Goto, A.<br>Sobue, T.<br>Takeuchi, Y.<br>Nakayama, T.<br>Muto, M.<br>Ishikawa, H.	J Gastroenterol Hepatol	2021	Complications of colonoscopy in Japan: An analysis using large-scale health insurance claims data	Not fitting defined screening population/ mixed population
Zubarik, R.<br>Fleischer, D. E.<br>Mastropietro, C.<br>Lopez, J.<br>Carroll, J.	Gastrointest Endosc	1999	Prospective analysis of complications 30 days after outpatient colonoscopy	Not fitting defined screening population

Benjamin, S.<br>Eisen, G.				

5 Appendix 5 - Data extraction templates

Template 1 – Study characteristics

- Study Number (assigned by reviewer)
- Study ID (First author - year)
- Referring to study protocol?
    - YES/NO
- Important deviations from protocol?
    - Free text
- Study limitations concerning harms noted by study authors
    - Free text
- Conclusions concerning harms noted by study authors
    - Free text
- Authors
- Title of publication
- Year of publication
- Funding
    - NR/Industry/Non-Industry/Mixed/Unclear
- Funding - details
    - Free text
- Conflicts of interest
    - Yes/No/Not Reported
- Conflicts of interest - details
    - Free text
- Study design
    - RCTs/Non-Randomized Study
- Study period
    - dd-mm-year - dd-mm-year
- Country/Countries
    - Free text
- Setting - Other

- Free text
- Population
  - Screening/Mixed
- Exclusion criteria
  - Reported/Not Reported/Noted no exclusion criteria
- Population details
  - Free text
- People invited, N
- People attending, N
- People performing FOBT, N
- FOBT+, N
- People receiving procedure, N
- Procedures performed, N
- Age interval
  - year-year
- Sex distribution
  - % women
- Mean age
- Median age
- Standard deviation
- Sociodemography
  - Reported/Not Reported
- Sociodemography - details
  - Free text
- CRCS methods applied
- FIT/FOBT test
  - Reported/Not Reported/ Not Performed
  - Type, dose, timing
- Bowel prep/Enema
  - Reported/Not Reported/ Not Performed
  - Type, dose, timing
- Anaestehesia (All types of medications before, during and after)

- Reported/Not Reported/ Not Performed
- Type, dose, timing

- Procedure
  - Sigmoidoscopy/Colonoscopy/Sigmoidoscopy to colonoscopy/Both/Control group
  - Expertise
- Polypectomy
  - Reported/Not Reported/ Not Performed
  - Details
- Overall definition of the harm domain physical harm
  - Not Reported/Definition

Template 2 – Outcome data
For each unique population of people reviewers noted if the following outcomes were assessed:

- Death
- Perforation
- Bleeding
- Pain
- Discomfort
- Cardiopulmonary
- Other

Any outcome that did not fit into the first six categories were categorized as other. When outcomes were reported as composite outcomes like "complications", serious adverse events etc. these were categorized as other.

<u>The following characteristics were noted by reviewers for all assessed outcomes:</u>

- Definition
  - Free text including any information supplied regarding thresholds, causality limits, severity limits etc.
- Time point(s) of assessment, i.e. when was the outcome assessed
  - Not Reported when outcome was assessed
  - Single time point
  - Composite > 1 time point of assessment, reporting of data summed up

    - Multiple > 1 time point of assessment, data reported per time point
- Follow-up
    - Longest duration of follow up in the event of multiple time points
    - Free text for details
- Outcome assessor
    - Person Performing Procedure, Self Report, Nurse, Research Assistant, Other, Not Reported
    - Free text for details
- Measurement tool
    - Not Reported, Interview, Questionnaire, Register, Other
    - Free text for details
- Analysis
    - Any changes between outcomes assessed and reported
    - Imputations for missing data, simplifications by merging categories or summing up scores, translations or interpretations from narrative material to numbers
- Number of people analyzed
- Number of procedures analyzed
- Effect size
- Consequences of harms
    - Reported/Not Reported
    - Details about consequences of harms (if reported)
- Modifiers assessed
    - Any covariate assessed for modifying the risk of the outcome
- Modifiers with significant association to outcome
    - Any covariate associated with the outcome, having a P-value < 0,05
- Size of effect
- Any effetcs size estimate like OR, RR etc.

6 Appendix 6 – The GRADE approach

In case of downgrading due to other factors than those described below, these are elaborated via footnotes as recommended in the GRADE Handbook (4).

Design

All studies included for the collective outcomes analyses, i.e., excluding the studies presented under *Results: Special cases*, were effectively one-armed studies. In view of this, and the fact that NRSs are likely equal if not better than RCTs to assess the harms of interventions, we judged those results both from NRSs and from RCTs were of low quality as a starting point for assessments. Below, we outline how we assessed the five factors that determines downgrading of the evidence and the three factors used for upgrading the evidence.

Downgrading factors

Risk of bias (1)

Studies with critical risk of bias were not part of the studies assessed via the GRADE approach.
We downgraded the evidence using the following cut-off values for the collective risk of bias across subpopulations that contributed with data for the outcome:

- If there was serious risk of bias in one or more bias domains <25% of study participants
    - 0
- If there was serious risk of bias in one or more bias domains 25-50 % of study participants
    - -1
- If there was serious risk of bias in one or more bias domains >50 % of study participants
    - -2

We used the weighted bias distributions tables for this assessment, thereby attributing more weight to larger studies.

Inconsistency of results (2)

In case of inconsistent results across subpopulations, we looked for plausible explanations using data about screening procedure delivery, study population characteristics and outcome measurement. In case we could not account for inconsistencies, we downgraded the evidence 1 or 2 levels, depending on the size of the inconsistencies. Below, we present the variables used to find plausible explanations for observed inconsistency in results, divided according to the PICOT format.

Population

Country and setting

Sex distribution

Age interval, mean age, median age, sociodemographic information.

Intervention

Expertise of endoscopists

With/without provision of anaesthesia and any details provided about anaesthetics used

Whether polypectomies were done and if so at which rate.

Comparator

Not applicable

Outcome measures

Any differences in outcome definition, measurement method, who measured the outcome and the follow-up time included.

Indirectness of evidence (3)

The eligibility criteria for the review defines the population of interest, the target population. To assess indirectness of evidence, we compared the review's eligibility criteria to characteristics of screening procedure delivery, study population and outcome measurement in included studies. We only accepted small deviations between eligibility criteria and characteristic of included studies; hence, we expected the evidence to be very direct in terms of answering the research question. **Therefore, this GRADE domain was not meaningful to assess**. Eligibility criteria for the review are available in appendix 2. Below, we outline our considerations about the core components of eligible studies that determine their directness/indirectness related to the research question of interest, in the PICOT format.

Population

We had strict criteria for the population of interest. We excluded any study outside of these criteria. We accepted minor deviations, e.g., a small percentage of the study population being above or below the age interval or in case a small part of the study population that was not in mean risk of CRC due to symptoms of CRC or due to any other reason for increased risk of CRC. In case a study population received one or more different screening procedure, e.g., some receiving flexible

sigmoidoscopy and other receiving FOBT and colonoscopy, we divided the study population according of the 4 screening procedure groups. As a result, we view all included studies as direct evidence concerning the interventions provided.

Intervention

We exclusively included studies that provided any combination of FOBT, colonoscopy and sigmoidoscopy and no other co-interventions other than those normally associated with these interventions, e.g., bowel preparation, anaesthesia and polypectomy. Following data extraction, we divided all studies according to the 4 types of screening procedures to facilitate homogenous groups. As a result, we view all included studies as direct evidence concerning the interventions provided.

Comparator

Not applicable

Outcome measures

We only included outcomes that were measured systematically. We extracted data concerning how the outcome was defined, how it was measured, by whom and the follow-up time. Following data extraction, we subcategorized the outcomes to create more homogenous outcome groups. As a result, we view all included studies as direct evidence concerning the outcomes analyzed.

Time

We did not downgrade the evidence due to the year the studies were conducted, e.g., studies conducted in the 90'ies versus the 2010's, as we expected the rate of adverse events to be constant over time. This is in line with a recent systematic review that found a small decline in the risk of post-colonoscopy bleeding and a stable rate of perforation and mortality due to screening of studies conducted between 2001 to 2015 (5).

Imprecise results (4)

We used the optimal information size (OIS) to assess whether the evidence should be downgraded.

We established the OIS as the number of people needed for 1 event to occur of a given outcome. To establish this estimate, we calculated the weighted mean risk of the outcome across those identified systematic reviews that quantified the outcome with the potential case risk estimate from this review added to the analysis. We added the potential case risk estimate from this review due to the hypothesis that physical harms have been underestimated in former reviews. Because of adding our estimate to the OIS calculation, the OIS becomes smaller, leading to a less strict downgrade criterion than if our results had not been added to the analysis.

We calculated OIS for each subgroup of bleeding and perforation as the inverse of the weighted mean for each subgroup of bleeding and perforation with data collected for the potential case analysis.

Calculations of OIS for bleeding and perforation are available below.

We downgraded the evidence with the following degrees using the criteria outlined below:

1. Do not rate down
   a. < 50 % of studies have a size below the OIS and/or
   b. The 95% confidence interval for the collective outcomes excludes no effect
2. Rate down 1 level
   a. >50 % of studies have a sample size below the OIS and/or
   b. The 95% confidence interval for the collective outcomes overlaps no effect
3. Rate down 2 levels
   a. Very few events, in general < 5 AND
   b. The 95% confidence interval for the collective outcomes overlaps no effect

7 Appendix 7 – Subcategories of bleeding

Appendix 7: Subcategories of bleeding.

Definitions of bleeding events	Follow-up time	Occurrences	Analysis category
Bleeding starting within 1 month and lasting for longer than 4 days	30 days		Mild-longterm
Bleeding that occurred and was managed during the procedure was considered as an incident	30 days		Mild-longterm

lower GL bleeding mild	30 days		Mild-longterm
Minimal rectal bleeding	30 days		Mild-longterm
Minor and intermediate	30 days		Mild-longterm
Minor GI bleeding	30 days		Mild-longterm
non serious bleeding per rectum reported when deemed to be at least possibly resulting from the colonoscopy	30-45 days		Mild-longterm
not requiring transfusion - We also identified all adverse events occurring within 30 days after baseline that were severe enough to require an emergency department visit or hospitalization. We classified adverse events as serious gastrointestinal (perforation, gastrointestinal bleeding requiring transfusion), other gastrointestinal (gastrointestinal bleeding not requiring transfusion, paralytic ileus, nausea, vomiting and dehydration, abdominal pain), or cardiovascular (myocardial infarction or angina, arrhythmias, congestive heart failure, cardiac or respiratory arrest, syncope, hypotension or shock) events	30 days	2	Mild-longterm
Periprocedural bleeding which is controlled during the procedure and which does not prevent completion of the procedure is not considered to be an adverse event.<br><br>Bleeding was analyzed for all grades of severity and subanalyzed for bleeding requiring transfusion	30 days		Mild-longterm
self-limited lower GI bleeding	28 days		Mild-longterm
bleeding from buttom after going home - An adverse event is defined by the NHSBCSP as an event that prevents the completion of the procedure (excluding technical failure or poor bowel preparation) and/or results in: (i) admission to hospital or prolonged hospital stay; (ii) another intervention (endoscopic, radiological, or surgical); or (iii) subsequent medical consultation.	Not Reported		Mild-NR
Bleeding starting within 1 month and lasting for longer than 4 days	Not Reported		Mild-NR
less complications: bleeding controlled at endoscopy (no transfusion or hospitalization)	Not Reported		Mild-NR
minimal bleeding with hospitalization	Not Reported		Mild-NR
Self limiting	Not Reported	4	Mild-NR
no clinically significant complications including i.e., perforation, need for hospitalization, or clinically important bleeding	Not Reported		Mild-NR
Bleeding requried no intervention or tranfusion	Not Reported		Mild-NR
bleeding events of none required transfusion or surgery.	Not Reported		Mild-NR
managed by epinephrine injection, heat coagulation or hemoclip at the time of occurrence. No blood transfusion or hospital admission was needed.	Not Reported		Mild-NR
bleeding requiring hospitalization and transfusion	30 days		Severe-longterm
bleeding requiring hospitalization	30 days		Severe-longterm

lower GL bleeding moderate	30 days		Severe-longterm
post-colonoscopy bleeding that required treatment or prompted the patient to contact a hospital for medical evaluation.	14 days		Severe-longterm
Serious bleedings categorised as adverse events	30 days		Severe-longterm
"post-polypectomy bleeding leading to hospitalization<br><br>diagnosis codes for hemorrhage and<br>hematoma complicating a procedure, hemorrhage of anus and<br>rectum, posthemorrhagic anemia, melena, gastrointestinal hemorrhage,<br>unintentional cut, puncture, perforation, or hemorrhage<br>during surgical and medical care, during endoscopic examination<br>or other surgical and medical care"	14 days		Severe-longterm
A severe complication was considered as a late complication when it occurred any time from the following day to 30 days after the colonoscopy.<br><br>‘‘complication which requires hospital admission or causes death due to perforation or haemorrhage which requires transfusion or severe vasovagal syndrome or peritonitis, and occurs within a period of 0 to 30 days from completion of the colonoscopy’’.5<br>A severe complication was considered as an immedi- ate complication when it occurred on the same day as the colonoscopy and as a late complication when it occurred any time from the following day to 30 days after the colonoscopy.	30 day		Severe-longterm
Complications were considered major if they required a blood transfusion, hospitalization, surgery, or if they resulted in death. Data on all complications within 30 days were collected	30 days		Severe-longterm
hospitalization within 30 days for serious hemorrhage involving transfusion	10 days		Severe-longterm
Major	30 days		Severe-longterm
Major adverse events. Defined as requiring hospital admission within 30 days of a screening colonoscopy.	30 days		Severe-longterm
Major complications were defi ned as any event related to the procedure and requiring admission within 30 days of the procedure. This includes perforations, post polypectomy bleeding requiring transfusion and/or hospital admission, cardiopulmonary events, and neurologic events	30 days		Severe-longterm
major complications were defined as any conditions or symptoms that resulted in hospital admission within 30 days of the procedure and included perforation, gastrointestinal bleeding requiring or not requiring blood transfusion, cardiopulmonary events, postpolypectomy syndrome, excessive abdominal pain, and death. All complications occurring within 30 days of any program test were included, regardless of whether the medical records associated the complication with the procedure	30 days		Severe-longterm
Major post-polypectomy adverse events were defined as: bleeding episodes requiring transfusion	30 days		Severe-longterm
requiring hospitalization	30 days		Severe-longterm
requiring transfusion	30 days	2	Severe-longterm
Serious AEs of interest were bleeding	30 days		Severe-longterm

serious bleeding events	30 days	2	Severe-longterm
serious lower GL bleeding	30 days	3	Severe-longterm
serious: gastrointestinal bleeding that required hospitalization	30 days		Severe-longterm
severe complications	30 days		Severe-longterm
severe complications	30 days		Severe-longterm
significant bleeding within 14 days was defined as severe complications per 1000 procedures	14 days		Severe-longterm
We defined significant bleedings as bleedings that lead to hospitalization (!1 day), blood transfusion, repeat endoscopy, radiologic intervention, or surgery	30 days	3	Severe-longterm
We defined bleeding as blood per rectum<br><br>We defined unplanned events as serious adverse events if they resulted in death, hospital admission or important inter- vention, and we subclassified them (by consensus) as probably, possibly or unlikely related to the colonoscopy.	0-14 days		Severe-longterm
heavy bleeding from the intestine - severe bleeding 12 days after polypectomy	12 days		Severe-longterm
requiring transfusion and hospitalization	Not Reported		Severe-NR
Immediate or delayed postpolypectomy bleeding - requiring transfusion and hospitalization	Not Reported		Severe-NR
"admitted to the hospital for postpolypec-<br>tomy bleeding following snare polypectomy."	Not Reported	2	Severe-NR
(requiring operative vs. conservative treatment, the latter comprising endoscopic procedures - acute complications	Not Reported		Severe-NR
admitted to hospital for bleeding	Not Reported	2	Severe-NR
An adverse event is defined by the NHSBCSP as an event that prevents the completion of the procedure (excluding technical failure or poor bowel preparation) and/or results in: (i) admission to hospital or prolonged hospital stay; (ii) another intervention (endoscopic, radiological, or surgical); or (iii) subsequent medical consultation.	Not Reported		Severe-NR
Bleeding requirering readmission	Not Reported		Severe-NR
Bleeding requiring transfusion - severe complications during the diagnostic procedure	Not Reported		Severe-NR
Bleeding was defined as a condition requiring interventions (either by endoscopic techniques or surgery).	Not Reported		Severe-NR
immediate hospitalization	Not Reported	2	Severe-NR
Major bleeding	Not Reported	2	Severe-NR
Massive bleeding requirering transfusion	Not Reported		Severe-NR
Post polypectomy bleeding involving transfusion<br>or hospitalisation of at least 24 hours	Not Reported		Severe-NR

Post-colonoscopy bleeding requiring admission	Not Reported		Severe-NR
serious bleeding	Not Reported		Severe-NR
Severe arterial bleeding after polyp resection requiring transfusions and hospitalization	Not Reported		Severe-NR
Severe hemorrhage	Not Reported	2	Severe-NR
significant bleeding (requiring blood transfusion, hospitalization, or surgery)	Not Reported		Severe-NR
Rectal hemorrhage during or immediately after the procedure requiring hospitalization or blood transfusion	24 h		Severe-NR

8 Appendix 8 – Subcategories of perforation

Appendix 8: Subcategories of perforation.

Definitions of perforation	Follow-up	Occurrences	Analysis category
minimal adverse events	30 days		Mild-longterm
mild	30 days		Mild-longterm
moderate	30 days		Mild-longterm
Perforation was defined as radiologic (computer tomography) findings consistent with intestinal perforation.	30 days	3	Mild-longterm
Perforation requiring hospitalization	30 days		Severe-longterm
See appendix 1<br><br>An adverse event is defined by the NHSBCSP as an event that prevents the completion of the procedure (excluding technical failure or poor bowel preparation) and/or results in: (i) admission to hospital or prolonged hospital stay; (ii) another intervention (endoscopic, radiological, or surgical); or (iii) subsequent medical consultation.<br>Any perforation within 30 days of the procedure	30 days		Severe-longterm
serious adverse event	30 days		Severe-longterm
hospitalization within 30 days for perforation	10 days		Severe-longterm
colonoscopic perforation leading to hospitalization	7 days		Severe-longterm
major complications were defined as any conditions or symptoms that resulted in hospital admission within 30 days of the procedure and included perforation, gastrointestinal bleeding requiring or not requiring blood transfusion, cardiopulmonary events, postpolypectomy syndrome, excessive abdominal pain, and death. All complications occurring within 30 days of any program test were included, regardless of whether the medical records associated the complication with the procedure	30 days		Severe-longterm

Major post-polypectomy adverse events were defined as: colonic perforation within 30 days of the procedure	30 days		Severe-longterm
We also identified all adverse events occurring within 30 days after baseline that were severe enough to require an emergency department visit or hospitalization.	30 days	2	Severe-longterm
All adverse events deviating from a normal postoperative course were registered after scrutinizing the hospital charts. In patients with multiple complications, the most severe of these was registered according to the Clavien–Dindo classification of surgical complications.	30 days		Severe-longterm
Major complications were defi ned as any event related to the procedure and requiring admission within 30 days of the procedure.	30 days		Severe-longterm
serious complications	30 days		Severe-longterm
Major adverse events and mortality. Defined as requiring hospital admission within 30 days of a screening colonoscopy.	30 days		Severe-longterm
defined as the composite of unplanned hospital admissions or emergency department visits at 30 days after outpatient colonoscopy.	30 days		Severe-longterm
Severe complications within 14 days per 1000 procedures	14 days		Severe-longterm
A severe complication was considered as a late complication when it occurred any time from the following day to 30 days after the colonoscopy.	30 days		Severe-longterm
severe	30 days		Severe-longterm
severe complications	30 days	2	Severe-longterm
serious adverse event	30-45 days		Severe-longterm
Serious GL complication	30 days		Severe-longterm
All requiring surgery	Not Reported		Severe-NR
This patient required surgery	Not Reported		Severe-NR
requiring surgery	Not Reported		Severe-NR
An adverse event is defined by the NHSBCSP as an event that prevents the completion of the procedure (excluding technical failure or poor bowel preparation) and/or results in: (i) admission to hospital or prolonged hospital stay; (ii) another intervention (endoscopic, radiological, or surgical); or (iii) subsequent medical consultation.	Not Reported		Severe-NR
involving transfusion or hospitalisation of at least 24 hours	Not Reported		Severe-NR
Causing hospitalization - Immediate complications	Not Reported	2	Severe-NR
Perforation is a well-defined severe complication and almost constantly leads to hospital admission.	Not Reported		Severe-NR
requiring operative vs. conservative treatment, the latter comprising endoscopic procedures - acute complications	Not Reported		Severe-NR
severe complications during the diagnostic procedure	Not Reported		Severe-NR
The following complications were defined as major complications	Not Reported		Severe-NR

major perforation	Not Reported		Severe-NR
Colonic perforation during or immediately after the procedure requiring hospitalization or blood transfusion	24 h		Severe-NR
Acute complications	During	2	Severe-NR

9 Appendix 9 – Study characteristics of special case studies and studies with an unscreened control group

Appendix 9: Study characteristics of special case studies and studies with an unscreened control group.

Study	Study design	Procedure	People, N	Compared to other studies?	Control group?	Cause for separate analysis	Bleeding assessed	Perforation assessed
**Adler 2013**	NRS	Once-only colonoscopy	12134	NO	NO	Complex study design	YES	YES
**Ladabaum 2021**	NRS	Once-only colonoscopy	4482598	NO	NO	Complex study design	YES	YES
**Stock 2013**	NRS	Once-only colonoscopy	8658	YES	YES	Control group	YES	YES
**Stock 2013**	NRS	Control	8658	NO	YES	Control group	YES	YES
**Garcia-Albeniz 2017**	NRS	Once-only colonoscopy	46872	YES	YES	Control group	YES	YES
**Garcia-Albeniz 2017**	NRS	Control	1762816	NO	YES	Control group	YES	YES
**Garcia-Albeniz 2017**	NRS	Once-only colonoscopy	31193	YES	YES	Control group	YES	YES
**Garcia-Albeniz 2017**	NRS	Control	1628020	NO	YES	Control group	YES	YES

Studies that had incomparable study designs were analyzed separately. One NRS study by Adler et al. with 12,134 people screened with once-only colonoscopy was handled separately due to multiple outcome assessment methods of bleeding and perforation: 1) via two case report forms were filled out by the physicians, 2) via a questionnaire to people screened and 3) via register data from a screening database. Complication rates in the study data was 3.5 times higher for bleeding and 1.5

times higher for perforation in case report forms and questionnaire respectively compared to the registry data (2).

Three studies included an unscreened control group. All three studies were NRSs and provided once-only colonoscopy as the screening procedure. Stock et al. and García-Albéniz et al. had 30 days follow-up and Ladabaum et al. had 180 days follow-up
(3–5). In all studies, outcome was assessed using register data with unclear validity leading to serious risk of measurement and missing data bias. In García-Albéniz's study there was 14 times higher risk of perforation events in the intervention group compared to the control group (5). Stock reported 7 perforation events in the intervention group and 0 events in the control group with a sample size of 8658 people (4). In Ladabaum's study the observed rate of perforation was compared to an expected event rate for perforation, which showed that CRCS increased the background risk of getting a perforation by 3.75 times (3). Using data from Stock, we found a 4 times higher risk of bleeding events in the intervention group compared to the control group. In García-Albéniz's study there was 10 times higher risk of bleeding events in the intervention group compared to the control group. In Ladabaum's study the observed to expected event rate for bleeding showed that CRCS increased the background risk for bleeding events by 3.55 times.

Appendix 9: Calculated incidence rate ratios (IRR) for bleeding and perforation.

**Bleeding**			
**Study**	**Category**	**Procedure**	**IRR**
Stock 2013	Severe-longterm	Once-only colonoscopy	4
Garcia-Albeniz 2017	Mild-longterm	Once-only colonoscopy	6
Garcia-Albeniz 2017	Mild-longterm	Once-only colonoscopy	5
Garcia-Albeniz 2017	Severe-longterm	Once-only colonoscopy	10
Garcia-Albeniz 2017	Severe-longterm	Once-only colonoscopy	4
**Perforation**			
**Study**	**Category**	**Procedure**	**IRR**
Stock 2013	Severe-longterm	Once-only colonoscopy	NA
Garcia-Albeniz 2017	Severe-longterm	Once-only colonoscopy	14
Garcia-Albeniz 2017	Severe-longterm	Once-only colonoscopy	8

IRR: First, we calculated the incidence rate (IR) by dividing the number of events with the number of people at risk for the control group and the intervention group. Next, we calculated the ratio between the two incidence rates.

10 Appendix 10 – Characteristics of additional subpopulations

Appendix 10: Characteristics of subpopulations that are not included in the meta-analyses.

Outcome	Subcategory	Study	Design	People	Procedure	Tool	Event	Incidence Rate (E/P) per 100,000 people	Worst bias score
Perforation	Mild-longterm	Pedersen 2021	NRS	11163	Once-only colonoscopy	Register	148	1,000	Serious
	Mild-longterm	Randel 2021 (s23c)	RCT	3297	Colonoscopy following any screening tests	Register	23	700	Serious
Bleeding	Severe-longterm	Randel 2021 (s23a)	RCT	36065	Sigmoidoscopy	Register	3	8	Serious
	Mild-longterm	Hol 2010a (19b)	RCT	332	Colonoscopy following any screening tests	NR	4	1,000	Critical

We were not able to conduct meta-analyses for four subcategories as there was only one study in the respective subcategory (6–8). Two of the studies found that the risk of mild perforation events with long-term follow-up was 700-1000 per 100,000 people screened (7,8). The risk of severe bleeding events with long-term follow-up was 8 per 100,000 screened in one study (8), whereas the last study, having long-term follow-up, found that 1000 mild bleedings occur per 100,000 screened (6). All outcome assessments had either serious or critical risk of bias.

11 Appendix 11 – Conversion factor for each procedure group

Appendix 11: Conversion factors.

Sigmoidoscopy		Colonoscopy following FOBT/FIT		Colonoscopy following any screening tests		Once-only colonoscopy	
People	225563	People	676.802,30	People	24.561,00	People	788.341,00
Procedure	267597	Procedure	800.430,00	Procedure	25.185,00	Procedure	801.872,00
People/procedure	0,842920511	People/procedure	0,8455483881	People/procedure	0,9752233472	People/procedure	0,98
Procedures/people	1,186351485	Procedures/people	1,182664427	Procedures/people	1,025406132	Procedures/people	1,02

12 Appendix 12 – Combination of subcategories

Appendix 12: The number of subpopulations in the respective subcategory stratified on procedure group.

**Outcome**	**Procedure**	**Subcategories**	**Subpopulations**
Bleeding	Colonoscopy following any screening tests	Severe-longterm	3
		Severe-NR	5
		Mild-longterm	1
		Mild-NR	4
		ND-longterm	3
		ND-NR	2
	Sigmoidoscopy	Severe-longterm	1
		Severe-NR	4
		Mild-longterm	2
		Mild-NR	3
		ND-longterm	3
		ND-NR	6
	Once-only colonoscopy	Severe-longterm	9
		Severe-NR	7
		Mild-longterm	5
		Mild-NR	3
		ND-longterm	8
		ND-NR	9
	Colonoscopy following FOBT/FIT	Severe-longterm	15
		Severe-NR	8
		Mild-longterm	4
		Mild-NR	2
		ND-longterm	5
		ND-NR	13
Perforation	Colonoscopy following any screening tests	Severe-longterm	2
		Severe-NR	2
		Mild-longterm	1
		ND-longterm	3
		ND-NR	7
	Sigmoidoscopy	Severe-longterm	0
		Severe-NR	0
		Mild-longterm	2
		ND-longterm	2

		ND-NR	6
	Once-only colonoscopy	Severe-longterm	7
		Severe-NR	6
		Mild-longterm	2
		ND-longterm	7
		ND-NR	15
	Colonoscopy following FOBT/FIT	Severe-longterm	13
		Severe-NR	5
		Mild-longterm	2
		ND-longterm	6
		ND-NR	20

13 Appendix 13 – Characteristics of procedure groups

Appendix 13: Characteristics of procedure groups.

	**Once-only colonoscopy**	**Colonoscopy following FIT**	**Sigmoidoscopy**	**Colonoscopy following any screening tests**
No. Subpopulations: 151				
**Perforation**				
Subpopulations, procedure, N	48 (32%)	54 (36%)	29 (19%)	20 (13%)
*Procedure & outcome	34 (23%)	45 (30%)	11 (7%)	15 (10%)
People, N	4,881,126	1,324,252	293,901	67,191
Procedures, N	5,493,536	1,174.998	347,741	83,968
Procedures per person, N	1.1	0.8	1.1	1.2
Women%, weighted mean	54.31	43.15	49.40	54.72
Age range	30-99 (74%)	20-93 (89%)	50-79 (73%)	40-86 (67%)
Mean age, weighted mean	64 (65%)	64 (33%)	61 (27%)	60 (33%)
Studies with provision of polypectomies, N	23 (68%)	29 (64%)	9 (82%)	9 (60%)
Studies with known polypectomy rate, N	10 (29%)	10 (22%)	0 (0%)	2 (13%)
Polypectomy rate, weighted mean (%)	56.15	41.71	0	44.85
**Bleeding**				
Subpopulations, Procedure, N	48 (32%)	54 (36%)	29 (19%)	20 (13%)
*Procedure & outcome	32 (21%)	44 (29%)	13 (9%)	15 (10%)

People, N	4,354,332	1,267,808	228,178	48,909
Procedures, N	4,900,647	1,124,916	269,978	61,121
Procedures per person, N	1.1	0.8	1.1	1.2
Women%, weighted mean	54.32	43.41	49.41	54.27
Age range	30-99 (78%)	20-93 (91%)	50-79 (62%)	40-86 (67%)
Mean age, weighted mean	64 (66%)	63 (34%)	60 (23%)	60 (27%)
Studies with provision of polypectomies, N	22 (69%)	32 (73%)	13 (100%)	11 (73%)
Studies with known polypectomy rate, N	9 (28%)	11 (25%)	0 (0%)	2 (13%)
Polypectomy rate, weighted mean (%)	56.52	43.59	0	98.54

(%): the percentage of the subpopulations that report information about characteristics

*: All variables from the row "people, N" and down are for the subpopulations where the outcome is assessed - e.g., when there are 4,881,126 people it is calculated for the 34 subpopulations that have assessed perforation and the same applies to all rows further down. The same goes for bleeding.

14 Appendix 14 - Adequacy of harm measurement across studies for bleeding

Appendix 14: Appraisal of harm reporting of bleeding.

	No. Publications	Proportion, %
Subpopulations total: 123		
Follow-up time	67	54%
Outcome assessor	45	37%
Measurement tool	78	63%

15 Appendix 15 - Adequacy of harm measurement across studies for perforation

Appendix 15: Appraisal of harm reporting of perforation.

	No. Publications	Proportion, %
Subpopulations total: 108		
Follow-up time	54	50%
Outcome assessor	36	33%
Measurement tool	64	59%

16 Appendix 16 - Bias distributions across all studies that assess bleeding

Study ID	Design	Procedure	Critical	Worst score	Inception	Classification	Performance	Missing data	Measurement	Reporting
Atkin 2002	RCT	FS		Serious	?	U	U	?	U	?
Atkin 1998	RCT	FS	YES	Critical	?	?	?	U	?	?
Atkin 1998	RCT	FS	YES	Critical	?	?	?	U	?	?
Forbes 2006	RCT	FS		Serious	?	U	U	U	?	?
Pabby 2005	NRS	FS		Serious	U	U	U	U	U	?
Randel 2021	RCT	FS		Serious	?	U	?	?	U	?
Atkin 2002	RCT	FS		Serious	?	U	U	?	U	?
Levin 2002	NRS	FS		Serious	?	U	U	U	U	?
Holme 2014	RCT	FS	YES	Critical	?	U	U	U	U	U
Jain 2002	NRS	FS	YES	Critical	?	U	U	U	U	?
Kewenter 1996	RCT	FS	YES	Critical	?	U	U	?	U	?
Levin 2002	NRS	FS	YES	Critical	?	U	U	U	U	?
Segnan 2002	RCT	FS	YES	Critical	?	U	?	U	U	?
Segnan 2002	RCT	FS	YES	Critical	?	U	?	U	U	?
Segnan 2005	RCT	FS	YES	Critical	?	U	U	U	U	?
Segnan 2005	RCT	FS	YES	Critical	?	U	U	U	U	?
Senore 2011	RCT	FS	YES	Critical	?	?	U	U	U	?
Arana-Arri 2018	NRS	TCfobt	YES	Critical	?	?	?	U	U	?
Binefa 2013	NRS	TCfobt	YES	Critical	U	?	?	U	U	?
Cheng 2002	NRS	TCfobt	YES	Critical	?	?	?	?	U	?
Dancourt 2008	NRS	TCfobt	YES	Critical	?	?	?	U	U	?
Denis 2007	NRS	TCfobt	YES	Critical	?	?	?	U	U	?
Rutter 2014	NRS	TCfobt		Serious	U	U	U	U	U	?
Lee 2012	NRS	TCfobt		Serious	?	?	U	U	U	?
Denis 2021	NRS	TCfobt		Serious	?	U	?	U	U	?
Robertson 2019	RCT	TCfobt		Serious	?	U	?	?	?	?
Denters 2012	NRS	TCfobt	YES	Critical	U	?	?	?	U	?
Ghanouni 2016	NRS	TCfobt		Serious	U	?	?	U	?	?
Hughes 2005	NRS	TCfobt		Serious	U	?	?	U	U	?
Denis 2013	NRS	TCfobt		Moderate	?	?	?	?	?	?
Saraste 2016	NRS	TCfobt		Serious	?	U	?	U	U	?
Blanks 2015	NRS	TCfobt		Serious	U	O	?	U	U	?
Karlijn 2021	NRS	TCfobt		Serious	?	?	?	U	U	?
Denters 2013	NRS	TCfobt	YES	Critical	U	?	?	U	U	?
Din 2017	NRS	TCfobt	YES	Critical	?	?	?	U	U	?
Ellul 2010	NRS	TCfobt	YES	Critical	?	?	?	U	U	?
Faivre 2004	NRS	TCfobt	YES	Critical	?	?	?	U	U	?
Garcia 2012	NRS	TCfobt	YES	Critical	?	U	?	U	U	?
Sung 2003	NRS	TCfobt		Serious	U	?	?	U	U	?
Mikkelsen 2018	NRS	TCfobt		Serious	?	?	?	U	U	?
Lee 2012	NRS	TCfobt		Serious	?	?	U	U	U	?
Din 2015	NRS	TCfobt		Serious	U	?	?	U	U	?
Benazzato 2020	NRS	TCfobt		Moderate	?	?	?	?	?	?
Ibánez 2018	NRS	TCfobt		Serious	?	?	?	U	U	?
Paszat 2020	NRS	TCfobt		Serious	?	U	?	U	?	?
Portillo 2018	NRS	TCfobt		Serious	?	?	?	?	U	?
Tomaszewski 2021	NRS	TCfobt		Serious	?	U	?	U	U	?
Vanaclocha-Espi 2018	NRS	TCfobt		Serious	?	?	?	U	U	?
Denis 2021	NRS	TCfobt		Serious	?	U	?	U	U	?
Hsu 2020	NRS	TCfobt		Serious	?	?	?	U	U	?
Randel 2021	RCT	TCfobt		Serious	?	U	U	?	U	?
Robertson 2019	RCT	TCfobt		Serious	?	U	?	?	?	?
Dominitz 2019	RCT	TCfobt		Serious	?	?	?	?	?	?

Marino 2012	NRS	TCfobt	YES	Critical	?	?	?	U	U	?
Meulen 2021	NRS	TCfobt	YES	Critical	?	?	?	U	U	?
Gupta 2012	NRS	TCfobt		Serious	U	?	?	U	U	?
Meulen 2021	NRS	TCfobt	YES	Critical	?	?	?	U	U	?
Neely 2013	NRS	TCfobt	YES	Critical	?	?	?	U	U	?
Parente 2013	NRS	TCfobt	YES	Critical	?	?	?	U	U	?
Quintero 2012	RCT	TCfobt	YES	Critical	?	?	?	U	U	?
Quyn 2018	NRS	TCfobt	YES	Critical	?	?	?	?	U	?
Robinson 1999	RCT	TCfobt	YES	Critical	U	?	?	?	?	?
Tepes 2017	NRS	TCfobt	YES	Critical	?	?	?	?	U	?
Zorzi 2009	NRS	TCfobt	YES	Critical	U	U	U	U	U	U
Castro 2013	NRS	TCfollowup	YES	Critical	?	?	?	U	U	?
Gondal 2003	RCT	TCfollowup	YES	Critical	?	?	?	U	U	?
Hol 2010a	RCT	TCfollowup	YES	Critical	?	?	?	U	U	?
Holme 2014	RCT	TCfollowup	YES	Critical	?	?	?	U	U	U
Kewenter 1996	RCT	TCfollowup	YES	Critical	?	?	?	?	U	?
Mandel 1993	RCT	TCfollowup	YES	Critical	?	?	?	?	U	?
Naumann 2021	NRS	TCfollowup	YES	Critical	?	U	?	?	U	?
Atkin 2002	RCT	TCfollowup		Serious	?	U	?	?	U	?
Forbes 2006	RCT	TCfollowup		Serious	?	?	?	U	?	?
Rajendran 2017	NRS	TCfollowup	YES	Critical	?	?	?	U	U	?
Randel 2021	RCT	TCfollowup		Serious	?	U	?	?	U	?
Rutter 2012	NRS	TCfollowup	YES	Critical	?	?	?	U	U	?
Atkin 2002	RCT	TCfollowup		Serious	?	U	?	?	U	?
Segnan 2002	RCT	TCfollowup	YES	Critical	?	?	?	U	U	?
Segnan 2002	RCT	TCfollowup	YES	Critical	?	?	?	U	U	?
Segnan 2005	RCT	TCfollowup	YES	Critical	?	?	?	U	U	?
Segnan 2005	RCT	TCfollowup	YES	Critical	?	?	?	U	U	?
Shroff 2015	NRS	TCfollowup	YES	Critical	?	U	?	U	U	?
Berhane 2009	NRS	TConly	YES	Critical	O	?	?	U	U	?
Crispin 2009	NRS	TConly	YES	Critical	?	?	?	U	U	?
Dae 2007	NRS	TConly	YES	Critical	?	?	?	U	U	?
Huppe 2004	NRS	TConly	YES	Critical	?	?	?	U	U	?
Imperiale 2000	NRS	TConly	YES	Critical	?	?	?	?	U	?
Kozbial 2015	NRS	TConly	YES	Critical	U	?	?	U	U	#N/A
Pox 2012	NRS	TConly	YES	Critical	?	?	?	U	U	?
Pox 2012	NRS	TConly	YES	Critical	?	?	?	U	U	?
Quintero 2012	RCT	TConly	YES	Critical	U	?	?	U	U	?
Rutter 2012	NRS	TConly	YES	Critical	?	?	?	U	U	?
Schoenfeld 2005	NRS	TConly	YES	Critical	?	?	?	U	U	?
Nelson 2002	NRS	TConly		Serious	?	?	?	U	U	?
Garcia-Albeniz 2017	NRS	TConly		Serious	?	?	?	U	U	?
Garcia-Albeniz 2017	NRS	TConly		Serious	?	?	?	U	U	?
Pedersen 2020	NRS	TConly		Serious	?	?	?	U	U	?
Pedersen 2020	NRS	TConly		Serious	?	?	?	U	U	?
Xirasagar 2020	NRS	TConly		Serious	O	O	?	?	U	U
Khalid-de Bakker 2011	NRS	TConly		Serious	?	?	?	?	U	?
Khalid-de Bakker 2011b	NRS	TConly		Serious	?	?	?	U	U	?
Causada-Calo 2020	NRS	TConly		Serious	U	?	?	U	U	?
Zwink 2017	NRS	TConly		Serious	?	?	?	?	?	?
Senore 2011	RCT	TConly	YES	Critical	?	?	?	U	U	U
Waldmann 2016	NRS	TConly		Serious	?	?	U	U	U	?
Ferlitsch 2011	NRS	TConly		Serious	?	?	?	?	U	?
**Zwink 2017**	NRS	TConly		Serious	?	?	?	?	U	?
Ahmed 2016	NRS	TConly		Serious	?	?	U	?	U	U
Sieg 2006	NRS	TConly	YES	Critical	?	?	?	U	U	?
Stoop 2012	RCT	TConly	YES	Critical	?	?	?	U	U	U
Nelson 2002	NRS	TConly		Serious	?	?	?	U	U	?
Stock 2013	NRS	TConly		Serious	?	?	?	U	U	?
Garcia-Albeniz 2017	NRS	TConly		Serious	?	?	?	U	U	?
Garcia-Albeniz 2017	NRS	TConly		Serious	?	?	?	U	U	?
Pedersen 2020	NRS	TConly		Serious	?	?	?	U	U	?
Wang 2018	NRS	TConly		Serious	?	?	?	U	U	?
Wang 2018	NRS	TConly		Serious	?	?	?	U	U	?
Lieberman 2000	NRS	TConly		Serious	?	?	U	U	U	?
Strul 2006	NRS	TConly	YES	Critical	?	U	?	U	U	?
Wong 2017	NRS	TConly	YES	Critical	O	?	?	U	U	?
Bokemeyer 2009	NRS	TConly		Serious	U	?	?	U	U	U
Taleban 2018	NRS	TConly		Serious	?	?	?	?	U	?
Bretthauer 2016	RCT	TConly		Serious	?	?	U	U	U	?

17 Appendix 17 - Bias distributions across all studies that assess perforation

Study ID	Design	Procedure	Critical	Worst score	Inception	Classification	Performance	Missing data	Measurement	Reporting
Atkin 2002	RCT	FS		Serious	?	U	?	?	U	?
Forbes 2006	RCT	FS		Serious	?	U	U	U	?	?
Holme 2014	RCT	FS	YES	Critical	?	U	U	U	U	U
Jain 2002	NRS	FS	YES	Critical	?	U	U	U	U	?
Kewenter 1996	RCT	FS	YES	Critical	?	U	U	?	U	?
Levin 2002	NRS	FS		Serious	?	U	U	U	U	?
Randel 2021	RCT	FS		Serious	?	U	?	?	U	?
Schoen 2012	RCT	FS	YES	Critical	U	?	?	U	U	?
Segnan 2002	RCT	FS	YES	Critical	?	U	?	U	U	?
Zubarik 2002	NRS	FS	YES	Critical	?	?	?	U	U	?
Arana-Arri 2018	NRS	TCfobt	YES	Critical	?	?	?	U	U	?
Benazzato 2020	NRS	TCfobt		Moderate	?	?	?	?	?	?
Binefa 2013	NRS	TCfobt	YES	Critical	U	?	?	U	U	?
Cheng 2002	NRS	TCfobt	YES	Critical	?	?	?	?	U	?
Dancourt 2008	NRS	TCfobt	YES	Critical	?	?	?	U	U	?
Denis 2007	NRS	TCfobt	YES	Critical	?	?	?	U	U	?
Denis 2013	NRS	TCfobt		Moderate	?	?	?	U	?	?
Denis 2021	NRS	TCfobt		Serious	?	U	?	U	U	?
Denis 2021	NRS	TCfobt		Serious	?	U	?	U	U	?
Denters 2012	NRS	TCfobt	YES	Critical	U	?	?	?	U	?
Denters 2013	NRS	TCfobt	YES	Critical	U	?	?	U	U	?
Derbyshire 2018	NRS	TCfobt		Serious	?	?	?	U	U	?
Din 2015	NRS	TCfobt		Serious	U	?	?	U	U	?
Din 2017	NRS	TCfobt	YES	Critical	?	?	?	U	U	?
Dominitz 2019	RCT	TCfobt		Serious	?	?	?	?	?	?
Ellul 2010	NRS	TCfobt	YES	Critical	?	?	?	U	U	?
Faivre 2004	NRS	TCfobt	YES	Critical	?	?	?	U	U	?
Florido 2017	NRS	TCfobt	YES	Critical	?	?	?	?	?	?
Garcia 2012	NRS	TCfobt	YES	Critical	?	U	?	U	U	?
Gupta 2012	NRS	TCfobt		Serious	U	?	?	U	U	?
Hsu 2020	NRS	TCfobt		Serious	?	?	?	U	U	?
Ibánez 2018	NRS	TCfobt		Serious	?	?	?	U	U	?
Karlijn 2021	NRS	TCfobt		Serious	?	?	?	U	U	?
Lee 2012	NRS	TCfobt		Serious	?	?	U	U	U	?
Marino 2012	NRS	TCfobt	YES	Critical	?	?	?	U	U	?
Meulen 2021	NRS	TCfobt	YES	Critical	?	?	?	U	U	?
Mikkelsen 2018	NRS	TCfobt		Serious	?	?	?	U	U	?
Neely 2013	NRS	TCfobt	YES	Critical	?	?	?	U	U	?
Parente 2013	NRS	TCfobt	YES	Critical	?	?	?	U	U	?
Paszat 2020	NRS	TCfobt		Serious	?	U	?	U	?	?
Portillo 2018	NRS	TCfobt		Serious	?	?	?	?	U	?
Quintero 2012	RCT	TCfobt	YES	Critical	?	?	?	U	U	?
Quyn 2018	NRS	TCfobt	YES	Critical	?	?	?	?	U	?
Quyn 2018	NRS	TCfobt	YES	Critical	?	?	?	?	U	?
Quyn 2018	NRS	TCfobt	YES	Critical	?	?	?	?	U	?
Randel 2021	RCT	TCfobt		Serious	?	U	U	?	U	?
Robertson 2019	RCT	TCfobt		Serious	?	U	?	?	?	?
Robinson 1999	RCT	TCfobt	YES	Critical	U	?	?	?	?	?
Rutter 2014	NRS	TCfobt		Serious	U	O	U	U	U	?
Saraste 2016	NRS	TCfobt		Serious	?	U	?	U	U	?
Steele 2004	NRS	TCfobt	YES	Critical	?	?	?	?	U	?
Sung 2003	NRS	TCfobt		Serious	U	?	?	U	U	?
Tepes 2017	NRS	TCfobt		Serious	?	?	?	?	U	?
Tomaszewski 2021	NRS	TCfobt		Serious	?	U	?	U	U	?
Vanaclocha-Espi 2018	NRS	TCfobt		Serious	?	?	?	U	U	?
Zorzi 2009	NRS	TCfobt	YES	Critical	U	U	U	U	U	U
Atkin 2002	RCT	TCfollowup		Serious	?	?	?	?	U	?
Castro 2013	NRS	TCfollowup	YES	Critical	?	?	?	U	U	?
Dellon 2009	NRS	TCfollowup		Serious	?	U	?	U	U	?
Forbes 2006	RCT	TCfollowup		Serious	?	?	?	U	?	?
Gondal 2003	RCT	TCfollowup	YES	Critical	?	?	?	U	U	?
Holme 2014	RCT	TCfollowup	YES	Critical	?	?	?	U	U	U
Kewenter 1996	RCT	TCfollowup	YES	Critical	?	?	?	?	U	?
Mandel 1993	RCT	TCfollowup	YES	Critical	?	?	?	?	U	?
Naumann 2021	NRS	TCfollowup	YES	Critical	?	U	?	?	U	?
Polter 2015	NRS	TCfollowup	YES	Critical	?	U	?	U	U	?
Randel 2021	RCT	TCfollowup		Serious	?	U	?	?	U	?
Rutter 2012	NRS	TCfollowup	YES	Critical	?	?	?	U	U	?
Schoen 2012	RCT	TCfollowup	YES	Critical	U	?	?	U	U	?
Segnan 2002	RCT	TCfollowup	YES	Critical	?	?	?	U	U	?
Shroff 2015	NRS	TCfollowup	YES	Critical	?	U	?	U	U	?
Ahmed 2016	NRS	TConly		Serious	?	?	U	?	U	U
Berhane 2009	NRS	TConly	YES	Critical	O	?	?	U	U	?
Bielawska 2014	NRS	TConly		Serious	?	U	?	U	U	?
Bokemeyer 2009	NRS	TConly		Serious	U	?	?	U	U	U
Bretthauer 2016	RCT	TConly		Moderate	?	?	U	U	U	?
Causada-Calo 2020	NRS	TConly		Serious	U	?	?	U	U	?
Chiu 2013	NRS	TConly	YES	Critical	?	?	?	U	U	?
Crispin 2009	NRS	TConly	YES	Critical	?	?	?	U	U	?
Dae 2007	NRS	TConly	YES	Critical	?	?	?	U	U	?
Ferlitsch 2011	NRS	TConly		Serious	?	?	?	?	U	?
Garcia-Albeniz 2017	NRS	TConly		Serious	?	?	?	U	U	?
Garcia-Albeniz 2017	NRS	TConly		Serious	?	?	?	U	U	?
Hamdani 2013	NRS	TConly	YES	Critical	?	U	?	U	U	?
Huppe 2004	NRS	TConly	YES	Critical	?	?	?	U	U	?
Imperiale 2000	NRS	TConly	YES	Critical	?	?	?	?	U	?
Khalid-de Bakker 2011	NRS	TConly		Serious	?	?	?	?	U	?
Khalid-de Bakker 2011b	NRS	TConly		Serious	?	?	?	U	U	?
Leventi 2021	NRS	TConly	YES	Critical	?	?	?	U	U	?
Lieberman 2000	NRS	TConly		Serious	?	?	U	U	U	?
Nelson 2002	NRS	TConly		Serious	?	?	?	U	U	?
Pedersen 2020	NRS	TConly		Serious	?	?	?	U	U	?
Pedersen 2020	NRS	TConly		Serious	?	?	?	U	U	?
Pedersen 2020	NRS	TConly		Serious	?	?	?	U	U	?
Pox 2012	NRS	TConly	YES	Critical	?	?	?	U	U	?
Quintero 2012	RCT	TConly	YES	Critical	U	?	?	U	U	?
Rutter 2012	NRS	TConly	YES	Critical	?	?	?	U	U	?
Schoenfeld 2005	NRS	TConly	YES	Critical	?	?	?	U	U	?
Sieg 2006	NRS	TConly	YES	Critical	?	?	?	U	U	?
Stock 2013	NRS	TConly		Serious	?	?	?	U	U	?
Strul 2006	NRS	TConly	YES	Critical	?	U	?	U	U	?
Taleban 2018	NRS	TConly		Serious	?	?	?	?	U	?
Waldmann 2016	NRS	TConly		Serious	?	?	U	U	U	?

Wang 2018	NRS	TConly		Serious	?	?	?	U	U	?
Xirasagar 2020	NRS	TConly		Serious	O	O	?	?	U	U
Zubarik 2002	NRS	TConly	YES	Critical	?	?	?	U	U	?
**Zwink 2017**	NRS	TConly		Serious	?	?	?	?	?	?
**Zwink 2017**	NRS	TConly		Serious	?	?	?	?	U	?

18 Appendix 18 – Characteristics of the external validity for bleeding

**Procedure**	**Sigmoidoscopy**				
**Outcome**	**Bleeding**				
**Subcategory**	**Severe-longterm**				
**No. Subcat.**	**People**	**Events**	**Risk/100.000**	**Quality**	**Grading SUM**
1	36065	3	8	very low	-3
**Risk of bias**	**Inconsistency**	**Indirectness**	**Imprecision**	**Publications bias**	**Large effect**
-2	NA	NA	0	-1	0
**Reasons for downgrading due to Inconsistency**	**0**		**OIS**	**12022**	
Measurement tool	NO				
Outcome assessor	NO				
Country/setting	NO				
Study year varies > 10 years	NO				
Age distribution	NO				
Expertise of endoscopists	NO				
With/without anaesthesia	NO				
Varying polypectomy rate	NO				

**Procedure**	**Sigmoidoscopy**				
**Outcome**	**Bleeding**				
**Subcategory**	**ND-longterm**				
**No. Subcat.**	**People**	**Events**	**Risk/100.000**	**Quality**	**Grading SUM**
2	107743	2	2	very low	-4
**Risk of bias**	**Inconsistency**	**Indirectness**	**Imprecision**	**Publications bias**	**Large effect**
-2	0	NA	-1	-1	0
**Reasons for downgrading due to inconsistency**	**0**		**OIS**		
Measurement tool	NO		**53872**		
Outcome assessor	NO				

Country/setting	NO				
Study year varies > 10 years	NO				
Age distribution	NO				
Expertise of endoscopists	NO				
With/without anaesthesia	NO				
Varying polypectomy rate	NO				

**Procedure**	**Sigmoidoscopy**				
**Outcome**	**Bleeding**				
**Subcategory**	**Mild-longterm**				
**No. Subcat.**	**People**	**Events**	**Risk/100.000**	**Quality**	**Grading SUM**
1	40674	77	189	very low	-2
**Risk of bias**	**Inconsistency**	**Indirectness**	**Imprecision**	**Publications bias**	**Large effect**
-2	NA	NA	0	-1	1
**Reasons for downgrading due to inconsistency**	**0**		**OIS**		
Measurement tool	NO		**528**		
Outcome assessor	NO				
Country/setting	NO				
Study year varies > 10 years	NO				
Age distribution	NO				
Expertise of endoscopists	NO				
With/without anaesthesia	NO				
Varying polypectomy rate	NO				

**Procedure**	**Sigmoidoscopy**				
**Outcome**	**Bleeding**				
**Subcategory**	**Severe-NR**				
**No. Subcat.**	**People**	**Events**	**Risk/100.000**	**Quality**	**Grading SUM**
1	40674	30	30	very low	-2
**Risk of bias**	**Inconsistency**	**Indirectness**	**Imprecision**	**Publications bias**	**Large effect**
-2	NA	NA	0	-1	1
**Reasons for downgrading due to inconsistency**	**0**		**OIS**		

Measurement tool	NO		**1356**		
Outcome assessor	NO				
Country/setting	NO				
Study year varies > 10 years	NO				
Age distribution	NO				
Expertise of endoscopists	NO				
With/without anaesthesia	NO				
Varying polypectomy rate	NO				

**Procedure**	**Sigmoidoscopy**				
**Outcome**	**Bleeding**				
**Subcategory**	**ND-NR**				
**No. Subcat.**	**People**	**Events**	**Risk/100.000**	**Quality**	**Grading SUM**
1	6968	3	43	very low	-4
**Risk of bias**	**Inconsistency**	**Indirectness**	**Imprecision**	**Publications bias**	**Large effect**
-2	NA	NA	-1	-1	0
**Reasons for downgrading due to inconsistency**	**0**		**OIS**		
Measurement tool	NO		**2323**		
Outcome assessor	NO				
Country/setting	NO				
Study year varies > 10 years	NO				
Age distribution	NO				
Expertise of endoscopists	NO				
With/without anaesthesia	NO				
Varying polypectomy rate	NO				

**Procedure**	**Colonoscopy following FIT**				
**Outcome**	**Bleeding**				
**Subcategory**	**Severe-longterm**				
**No. Subcat.**	**People**	**Events**	**Risk/100.000**	**Quality**	**Grading SUM**
14	674094	3456	229	very low	-6
**Risk of bias**	**Inconsistency**	**Indirectness**	**Imprecision**	**Publications bias**	**Large effect**
-2	-2	NA	-1	-1	0

Reasons for downgrading due to inconsistency	5		OIS		
Measurement tool	NO		195		
Outcome assessor	YES				
Country/setting	YES				
Study year varies > 10 years	NO				
Age distribution	NO				
Expertise of endoscopists	YES				
With/without anaesthesia	YES				
Varying polypectomy rate	YES				

**Procedure**	**Colonoscopy following FIT**				
**Outcome**	**Bleeding**				
**Subcategory**	**ND-longterm**				
**No. Subcat.**	**People**	**Events**	**Risk/100.000**	**Quality**	**Grading SUM**
5	172393,5	1295	793	very low	-2
**Risk of bias**	**Inconsistency**	**Indirectness**	**Imprecision**	**Publications bias**	**Large effect**
-2	0	NA	-1	-1	2
**Reasons for downgrading due to inconsistency**	**0**		**OIS**		
Measurement tool	NO		**133**		
Outcome assessor	NO				
Country/setting	NO				
Study year varies > 10 years	NO				
Age distribution	NO				
Expertise of endoscopists	NO				
With/without anaesthesia	NO				
Varying polypectomy rate	NO				

**Procedure**	**Colonoscopy following FIT**				
**Outcome**	**Bleeding**				
**Subcategory**	**Mild-longterm**				
**No. Subcat.**	**People**	**Events**	**Risk/100.000**	**Quality**	**Grading SUM**
4	177490	1249	631	very low	-4
**Risk of bias**	**Inconsistency**	**Indirectness**	**Imprecision**	**Publications bias**	**Large effect**

-2	-2	NA	-1	-1	2
**Reasons for downgrading due to inconsistency**	**6**		**OIS**		
Measurement tool	YES		**142**		
Outcome assessor	YES				
Country/setting	YES				
Study year varies > 10 years	NO				
Age distribution	YES				
Expertise of endoscopists	YES				
With/without anaesthesia	NO				
Varying polypectomy rate	YES				

**Procedure**	**Colonoscopy following FIT**				
**Outcome**	**Bleeding**				
**Subcategory**	**Mild-NR**				
**No. Subcat.**	**People**	**Events**	**Risk/100.000**	**Quality**	**Grading SUM**
1	50858	3865	7600	very low	-1
**Risk of bias**	**Inconsistency**	**Indirectness**	**Imprecision**	**Publications bias**	**Large effect**
-2	NA	NA	0	-1	2
**Reasons for downgrading due to inconsistency**	**0**		**OIS**		
Measurement tool	NO		**13**		
Outcome assessor	NO				
Country/setting	NO				
Study year varies > 10 years	NO				
Age distribution	NO				
Expertise of endoscopists	NO				
With/without anaesthesia	NO				
Varying polypectomy rate	NO				

**Procedure**	**Colonoscopy following FIT**				
**Outcome**	**Bleeding**				
**Subcategory**	**ND-NR**				
**No. Subcat.**	**People**	**Events**	**Risk/100.000**	**Quality**	**Grading SUM**
1	476	1	210	very low	-4

Risk of bias	Inconsistency	Indirectness	Imprecision	Publications bias	Large effect
-2	NA	NA	-1	-1	0
**Reasons for downgrading due to inconsistency**	**0**		**OIS**		
Measurement tool	NO		**476**		
Outcome assessor	NO				
Country/setting	NO				
Study year varies > 10 years	NO				
Age distribution	NO				
Expertise of endoscopists	NO				
With/without anaesthesia	NO				
Varying polypectomy rate	NO				

**Procedure**	**Colonoscopy following FIT**				
**Outcome**	**Bleeding**				
**Subcategory**	**Severe-NR**				
**No. Subcat.**	**People**	**Events**	**Risk/100.000**	**Quality**	**Grading SUM**
1	1057	8	757	very low	-2
**Risk of bias**	**Inconsistency**	**Indirectness**	**Imprecision**	**Publications bias**	**Large effect**
-2	NA	NA	-1	-1	2
**Reasons for downgrading due to inconsistency**	**0**		**OIS**		
Measurement tool	NO		**132**		
Outcome assessor	NO				
Country/setting	NO				
Study year varies > 10 years	NO				
Age distribution	NO				
Expertise of endoscopists	NO				
With/without anaesthesia	NO				
Varying polypectomy rate	NO				

**Procedure**	**Colonoscopy following any screening tests**				
**Outcome**	**Bleeding**				
**Subcategory**	**Mild-NR**				
**No. Subcat.**	**People**	**Events**	**Risk/100.000**	**Quality**	**Grading SUM**

1	2051	7	341	very low	-4
**Risk of bias**	**Inconsistency**	**Indirectness**	**Imprecision**	**Publications bias**	**Large effect**
-2	NA	NA	-1	-1	0
**Reasons for downgrading due to inconsistency**	**0**		**OIS**		
Measurement tool	NO		**293**		
Outcome assessor	NO				
Country/setting	NO				
Study year varies > 10 years	NO				
Age distribution	NO				
Expertise of endoscopists	NO				
With/without anaesthesia	NO				
Varying polypectomy rate	NO				

**Procedure**	**Colonoscopy following any screening tests**				
**Outcome**	**Bleeding**				
**Subcategory**	**ND-longterm**				
**No. Subcat.**	**People**	**Events**	**Risk/100.000**	**Quality**	**Grading SUM**
1	112	0	0	very low	-5
**Risk of bias**	**Inconsistency**	**Indirectness**	**Imprecision**	**Publications bias**	**Large effect**
-2	NA	NA	-2	-1	0
**Reasons for downgrading due to inconsistency**	**0**		**OIS**		
Measurement tool	NO		**NA**		
Outcome assessor	NO				
Country/setting	NO				
Study year varies > 10 years	NO				
Age distribution	NO				
Expertise of endoscopists	NO				
With/without anaesthesia	NO				
Varying polypectomy rate	NO				

**Procedure**	**Colonoscopy following any screening tests**
**Outcome**	**Bleeding**
**Subcategory**	**Severe-longterm**

No. Subcat.	People	Events	Risk/100.000	Quality	Grading SUM
1	3297	23	698	very low	-3
**Risk of bias**	**Inconsistency**	**Indirectness**	**Imprecision**	**Publications bias**	**Large effect**
-2	NA	NA	-1	-1	1
**Reasons for downgrading due to inconsistency**	**0**		**OIS**		
Measurement tool	NO		**143**		
Outcome assessor	NO				
Country/setting	NO				
Study year varies > 10 years	NO				
Age distribution	NO				
Expertise of endoscopists	NO				
With/without anaesthesia	NO				
Varying polypectomy rate	NO				

**Procedure**	**Colonoscopy following any screening tests**				
**Outcome**	**Bleeding**				
**Subcategory**	**Severe-NR**				
**No. Subcat.**	**People**	**Events**	**Risk/100.000**	**Quality**	**Grading SUM**
1	2051	9	439	very low	-2
**Risk of bias**	**Inconsistency**	**Indirectness**	**Imprecision**	**Publications bias**	**Large effect**
-2	NA	NA	0	-1	1
**Reasons for downgrading due to inconsistency**	**0**		**OIS**		
Measurement tool	NO		**228**		
Outcome assessor	NO				
Country/setting	NO				
Study year varies > 10 years	NO				
Age distribution	NO				
Expertise of endoscopists	NO				
With/without anaesthesia	NO				
Varying polypectomy rate	NO				

**Procedure**	**Once-only colonoscopy**
**Outcome**	**Bleeding**

Subcategory	Severe-longterm				
No. Subcat.	People	Events	Risk/100.000	Quality	Grading SUM
7	749296	459	68	very low	-6
Risk of bias	Inconsistency	Indirectness	Imprecision	Publications bias	Large effect
-2	-2	NA	-1	-1	0
Reasons for downgrading due to inconsistency	6		OIS		
Measurement tool	YES		1632		
Outcome assessor	YES				
Country/setting	YES				
Study year varies > 10 years	YES				
Age distribution	YES				
Expertise of endoscopists	YES				
With/without anaesthesia	NO				
Varying polypectomy rate	NO				

Procedure	Once-only colonoscopy				
Outcome	Bleeding				
Subcategory	Mild-longterm				
No. Subcat.	People	Events	Risk/100.000	Quality	Grading SUM
4	92424	257	320	very low	-5
Risk of bias	Inconsistency	Indirectness	Imprecision	Publications bias	Large effect
-2	-1	NA	-1	-1	0
Reasons for downgrading due to inconsistency	3		OIS		
Measurement tool	YES		360		
Outcome assessor	YES				
Country/setting	YES				
Study year varies > 10 years	NO				
Age distribution	NO				
Expertise of endoscopists	NO				
With/without anaesthesia	NO				
Varying polypectomy rate	NO				

**Procedure**	**Once-only colonoscopy**				
**Outcome**	**Bleeding**				
**Subcategory**	**ND-longterm**				
**No. Subcat.**	**People**	**Events**	**Risk/100.000**	**Quality**	**Grading SUM**
4	36356	111	420	very low	-5
**Risk of bias**	**Inconsistency**	**Indirectness**	**Imprecision**	**Publications bias**	**Large effect**
-2	-1	NA	-1	-1	0
**Reasons for downgrading due to inconsistency**	**3**		**OIS**		
Measurement tool	YES		**328**		
Outcome assessor	NO				
Country/setting	YES				
Study year varies > 10 years	NO				
Age distribution	NO				
Expertise of endoscopists	NO				
With/without anaesthesia	NO				
Varying polypectomy rate	YES				

**Procedure**	**Once-only colonoscopy**				
**Outcome**	**Bleeding**				
**Subcategory**	**ND-NR**				
**No. Subcat.**	**People**	**Events**	**Risk/100.000**	**Quality**	**Grading SUM**
4	209897	239	150	very low	-5
**Risk of bias**	**Inconsistency**	**Indirectness**	**Imprecision**	**Publications bias**	**Large effect**
-2	-1	NA	-1	-1	0
**Reasons for downgrading due to inconsistency**	**3**		**OIS**		
Measurement tool	NO		**878**		
Outcome assessor	NO				
Country/setting	YES				
Study year varies > 10 years	NO				
Age distribution	YES				
Expertise of endoscopists	NO				
With/without anaesthesia	NO				
Varying polypectomy rate	YES				

**Procedure**	**Once-only colonoscopy**				
**Outcome**	**Bleeding**				
**Subcategory**	**Severe-NR**				
**No. Subcat.**	**People**	**Events**	**Risk/100.000**	**Quality**	**Grading SUM**
3	276613	461	167	very low	-2
**Risk of bias**	**Inconsistency**	**Indirectness**	**Imprecision**	**Publications bias**	**Large effect**
-2	0	NA	0	-1	1
**Reasons for downgrading due to inconsistency**	**0**		**OIS**		
Measurement tool	NO		**600**		
Outcome assessor	NO				
Country/setting	NO				
Study year varies > 10 years	NO				
Age distribution	NO				
Expertise of endoscopists	NO				
With/without anaesthesia	NO				
Varying polypectomy rate	NO				

**Procedure**	**Once-only colonoscopy**				
**Outcome**	**Bleeding**				
**Subcategory**	**Mild-NR**				
**No. Subcat.**	**People**	**Events**	**Risk/100.000**	**Quality**	**Grading SUM**
1	25008	6	24	very low	-2
**Risk of bias**	**Inconsistency**	**Indirectness**	**Imprecision**	**Publications bias**	**Large effect**
-2	NA	NA	0	-1	1
**Reasons for downgrading due to inconsistency**	**0**		**OIS**		
Measurement tool	NO		**4168**		
Outcome assessor	NO				
Country/setting	NO				
Study year varies > 10 years	NO				
Age distribution	NO				
Expertise of endoscopists	NO				
With/without anaesthesia	NO				

Varying polypectomy rate	NO				

19 Appendix 19 – Characteristics of the external validity for perforation

**Procedure**	**Sigmoidoscopy**				
**Outcome**	**Perforation**				
**Subcategory**	**ND-longterm**				
**No. Subcat.**	**People**	**Events**	**Risk/100.000**	**Quality**	**Grading SUM**
2	107743	2	2	very low	-4
**Risk of bias**	**Inconsistency**	**Indirectness**	**Imprecision**	**Publications bias**	**Large effect**
-2	0	NA	-1	-1	0
**Reasons for downgrading due to inconsistency**	**0**		**OIS**		
Measurement tool	NO		**53872**		
Outcome assessor	NO				
Country/setting	NO				
Study year varies > 10 years	NO				
Age distribution	NO				
Expertise of endoscopists	NO				
With/without anaesthesia	NO				
Varying polypectomy rate	NO				

**Procedure**	**Sigmoidoscopy**				
**Outcome**	**Perforation**				
**Subcategory**	**ND-NR**				
**No. Subcat.**	**People**	**Events**	**Risk/100.000**	**Quality**	**Grading SUM**
1	40674	1	2	very low	-4
**Risk of bias**	**Inconsistency**	**Indirectness**	**Imprecision**	**Publications bias**	**Large effect**
-2	NA	NA	-1	-1	0
**Reasons for downgrading due to inconsistency**	**0**		**OIS**		
Measurement tool	NO		**40674**		
Outcome assessor	NO				
Country/setting	NO				

Study year varies > 10 years	NO				
Age distribution	NO				
Expertise of endoscopists	NO				
With/without anaesthesia	NO				
Varying polypectomy rate	NO				

**Procedure**	**Sigmoidoscopy**				
**Outcome**	**Perforation**				
**Subcategory**	**Mild-longterm**				
**No. Subcat.**	**People**	**Events**	**Risk/100.000**	**Quality**	**Grading SUM**
1	36065	3	8	very low	-4
**Risk of bias**	**Inconsistency**	**Indirectness**	**Imprecision**	**Publications bias**	**Large effect**
-2	NA	NA	-1	-1	0
**Reasons for downgrading due to inconsistency**	**0**		**OIS**		
Measurement tool	NO		**12022**		
Outcome assessor	NO				
Country/setting	NO				
Study year varies > 10 years	NO				
Age distribution	NO				
Expertise of endoscopists	NO				
With/without anaesthesia	NO				
Varying polypectomy rate	NO				

**Procedure**	**Colonoscopy following FIT**				
**Outcome**	**Perforation**				
**Subcategory**	**Severe-longterm**				
**No. Subcat.**	**People**	**Events**	**Risk/100.000**	**Quality**	**Grading SUM**
12	682112,1	745	88	very low	-4
**Risk of bias**	**Inconsistency**	**Indirectness**	**Imprecision**	**Publications bias**	**Large effect**
-2	-2	NA	-1	-1	2
**Reasons for downgrading due to inconsistency**	**5**		**OIS**		
Measurement tool	YES		**916**		

Outcome assessor	YES				
Country/setting	YES				
Study year varies > 10 years	NO				
Age distribution	NO				
Expertise of endoscopists	YES				
With/without anaesthesia	YES				
Varying polypectomy rate	NO				

**Procedure**	**Colonoscopy following FIT**				
**Outcome**	**Perforation**				
**Subcategory**	**ND-longterm**				
**No. Subcat.**	**People**	**Events**	**Risk/100.000**	**Quality**	**Grading SUM**
6	428265,8	301	70	very low	-1
**Risk of bias**	**Inconsistency**	**Indirectness**	**Imprecision**	**Publications bias**	**Large effect**
-2	0	NA	0	-1	2
**Reasons for downgrading due to inconsistency**	**0**		**OIS**		
Measurement tool	NO		**1423**		
Outcome assessor	NO				
Country/setting	NO				
Study year varies > 10 years	NO				
Age distribution	NO				
Expertise of endoscopists	NO				
With/without anaesthesia	NO				
Varying polypectomy rate	NO				

**Procedure**	**Colonoscopy following FIT**				
**Outcome**	**Perforation**				
**Subcategory**	**ND-NR**				
**No. Subcat.**	**People**	**Events**	**Risk/100.000**	**Quality**	**Grading SUM**
3	15207	8	53	very low	-2
**Risk of bias**	**Inconsistency**	**Indirectness**	**Imprecision**	**Publications bias**	**Large effect**
-2	0	NA	-1	-1	2

**Reasons for downgrading due to incosnsistency**	**0**		**OIS**		
Measurement tool	NO		**1901**		
Outcome assessor	NO				
Country/setting	NO				
Study year varies > 10 years	NO				
Age distribution	NO				
Expertise of endoscopists	NO				
With/without anaesthesia	NO				
Varying polypectomy rate	NO				

**Procedure**	**Colonoscopy following FIT**				
**Outcome**	**Perforation**				
**Subcategory**	**Mild-longterm**				
**No. Subcat.**	**People**	**Events**	**Risk/100.000**	**Quality**	**Grading SUM**
2	85776	51	59	very low	-1
**Risk of bias**	**Inconsistency**	**Indirectness**	**Imprecision**	**Publications bias**	**Large effect**
-2	0	NA	0	-1	2
**Reasons for downgrading due to inconsistency**	**0**		**OIS**		
Measurement tool	NO		**1682**		
Outcome assessor	NO				
Country/setting	NO				
Study year varies > 10 years	NO				
Age distribution	NO				
Expertise of endoscopists	NO				
With/without anaesthesia	NO				
Varying polypectomy rate	NO				

**Procedure**	**Once-only colonoscopy**				
**Outcome**	**Perforation**				
**Subcategory**	**ND-NR**				
**No. Subcat.**	**People**	**Events**	**Risk/100.000**	**Quality**	**Grading SUM**
7	782103	90	12	very low	-2

Risk of bias	Inconsistency	Indirectness	Imprecision	Publications bias	Large effect
-2	0	NA	0	-1	1
**Reasons for downgrading due to inconsistency**	**0**		**OIS**		
Measurement tool	NO		**8690**		
Outcome assessor	NO				
Country/setting	NO				
Study year varies > 10 years	NO				
Age distribution	NO				
Expertise of endoscopists	NO				
With/without anaesthesia	NO				
Varying polypectomy rate	NO				

**Procedure**	**Once-only colonoscopy**				
**Outcome**	**Perforation**				
**Subcategory**	**Severe-longterm**				
**No. Subcat.**	**People**	**Events**	**Risk/100.000**	**Quality**	**Grading SUM**
7	776620,4	268	53	very low	-3
**Risk of bias**	**Inconsistency**	**Indirectness**	**Imprecision**	**Publications bias**	**Large effect**
-2	-2	NA	0	-1	2
**Reasons for downgrading due to inconsistency**	**4**		**OIS**		
Measurement tool	NO		**2898**		
Outcome assessor	NO				
Country/setting	YES				
Study year varies > 10 years	NO				
Age distribution	YES				
Expertise of endoscopists	YES				
With/without anaesthesia	NO				
Varying polypectomy rate	YES				

**Procedure**	**Once-only colonoscopy**
**Outcome**	**Perforation**

Subcategory	ND-longterm				
No. Subcat.	People	Events	Risk/100.000	Quality	Grading SUM
4	9034	8	53	very low	-3
Risk of bias	Inconsistency	Indirectness	Imprecision	Publications bias	Large effect
-2	0	NA	-1	-1	1
**Reasons for downgrading due to inconsistency**	**0**		**OIS**		
Measurement tool	NO		**1129**		
Outcome assessor	NO				
Country/setting	NO				
Study year varies > 10 years	NO				
Age distribution	NO				
Expertise of endoscopists	NO				
With/without anaesthesia	NO				
Varying polypectomy rate	NO				

Procedure	Once-only colonoscopy				
Outcome	Perforation				
Subcategory	Severe-NR				
No. Subcat.	People	Events	Risk/100.000	Quality	Grading SUM
2	264701	56	70	very low	-3
Risk of bias	Inconsistency	Indirectness	Imprecision	Publications bias	Large effect
-2	0	NA	-1	-1	1
**Reasons for downgrading due to inconsistency**	**0**		**OIS**		
Measurement tool	NO		**4727**		
Outcome assessor	NO				
Country/setting	NO				
Study year varies > 10 years	NO				
Age distribution	NO				
Expertise of endoscopists	NO				
With/without anaesthesia	NO				
Varying polypectomy rate	NO				

Procedure	Once-only colonoscopy				
Outcome	Perforation				
Subcategory	Mild-longterm				
No. Subcat.	People	Events	Risk/100.000	Quality	Grading SUM
2	22327	16	72	very low	-2
Risk of bias	Inconsistency	Indirectness	Imprecision	Publications bias	Large effect
-2	0	NA	0	-1	1
**Reasons for downgrading due to Inconsistency**	**0**		**OIS**	**1395**	
Measurement tool	NO				
Outcome assessor	NO				
Country/setting	NO				
Study year varies > 10 years	NO				
Age distribution	NO				
Expertise of endoscopists	NO				
With/without anaesthesia	NO				
Varying polypectomy rate	NO				

Procedure	Colonoscopy following any screening tests				
Outcome	Perforation				
Subcategory	ND-NR				
No. Subcat.	People	Events	Risk/100.000	Quality	Grading SUM
2	5665	4	32	very low	-3
Risk of bias	Inconsistency	Indirectness	Imprecision	Publications bias	Large effect
-2	0	NA	-1	-1	1
**Reasons for downgrading due to inconsistency**	**0**		**OIS**		
Measurement tool	NO		**1416**		
Outcome assessor	NO				
Country/setting	NO				
Study year varies > 10 years	NO				
Age distribution	NO				
Expertise of endoscopists	NO				
With/without anaesthesia	NO				

Varying polypectomy rate	NO				

**Procedure**	**Colonoscopy following any screening tests**				
**Outcome**	**Perforation**				
**Subcategory**	**ND-longterm**				
**No. Subcat.**	**People**	**Events**	**Risk/100.000**	**Quality**	**Grading SUM**
1	112	0	0	very low	-3
**Risk of bias**	**Inconsistency**	**Indirectness**	**Imprecision**	**Publications bias**	**Large effect**
-2	NA	NA	NA	-1	0
**Reasons for downgrading due to inconsistency**	**0**		**OIS**		
Measurement tool	NO		**NA**		
Outcome assessor	NO				
Country/setting	NO				
Study year varies > 10 years	NO				
Age distribution	NO				
Expertise of endoscopists	NO				
With/without anaesthesia	NO				
Varying polypectomy rate	NO				

**Procedure**	**Colonoscopy following any screening tests**				
**Outcome**	**Perforation**				
**Subcategory**	**Mild-longterm**				
**No. Subcat.**	**People**	**Events**	**Risk/100.000**	**Quality**	**Grading SUM**
1	3297	4	121	very low	-2
**Risk of bias**	**Inconsistency**	**Indirectness**	**Imprecision**	**Publications bias**	**Large effect**
-2	NA	NA	0	-1	1
**Reasons for downgrading due to Inconsistency**	**0**		**OIS**	**824**	
Measurement tool	NO				
Outcome assessor	NO				
Country/setting	NO				
Study year varies > 10 years	NO				
Age distribution	NO				

Expertise of endoscopists	NO				
With/without anaesthesia	NO				
Varying polypectomy rate	NO				

20 Appendix 20 – The consequences of bleeding

Appendix 20: The consequences of bleeding.

	No. Subcategories	Proportion, %
No. subpopulations with > 0 events	108	88%
Information about consequence of harm*	39	36%
**Proportion calculated from no. subpopulations with > 0 events*		

Appendix 20: Reported consequences of bleeding events for subcategories.

Consequences	Number of subcategories reporting the respective consequence
Transfusion	14 (11.4%)
Treatment (surgery)	23 (18.7%)
Hospitalization	18 (14.6%)

21 Appendix 21 – The consequences of perforation

Appendix 21: The consequences of perforation.

	No. Subcategories	Proportion, %
No. subcategories with > 0 events	82	76%
Information about consequence of harm*	33	40%
**Proportion calculated from no. subpopulations with > 0 events*		

Appendix 21: Reported consequences of perforation events for subcategories.

Consequences	Number of subcategories reporting the respective consequence
Death	4 (3.7%)
Treatment (surgery)	22 (20.4%)
Morbidity	2 (1.8%)
Hospitalization	8 (7.4%)

22 Appendix 22 - Factors potentially modifying occurrences of bleeding

Appendix 22 - Factors potentially modifying occurrences of bleeding.

Modifier	Subcategories with modifier assessment	Significant effect
**Age**	21	7
**Gender (Women)**	22	9
**Polypectomy**	23	19
**Caecal intubation**	4	2
**Race**	6	3
**Expertise**	12	6
**Comorbidities**	4	2
**Cancer**	1	1
**Sedation**	0	0
**Income**	0	0

23 Appendix 23 - Factors potentially modifying occurrences of perforation

Appendix 23: Factors potentially modifying occurrences of perforation

Modifier	Subcategories with modifier assessment	Significant effect
**Age**	19	8
**Gender (Women)**	19	9
**Polypectomy**	24	21
**Caecal intubation**	1	1
**Race**	3	2
**Expertise**	7	5
**Comorbidities**	5	3
**Cancer**	0	0
**Sedation**	1	1
**Income**	1	0

24 Appendix 24 – Bleeding and perforation assessed in six former systematic reviews

Appendix 24: Six systematic reviews of bleeding and perforation.

Study ID	Lin 2021 (9)	Reumkens 2016 (10)	Fitzpatrick-Lewis 2016 (11)	Holme 2013 (12)	Vemeer 2017 (13)	Niv 2008 (14)
Sigmoidoscopy	YES	NO	YES	YES	NO	NO
Once-only colonoscopy	YES	YES	YES	NO	YES	YES
Colonoscopy following FIT	YES	NO	YES	YES	NO	NO
Colonoscopy following any screening tests	YES	NO	NO	NO	NO	NO
Perforation	YES	YES	YES	YES	YES	YES
Severe bleeding	YES	YES	YES	YES	YES	YES
Mild bleeding	NO	NO	YES	NO	NO	NO

25 Appendix 25 – Comparison between former systematic reviews that assess bleeding and current review.

Appendix 25: Occurrences of bleeding in former systematic reviews compared to the present review. Numbers are per 100,000 people screened with 95% CI.

	Sigmoidoscopy	Colonoscopy following FIT	Once-only colonoscopy	Colonoscopy following any screening tests
Present review (all types of bleedings with longterm follow-up)	56 [9;362]	445 [315;627]	268 [106;676]	205 [65;644]
Present review (all types of bleedings with	46 [6;336]	399 [203;782]	140 [111;177]	372 [178;774]

shortterm/not reported follow-up)				
Present review (severe events)	1 [0;32421]	247 [142;429]	63 [39;103]	198 [36;1082]
*Lin 2021 (severe events)	6 [0.0;153]	206 [(83;324]	149 [96-203]	211 [84;339]
Fitzpatrick-Lewis 2016 (severe events)	9 [4;15]	111 [65;175]	108 [85;132]	___
Holme 2013 (severe events)	7 [4;12]	72 [47;105]	___	___
*Reumkens 2016 (severe events)	___	___	284 [170;370]	___
*Vemeer 2017 (severe events)	___	___	78 [18;163]	___
§Niv 2008	___	___	5 [2;9]	___

Present review = this review

**Events/procedure converted to events/people*

§ Severity not defined

___ = *no harm estimates in the respective procedure*

26 Appendix 26 – Comparison between former systematic reviews that assess perforation and current review.

Appendix 26: Occurrences of perforation in former systematic reviews compared to the present review. Numbers are per 100,000 people screened with 95% CI.

	Sigmoidoscopy	Colonoscopy following FIT	Once-only colonoscopy	Colonoscopy following any screening tests
Present review (all types of	4 [2;611]	85 [62;115]	39 [38;39]	59 [26;134]

perforations with longterm follow-up)				
Present review (all types of perforations with shortterm/not reported follow-up)	5 [1;36]	83 [69;101]	22 [10;46]	100 [50;201]
Present review (severe events)	5 [1;36]	97 [62;152]	53 [26;112]	42 [13;138]
*§Lin 2021	2 [0.1;0.5]	64 [40;87]	32 [23;41]	122 [76;168]
§Fitzpatrick-Lewis 2016	1 [0;3]	61 [10;111]	53 [37;69]	___
Holme 2013 (severe events)	3 [1;7]	59 [37;89]	___	___
*§Reumkens 2016	___	___	50 [40;70]	___
*§Vemeer 2017	___	___	7 [0.6;17]	___
§Niv 2008	___	___	1 [0.6;2]	___

Present review = this review

**Events/procedure converted to events/people*

§ Severity not defined

___ = no harm estimates in the respective procedure

Reference list:

1. Martiny F, Gram EG, Nielsen SB, Rahbek O, Jauernik C, Bie AKL, et al. Physical harms associated with sigmoidoscopy and colonoscopy during colorectal cancer screening - a systematic review with meta-analyses of deaths and cardiopulmonary events. 2022.
2. Adler A, Lieberman D, Aminalai A, Aschenbeck J, Drossel R, Mayr M, et al. Data quality of the German Screening Colonoscopy Registry. Endoscopy. 2013 Sep 9;45(10):813–8.
3. Ladabaum U, Mannalithara A, Desai M, Sehgal M, Singh G. Age-Specific Rates and Time-Courses of Gastrointestinal and Nongastrointestinal Complications Associated With Screening/Surveillance Colonoscopy. Am J Gastroenterol. 2021 Dec;116(12):2430–45.

4. Stock C, Ihle P, Sieg A, Schubert I, Hoffmeister M, Brenner H. Adverse events requiring hospitalization within 30 days after outpatient screening and nonscreening colonoscopies. Gastrointest Endosc. 2013 Mar;77(3):419–29.
5. García-Albéniz X, Hsu J, Bretthauer M, Hernán MA. Effectiveness of Screening Colonoscopy to Prevent Colorectal Cancer Among Medicare Beneficiaries Aged 70 to 79 Years: A Prospective Observational Study. Ann Intern Med. 2017 Jan 3;166(1):18.
6. Hol L, van Leerdam ME, van Ballegooijen M, van Vuuren AJ, van Dekken H, Reijerink JCIY, et al. Screening for colorectal cancer: randomised trial comparing guaiac-based and immunochemical faecal occult blood testing and flexible sigmoidoscopy. Gut. 2010 Jan 1;59(01):62–8.
7. Pedersen L, Sorensen N, Lindorff-Larsen K, Carlsen CG, Wensel N, Torp-Pedersen C, et al. Colonoscopy adverse events: are we getting the full picture? Scand J Gastroenterol. 2020 Aug 2;55(8):979–87.
8. Randel KR, Schult AL, Botteri E, Hoff G, Bretthauer M, Ursin G, et al. Colorectal Cancer Screening With Repeated Fecal Immunochemical Test Versus Sigmoidoscopy: Baseline Results From a Randomized Trial. Gastroenterology. 2021 Mar;160(4):1085-1096.e5.
9. Lin JS, Perdue LA, Henrikson NB, Bean SI, Blasi PR. Screening for Colorectal Cancer: Updated Evidence Report and Systematic Review for the US Preventive Services Task Force. JAMA. 2021 May 18;325(19):1978.
10. Reumkens A, Rondagh EJA, Bakker MC, Winkens B, Masclee AAM, Sanduleanu S. Post-Colonoscopy Complications: A Systematic Review, Time Trends, and Meta-Analysis of Population-Based Studies. Am J Gastroenterol. 2016 Aug;111(8):1092–101.
11. Fitzpatrick-Lewis D, Ali MU, Warren R, Kenny M, Sherifali D, Raina P. Screening for Colorectal Cancer: A Systematic Review and Meta-Analysis. Clin Colorectal Cancer. 2016 Dec;15(4):298–313.
12. Holme Ø, Bretthauer M, Fretheim A, Odgaard-Jensen J, Hoff G. Flexible sigmoidoscopy versus faecal occult blood testing for colorectal cancer screening in asymptomatic individuals. Cochrane Colorectal Cancer Group, editor. Cochrane Database Syst Rev [Internet]. 2013 Oct 1 [cited 2022 May 18];2014(3). Available from: http://doi.wiley.com/10.1002/14651858.CD009259.pub2
13. Vermeer NCA, Snijders HS, Holman FA, Liefers GJ, Bastiaannet E, van de Velde CJH, et al. Colorectal cancer screening: Systematic review of screen-related morbidity and mortality. Cancer Treat Rev. 2017 Mar;54:87–98.
14. Niv Y, Hazazi R, Levi Z, Fraser G. Screening Colonoscopy for Colorectal Cancer in Asymptomatic People: A Meta-Analysis. Dig Dis Sci. 2008 Dec;53(12):3049–54.
