# Supplementary material for: The risk of bleeding and perforation from sigmoidoscopy or colonoscopy in colorectal cancer screening: A systematic review and meta-analyses
Source: PLoS One. 2023 Oct 31;18(10):e0292797. doi: 10.1371/journal.pone.0292797 (PMC10617695; doi:10.1371/journal.pone.0292797)
Supplement: S2 File — (PDF) [file pone.0292797.s003.pdf]

S2: All included studies that assess bleeding or perforation

Study Number	Study ID	Design	Procedure	Outcome	Analysis category
25a	Atkin 1998	RCT	FS	Bleeding	ND-NR
25b	Atkin 1998	RCT	FS	Bleeding	ND-NR
36a	Atkin 2002	RCT	FS	Bleeding	Mild-longterm
36a	Atkin 2002	RCT	FS	Bleeding	Severe-NR
63b	Forbes 2006	RCT	FS	Bleeding	ND-longterm
s34a	Holme 2014	RCT	FS	Bleeding	Severe-NR
85a	Kewenter 1996	RCT	FS	Bleeding	ND-NR
s23a	Randel 2021	RCT	FS	Bleeding	Severe-longterm
21	Jain 2002	NRS	FS	Bleeding	ND-NR
50a	Segnan 2002	RCT	FS	Bleeding	Mild-NR
22	Levin 2002	NRS	FS	Bleeding	Mild-longterm
22	Levin 2002	NRS	FS	Bleeding	ND-longterm
50a	Segnan 2002	RCT	FS	Bleeding	Severe-NR
31	Pabby 2005	NRS	FS	Bleeding	ND-NR
51a	Segnan 2005	RCT	FS	Bleeding	Mild-NR
51a	Segnan 2005	RCT	FS	Bleeding	Severe-NR
42a	Senore 2011	RCT	FS	Bleeding	ND-longterm
89	Arana-Arri 2018	NRS	TCfobt	Bleeding	Severe-longterm
s6	Dominitz 2019	RCT	TCfobt	Bleeding	Severe-longterm
s1	Benazzato 2020	NRS	TCfobt	Bleeding	Severe-longterm
44	Binefa 2013	NRS	TCfobt	Bleeding	Severe-NR
2e	Blanks 2015	NRS	TCfobt	Bleeding	ND-longterm
69	Cheng 2002	NRS	TCfobt	Bleeding	Mild-NR
76	Dancourt 2008	NRS	TCfobt	Bleeding	ND-NR
35	Denis 2007	NRS	TCfobt	Bleeding	ND-NR
34	Denis 2013	NRS	TCfobt	Bleeding	ND-longterm
s4a	Denis 2021	NRS	TCfobt	Bleeding	Mild-longterm
s4b	Denis 2021	NRS	TCfobt	Bleeding	Severe-longterm
82	Denters 2012	NRS	TCfobt	Bleeding	ND-NR

5	Denters 2013	NRS	TCfobt	Bleeding	Severe-NR
2d	Din 2015	NRS	TCfobt	Bleeding	Severe-longterm
10	Din 2017	NRS	TCfobt	Bleeding	ND-NR
2f	Ellul 2010	NRS	TCfobt	Bleeding	ND-NR
77	Faivre 2004	NRS	TCfobt	Bleeding	ND-NR
83	Garcia 2012	NRS	TCfobt	Bleeding	Severe-NR
2c	Ghanouni 2016	NRS	TCfobt	Bleeding	Mild-NR
27b	Quintero 2012	RCT	TCfobt	Bleeding	ND-NR
8	Gupta 2012	NRS	TCfobt	Bleeding	Severe-NR
s23b	Randel 2021	RCT	TCfobt	Bleeding	Severe-longterm
s24	Robertson 2019	RCT	TCfobt	Bleeding	Mild-longterm
s9	Hsu 2020	NRS	TCfobt	Bleeding	Severe-longterm
26	Hughes 2005	NRS	TCfobt	Bleeding	ND-longterm
s10	Ibánez 2018	NRS	TCfobt	Bleeding	Severe-longterm
s11	Karlijn 2021	NRS	TCfobt	Bleeding	ND-longterm
s24	Robertson 2019	RCT	TCfobt	Bleeding	Severe-longterm
4	Robinson 1999	RCT	TCfobt	Bleeding	Severe-NR
2b	Lee 2012	NRS	TCfobt	Bleeding	Mild-longterm
2b	Lee 2012	NRS	TCfobt	Bleeding	Severe-longterm
62	Marino 2012	NRS	TCfobt	Bleeding	ND-NR
s17	Meulen 2021	NRS	TCfobt	Bleeding	ND-NR
s17	Meulen 2021	NRS	TCfobt	Bleeding	ND-NR
64	Mikkelsen 2018	NRS	TCfobt	Bleeding	Severe-longterm
33	Neely 2013	NRS	TCfobt	Bleeding	ND-NR
75	Parente 2013	NRS	TCfobt	Bleeding	Severe-NR
s15	Paszat 2020	NRS	TCfobt	Bleeding	Severe-longterm
s21	Portillo 2018	NRS	TCfobt	Bleeding	Severe-longterm
s36	Quyn 2018	NRS	TCfobt	Bleeding	Severe-NR
2a	Rutter 2014	NRS	TCfobt	Bleeding	Mild-longterm
41	Saraste 2016	NRS	TCfobt	Bleeding	ND-longterm

20	Sung 2003	NRS	TCfobt	Bleeding	ND-NR
88	Tepes 2017	NRS	TCfobt	Bleeding	Severe-NR
s26	Tomaszewski 2021	NRS	TCfobt	Bleeding	Severe-longterm
s27	Vanaclocha-Espi 2018	NRS	TCfobt	Bleeding	Severe-longterm
48	Zorzi 2009	NRS	TCfobt	Bleeding	ND-NR
36b	Atkin 2002	RCT	TCfollowup	Bleeding	Mild-NR
36b	Atkin 2002	RCT	TCfollowup	Bleeding	Severe-NR
63a	Forbes 2006	RCT	TCfollowup	Bleeding	ND-longterm
46	Castro 2013	NRS	TCfollowup	Bleeding	ND-longterm
60b	Gondal 2003	RCT	TCfollowup	Bleeding	Mild-NR
19b	Hol 2010a	RCT	TCfollowup	Bleeding	Mild-longterm
s34b	Holme 2014	RCT	TCfollowup	Bleeding	Severe-NR
85b	Kewenter 1996	RCT	TCfollowup	Bleeding	Severe-longterm
70	Mandel 1993	RCT	TCfollowup	Bleeding	Severe-NR
s23c	Randel 2021	RCT	TCfollowup	Bleeding	Severe-longterm
50b	Segnan 2002	RCT	TCfollowup	Bleeding	Mild-NR
50b	Segnan 2002	RCT	TCfollowup	Bleeding	Severe-NR
s19	Naumann 2021	NRS	TCfollowup	Bleeding	ND-NR
51b	Segnan 2005	RCT	TCfollowup	Bleeding	Mild-NR
51b	Segnan 2005	RCT	TCfollowup	Bleeding	Severe-NR
s22	Rajendran 2017	NRS	TCfollowup	Bleeding	ND-NR
7b	Rutter 2012	NRS	TCfollowup	Bleeding	ND-longterm
59	Shroff 2015	NRS	TCfollowup	Bleeding	Severe-longterm
s33	Ahmed 2016	NRS	TConly	Bleeding	ND-NR
1	Bretthauer 2016	RCT	TConly	Bleeding	Severe-NR
54	Berhane 2009	NRS	TConly	Bleeding	Severe-longterm
14	Bokemeyer 2009	NRS	TConly	Bleeding	Severe-NR
s3	Causada-Calo 2020	NRS	TConly	Bleeding	ND-longterm
15	Crispin 2009	NRS	TConly	Bleeding	Severe-NR
17	Dae 2007	NRS	TConly	Bleeding	Severe-NR

3c	Ferlitsch 2011	NRS	TConly	Bleeding	ND-NR
58a	Garcia-Albeniz 2017	NRS	TConly	Bleeding	Mild-longterm
58c	Garcia-Albeniz 2017	NRS	TConly	Bleeding	Mild-longterm
58a	Garcia-Albeniz 2017	NRS	TConly	Bleeding	Severe-longterm
58c	Garcia-Albeniz 2017	NRS	TConly	Bleeding	Severe-longterm
27a	Quintero 2012	RCT	TConly	Bleeding	ND-NR
66	Huppe 2004	NRS	TConly	Bleeding	ND-NR
84	Imperiale 2000	NRS	TConly	Bleeding	Mild-NR
11	Khalid-de Bakker 2011	NRS	TConly	Bleeding	ND-longterm
12	Khalid-de Bakker 2011b	NRS	TConly	Bleeding	ND-longterm
3b	Kozbial 2015	NRS	TConly	Bleeding	ND-NR
s37	Lieberman 2000	NRS	TConly	Bleeding	Severe-longterm
32	Nelson 2002	NRS	TConly	Bleeding	Mild-longterm
32	Nelson 2002	NRS	TConly	Bleeding	Severe-longterm
s20	Pedersen 2020	NRS	TConly	Bleeding	Mild-longterm
s20	Pedersen 2020	NRS	TConly	Bleeding	Mild-longterm
s20	Pedersen 2020	NRS	TConly	Bleeding	Severe-longterm
68	Pox 2012	NRS	TConly	Bleeding	Mild-NR
68	Pox 2012	NRS	TConly	Bleeding	Severe-NR
42b	Senore 2011	RCT	TConly	Bleeding	ND-longterm
13b	Stoop 2012	RCT	TConly	Bleeding	ND-longterm
7a	Rutter 2012	NRS	TConly	Bleeding	ND-longterm
73	Schoenfeld 2005	NRS	TConly	Bleeding	Severe-NR
67	Sieg 2006	NRS	TConly	Bleeding	ND-NR
23a	Stock 2013	NRS	TConly	Bleeding	Severe-longterm
87	Strul 2006	NRS	TConly	Bleeding	ND-NR
s25	Taleban 2018	NRS	TConly	Bleeding	Severe-NR
3a	Waldmann 2016	NRS	TConly	Bleeding	ND-NR
s28	Wang 2018	NRS	TConly	Bleeding	Severe-longterm
s28	Wang 2018	NRS	TConly	Bleeding	Severe-longterm

s30	Wong 2017	NRS	TConly	Bleeding	ND-longterm
s31	Xirasagar 2020	NRS	TConly	Bleeding	Mild-NR
s32	Zwink 2017	NRS	TConly	Bleeding	ND-longterm
s32	Zwink 2017	NRS	TConly	Bleeding	ND-NR

Study Number	Study ID	Design	Procedure	Outcome	Analysis category
21	Jain 2002	NRS	FS	Perforation	ND-NR
22	Levin 2002	NRS	FS	Perforation	ND-longterm
36a	Atkin 2002	RCT	FS	Perforation	ND-NR
63b	Forbes 2006	RCT	FS	Perforation	ND-longterm
s34a	Holme 2014	RCT	FS	Perforation	ND-NR
85a	Kewenter 1996	RCT	FS	Perforation	ND-NR
s23a	Randel 2021	RCT	FS	Perforation	Mild-longterm
72a	Schoen 2012	RCT	FS	Perforation	ND-NR
50a	Segnan 2002	RCT	FS	Perforation	Mild-longterm
29b	Zubarik 2002	NRS	FS	Perforation	ND-NR
89	Arana-Arri 2018	NRS	TCfobt	Perforation	Severe-longterm
s1	Benazzato 2020	NRS	TCfobt	Perforation	Severe-longterm
44	Binefa 2013	NRS	TCfobt	Perforation	Severe-NR
69	Cheng 2002	NRS	TCfobt	Perforation	ND-NR
76	Dancourt 2008	NRS	TCfobt	Perforation	ND-NR
35	Denis 2007	NRS	TCfobt	Perforation	Severe-NR
34	Denis 2013	NRS	TCfobt	Perforation	Severe-longterm
s4a	Denis 2021	NRS	TCfobt	Perforation	ND-longterm
s4b	Denis 2021	NRS	TCfobt	Perforation	Severe-longterm
82	Denters 2012	NRS	TCfobt	Perforation	ND-NR
5	Denters 2013	NRS	TCfobt	Perforation	ND-NR
s5	Derbyshire 2018	NRS	TCfobt	Perforation	ND-longterm
2d	Din 2015	NRS	TCfobt	Perforation	Severe-longterm
10	Din 2017	NRS	TCfobt	Perforation	ND-NR
2f	Ellul 2010	NRS	TCfobt	Perforation	Severe-NR
77	Faivre 2004	NRS	TCfobt	Perforation	ND-NR
s7	Florido 2017	NRS	TCfobt	Perforation	ND-NR
83	Garcia 2012	NRS	TCfobt	Perforation	Severe-NR
8	Gupta 2012	NRS	TCfobt	Perforation	ND-NR

s9	Hsu 2020	NRS	TCfobt	Perforation	Severe-longterm
s10	Ibánez 2018	NRS	TCfobt	Perforation	Severe-longterm
s11	Karlijn 2021	NRS	TCfobt	Perforation	ND-longterm
2b	Lee 2012	NRS	TCfobt	Perforation	Severe-longterm
62	Marino 2012	NRS	TCfobt	Perforation	Severe-NR
s17	Meulen 2021	NRS	TCfobt	Perforation	ND-NR
64	Mikkelsen 2018	NRS	TCfobt	Perforation	ND-longterm
33	Neely 2013	NRS	TCfobt	Perforation	ND-NR
75	Parente 2013	NRS	TCfobt	Perforation	ND-NR
s15	Paszat 2020	NRS	TCfobt	Perforation	Severe-longterm
s21	Portillo 2018	NRS	TCfobt	Perforation	Severe-longterm
s36	Quyn 2018	NRS	TCfobt	Perforation	ND-NR
s36	Quyn 2018	NRS	TCfobt	Perforation	ND-NR
s36	Quyn 2018	NRS	TCfobt	Perforation	ND-NR
s6	Dominitz 2019	RCT	TCfobt	Perforation	ND-longterm
2a	Rutter 2014	NRS	TCfobt	Perforation	ND-longterm
41	Saraste 2016	NRS	TCfobt	Perforation	Severe-longterm
27b	Quintero 2012	RCT	TCfobt	Perforation	ND-NR
s23b	Randel 2021	RCT	TCfobt	Perforation	Mild-longterm
s24	Robertson 2019	RCT	TCfobt	Perforation	Severe-longterm
4	Robinson 1999	RCT	TCfobt	Perforation	ND-NR
81	Steele 2004	NRS	TCfobt	Perforation	ND-NR
20	Sung 2003	NRS	TCfobt	Perforation	ND-NR
88	Tepes 2017	NRS	TCfobt	Perforation	ND-NR
s26	Tomaszewski 2021	NRS	TCfobt	Perforation	Mild-longterm
s27	Vanaclocha-Espi 2018	NRS	TCfobt	Perforation	Severe-longterm
48	Zorzi 2009	NRS	TCfobt	Perforation	ND-NR
46	Castro 2013	NRS	TCfollowup	Perforation	Severe-longterm
43	Dellon 2009	NRS	TCfollowup	Perforation	ND-NR
s19	Naumann 2021	NRS	TCfollowup	Perforation	ND-NR

30	Polter 2015	NRS	TCfollowup	Perforation	ND-longterm
36b	Atkin 2002	RCT	TCfollowup	Perforation	ND-NR
63a	Forbes 2006	RCT	TCfollowup	Perforation	ND-longterm
60b	Gondal 2003	RCT	TCfollowup	Perforation	ND-NR
7b	Rutter 2012	NRS	TCfollowup	Perforation	ND-longterm
s34b	Holme 2014	RCT	TCfollowup	Perforation	ND-NR
85b	Kewenter 1996	RCT	TCfollowup	Perforation	ND-NR
70	Mandel 1993	RCT	TCfollowup	Perforation	Severe-NR
s23c	Randel 2021	RCT	TCfollowup	Perforation	Mild-longterm
72b	Schoen 2012	RCT	TCfollowup	Perforation	ND-NR
59	Shroff 2015	NRS	TCfollowup	Perforation	Severe-longterm
50b	Segnan 2002	RCT	TCfollowup	Perforation	Severe-NR
s33	Ahmed 2016	NRS	TConly	Perforation	ND-NR
54	Berhane 2009	NRS	TConly	Perforation	ND-longterm
45	Bielawska 2014	NRS	TConly	Perforation	ND-NR
14	Bokemeyer 2009	NRS	TConly	Perforation	Severe-NR
s3	Causada-Calo 2020	NRS	TConly	Perforation	Severe-longterm
80	Chiu 2013	NRS	TConly	Perforation	ND-NR
15	Crispin 2009	NRS	TConly	Perforation	Severe-NR
17	Dae 2007	NRS	TConly	Perforation	ND-NR
3c	Ferlitsch 2011	NRS	TConly	Perforation	ND-NR
58a	Garcia-Albeniz 2017	NRS	TConly	Perforation	Severe-longterm
58c	Garcia-Albeniz 2017	NRS	TConly	Perforation	Severe-longterm
52	Hamdani 2013	NRS	TConly	Perforation	ND-longterm
66	Huppe 2004	NRS	TConly	Perforation	ND-NR
84	Imperiale 2000	NRS	TConly	Perforation	ND-NR
11	Khalid-de Bakker 2011	NRS	TConly	Perforation	ND-longterm
12	Khalid-de Bakker 2011b	NRS	TConly	Perforation	ND-longterm
s16	Leventi 2021	NRS	TConly	Perforation	Severe-NR
s37	Lieberman 2000	NRS	TConly	Perforation	ND-longterm

32	Nelson 2002	NRS	TConly	Perforation	Severe-longterm
s20	Pedersen 2020	NRS	TConly	Perforation	Mild-longterm
s20	Pedersen 2020	NRS	TConly	Perforation	Mild-longterm
s20	Pedersen 2020	NRS	TConly	Perforation	Severe-longterm
68	Pox 2012	NRS	TConly	Perforation	Severe-NR
1	Bretthauer 2016	RCT	TConly	Perforation	ND-NR
7a	Rutter 2012	NRS	TConly	Perforation	ND-longterm
73	Schoenfeld 2005	NRS	TConly	Perforation	ND-NR
27a	Quintero 2012	RCT	TConly	Perforation	ND-NR
67	Sieg 2006	NRS	TConly	Perforation	Severe-NR
23a	Stock 2013	NRS	TConly	Perforation	Severe-longterm
87	Strul 2006	NRS	TConly	Perforation	ND-NR
s25	Taleban 2018	NRS	TConly	Perforation	Severe-NR
3a	Waldmann 2016	NRS	TConly	Perforation	ND-NR
s28	Wang 2018	NRS	TConly	Perforation	Severe-longterm
s31	Xirasagar 2020	NRS	TConly	Perforation	ND-NR
29a	Zubarik 2002	NRS	TConly	Perforation	ND-NR
s32	Zwink 2017	NRS	TConly	Perforation	ND-longterm
s32	Zwink 2017	NRS	TConly	Perforation	ND-NR
