# Supplementary material for: The risk of bleeding and perforation from sigmoidoscopy or colonoscopy in colorectal cancer screening: A systematic review and meta-analyses
Source: PLoS One. 2023 Oct 31;18(10):e0292797. doi: 10.1371/journal.pone.0292797 (PMC10617695; doi:10.1371/journal.pone.0292797)
Supplement: S3 File — (DOCX) [file pone.0292797.s004.docx]

S3 – General information about the systematic review

Contents

[Funding 1](#_Toc121994872)

[Patient and Public Involvement 1](#_Toc121994873)

[Availability of data and materials 2](#_Toc121994874)

[Contributor and guarantor information (CRediT taxonomy) 2](#_Toc121994875)

[Copyright/license for publication 2](#_Toc121994876)

[Competing interests: 3](#_Toc121994877)

[Transparency statement 3](#_Toc121994878)

[Ethics approval 3](#_Toc121994879)

[Patient consent for publication 3](#_Toc121994880)

[Provenance 3](#_Toc121994881)

# Funding

One reviewer, Frederik Handberg Juul Martiny, received financial support via the research grant “Sara Krabbes legat” from the Danish Society for General Practitioners (<https://www.dsam.dk/forskning/sara_krabbes_legat/>), covering expenses related to Open Access publication. The Danish Cancer Society Research Center (<https://www.cancer.dk/forskning/stoette-til-forskning/funding/>) funded one year’s salary for FHJM to conduct the systematic review, and the William Demant Foundation (<https://www.williamdemantfonden.dk/>) supported FHJM’s participation in the Preventing Overdiagnosis Conference 2017 in Quebec, Canada. The funders had no role in study design, data collection and analysis, decision to publish, or preparation of the manuscript. Frederik Handberg Juul Martiny is independent of the funding bodies.

# ****Patient and Public Involvement****

Patients/the public were not involved in the design, conduct or reporting of this review. We intend to share our results with relevant patient groups through the Danish Cancer Society.

# Availability of data and materials

The data underlying the results presented in the study are available from an Open Science Framework project via <https://osf.io/ad7nf/?view_only=20fb99934d614293900f0a789d0a350b>. The source code used in R for meta-analyses can be requested via email to the main author.

# Contributor and guarantor information (CRediT taxonomy)

*Isabella Skaarup Kindt (ISK), Frederik Handberg Juul Martiny (FHJM), Emma Grundtvig Gram (EGG), Anne Katrine Lykke Bie (AKLB), Christian Patrick Jauernik (CJ), Or Joseph Rahbek (OR), Sigrid Brisson Nielsen (SBN), Volkert Siersma (VS), Christine Winther Bang (CWB), John Brodersen (JB).*

FHJM and JB conceptualized the study and secured its funding. FHJM handled project administration, and act as guarantor for the study, working under the supervision of JB. FHJM drafted the protocol, and AKLB, CJ, OJR, SBN and JB provided comments. ISK, FHJM, EGG, AKLB, CJ, OJR and SBN assessed references for eligibility, extracted data and assessed the risk of bias in studies included for review. ISK made evidence (GRADE) profiles with feedback from FHJM (Appendix 16&17). VS contributed to the methodology of the study and supervised CWB in assisting ISK with data curation, formal analysis and visualization of data and use of software (R). ISK drafted the initial manuscript, and FHJM, EGG, AKLB, CJ, OJR, SBN, VS, CWB, and JB contributed to review and editing to reach the final manuscript. All authors had full access to all data in the study (including statistical reports and tables), taking responsibility for the integrity of the data and the accuracy of the data analysis. The corresponding author attests that all listed authors meet authorship criteria and that no others meeting the criteria have been omitted.

# Copyright/license for publication

The Corresponding Author has the right to grant on behalf of all authors and does grant on behalf of all authors, [a worldwide licence](http://www.bmj.com/sites/default/files/BMJ%20Author%20Licence%20March%202013.doc) to the Publishers and its licensees in perpetuity, in all forms, formats and media (whether known now or created in the future), to i) publish, reproduce, distribute, display and store the Contribution, ii) translate the Contribution into other languages, create adaptations, reprints, include within collections and create summaries, extracts and/or, abstracts of the Contribution, iii) create any other derivative work(s) based on the Contribution, iv) to exploit all subsidiary rights in the Contribution, v) the inclusion of electronic links from the Contribution to third party material where-ever it may be located; and, vi) licence any third party to do any or all of the above.

# Competing interests:

All authors have completed the ICMJE uniform disclosure form at [www.icmje.org/coi_disclosure.pdf](http://www.icmje.org/coi_disclosure.pdf) and declare: the first author had financial support from the Danish Society for General Practice, the Danish Cancer Society and the William Demant Foundation for the submitted work, there were no financial relationships with any organizations that might have an interest in the submitted work in the previous three years; no other relationships or activities that could appear to have influenced the submitted work.

# Transparency statement

The lead author ISK affirms that this manuscript is an honest, accurate, and transparent account of the study being reported; that no important aspects of the study have been omitted; and that any discrepancies from the study as planned (Please view PROSPERO Registration CRD42017058844) have been explained.

# Ethics approval

Not required.

# Patient consent for publication

Not required.

# Provenance

Not commissioned
