# Supplementary material for: The risk of bleeding and perforation from sigmoidoscopy or colonoscopy in colorectal cancer screening: A systematic review and meta-analyses
Source: PLoS One. 2023 Oct 31;18(10):e0292797. doi: 10.1371/journal.pone.0292797 (PMC10617695; doi:10.1371/journal.pone.0292797)
Supplement: S4 File — (PDF) [file pone.0292797.s005.pdf]

??For those soon to register with PROSPERO: records may not be submitted between the 23rd Dec - 3rd Jan whilst staff are away and the university is closed for the Christmas period. During this time you may still work on your record and save it for submission in the new year. ??

Systematic review

Fields that have an ***asterisk (*)*** *next to them means that they* ***must be answered. Word limits*** *are provided for each section. You will be unable to submit the form if the word limits are exceeded for any section. Registrant means the person filling out the form*.

This record cannot be edited because it has been marked as out of scope

1. * Review title.

Give the title of the review in English

Physical harm of screening for colorectal cancer: a systematic review

2. Original language title.

For reviews in languages other than English, give the title in the original language. This will be displayed with the English language title.

3. * Anticipated or actual start date.

Give the date the systematic review started or is expected to start.

02/01/2017

4. * Anticipated completion date.

Give the date by which the review is expected to be completed.

29/12/2017

5. * Stage of review at time of this submission.

This field uses answers to initial screening questions. It cannot be edited until after registration.

Tick the boxes to show which review tasks have been started and which have been completed.

Update this field each time any amendments are made to a published record.

The review has not yet started: No

Review stage	Started	Completed
Preliminary searches	Yes	Yes
Piloting of the study selection process	Yes	Yes
Formal screening of search results against eligibility criteria	Yes	No
Data extraction	No	No
Risk of bias (quality) assessment	No	No
Data analysis	No	No

Provide any other relevant information about the stage of the review here.

6. * Named contact.

The named contact is the guarantor for the accuracy of the information in the register record. This may be any member of the review team.

Frederik Martiny

Email salutation (e.g. "Dr Smith" or "Joanne") for correspondence:

7. * Named contact email.

Give the electronic email address of the named contact.

fhm@sund.ku.dk

8. Named contact address

Give the full institutional/organisational postal address for the named contact.

Centre of Research & Education in General Practice, Department of Public Health, University of Copenhagen

Øster Farimagsgade 5, building 24, entrance Q, ground and 1st floor

P.O.B. 2099

DK-1014 Copenhagen K

9. Named contact phone number.

Give the telephone number for the named contact, including international dialling code.

10. * Organisational affiliation of the review.

Full title of the organisational affiliations for this review and website address if available. This field may be completed as 'None' if the review is not affiliated to any organisation.

1) The Section of General Practice and the Research Unit for General Practice in Copenhagen 2) The Research Unit for General Practice in Region Zealand

Organisation web address:

1) http://publichealth.ku.dk/sections/general/ 2) http://www.regionsjaelland.dk/Kampagner/English/Hospitals/Sider/default.aspx

11. * Review team members and their organisational affiliations.

Give the personal details and the organisational affiliations of each member of the review team. Affiliation refers to groups or organisations to which review team members belong. **NOTE: email and country now MUST be entered for each person, unless you are amending a published record.**

Mr Frederik Martiny. 1) The Section of General Practice and the Research Unit for General Practice in Copenhagen 2) The Research Unit for General Practice in Region Zealand
Ms Sigrid Brisson Nielsen. 1) The Section of General Practice and the Research Unit for General Practice in Copenhagen 2) The Research Unit for General Practice in Region Zealand
Mr Or Rahbek. 1) The Section of General Practice and the Research Unit for General Practice in Copenhagen 2) The Research Unit for General Practice in Region Zealand
Mr Christian Jauernik. 1) The Section of General Practice and the Research Unit for General Practice in Copenhagen 2) The Research Unit for General Practice in Region Zealand
Ms Anne Katrine Lykke Bie. 1) The Section of General Practice and the Research Unit for General Practice in Copenhagen 2) The Research Unit for General Practice in Region Zealand
Professor John Brodersen. 1) The Section of General Practice and the Research Unit for General Practice in Copenhagen 2) The Research Unit for General Practice in Region Zealand

12. * Funding sources/sponsors.

Details of the individuals, organizations, groups, companies or other legal entities who have funded or sponsored the review.

The Danish Cancer Society Scientific Committee has granted a scholarship of 120.000 DKK to finance the salary of the main author Frederik Martiny for the year 2017. Grant number R165-A10525-16-S7. The funding source has no role in the design of this study and will not have any role during its execution, analyses, interpretation of the data, or decision to submit results.

Grant number(s)

State the funder, grant or award number and the date of award

13. * Conflicts of interest.

List actual or perceived conflicts of interest (financial or academic).

None

14. Collaborators.

Give the name and affiliation of any individuals or organisations who are working on the review but who are not listed as review team members. **NOTE: email and country must be completed for each person, unless you are amending a published record.**

15. * Review question.

State the review question(s) clearly and precisely. It may be appropriate to break very broad questions down into a series of related more specific questions. Questions may be framed or refined using PI(E)COS or similar where relevant.

What is the evidence for the physical harms of colorectal cancer screening?

Objectives: to report the number and types of studies investigating any type of physical harm of colorectal cancer screening;

to report the types of physical harms of colorectal cancer screening including the risk, magnitude and

consequences of these harms;

to assess and report whether studies have investigated if any factors modify the physical harm of colorectal cancer screening;

to assess and report the overall quality of the evidence as well as the risk of bias and the adequacy of harm measurements in studies.

16. * Searches.

State the sources that will be searched (e.g. Medline). Give the search dates, and any restrictions (e.g. language or publication date). Do NOT enter the full search strategy (it may be provided as a link or attachment below.)

The search strategy was developed and conducted in cooperation with an information specialist at the Copenhagen University Library. To maximise the amount of relevant literature retrieved a combination of index words and keywords was searched. We searched the databases PubMed, MEDLINE, Embase, CINAHL, PsycINFO and Cochrane Library. All databases were searched from the date from which the databases have literature coverage until the 12-04-2017.

We initially developed the search strategy for MEDLINE and subsequently adapted it to the other databases. We applied no restrictions concerning date, language or study design. The database search will be supplemented by a targeted grey literature search in Google Scholar. Ongoing trials will be obtained by searching WHO’s ICTRP Search Portal.

A preliminary search strategy for MEDLINE is presented in the attached document. We will report the final literature searches for all databases in the final publication. All studies identified will be compiled in the

reference programme Endnote where duplicates will be removed.

17. URL to search strategy.

Upload a file with your search strategy, or an example of a search strategy for a specific database, (including the keywords) in pdf or word format. In doing so you are consenting to the file being made publicly accessible. Or provide a URL or link to the strategy. Do NOT provide links to your search **results**.

https://www.crd.york.ac.uk/PROSPEROFILES/58844_STRATEGY_20170505.pdf

Alternatively, upload your search strategy to CRD in pdf format. Please note that by doing so you are consenting to the file being made publicly accessible.

Yes I give permission for this file to be made publicly available

18. * Condition or domain being studied.

Give a short description of the disease, condition or healthcare domain being studied in your systematic review.

Colorectal cancer screening is restricted to conventional screening methods as defined under the text box "Interventions".

The term "screening" covers all aspects of the screening cascade from invitation, primary testing to downstream diagnostic workup. Harms might occur at any step during the screening cascade [1]. We define harm as recommended in the PRISMA harms checklist [2]: Harm is the totality of adverse consequences of screening, being the direct opposite of benefits. Harm, as a term, thereby comprises both complications, safety issues, adverse events, adverse effects and side effects occurring in relation to screening for colorectal cancer.

1. Harris, R.P., et al., The harms of screening: a proposed taxonomy and application to lung cancer screening. JAMA Intern Med, 2014. 174(2): p. 281-5.

2. Zorzela, L., et al., PRISMA harms checklist: improving harms reporting in systematic reviews. BMJ, 2016. 352: p. i157.

19. * Participants/population.

Specify the participants or populations being studied in the review. The preferred format includes details of both inclusion and exclusion criteria.

Inclusion: 40 Age 80 years, at average risk of colorectal cancer and asymptomatic regarding signs of colorectal cancer.

Exclusion: People at higher than average risk of colorectal cancer, including people recruited because of personal or family history of colorectal cancer, people with known genetic susceptibility, people with earlier or current diagnosis of colorectal cancer, people with increased risk of colorectal cancer because of illness, for example inflammatory bowel disorder.

We will include studies with mixed populations if data and analyses are divided so that data regarding our target group is available and not mixed in with the data for i.e. symptomatic individuals, colorectal cancer patients etc.

20. * Intervention(s), exposure(s).

Give full and clear descriptions or definitions of the interventions or the exposures to be reviewed. The preferred format includes details of both inclusion and exclusion criteria.

Inclusion: Conventional colorectal cancer screening tools, including any type and combination of the following: Faecal occult blood test, sigmoidoscopy and colonoscopy.

Exclusion: Stool testing using in-office digital rectal exam, genetic testing, blood tests, stool DNA tests, capsule endoscopy or CT colonography.

21. * Comparator(s)/control.

Where relevant, give details of the alternatives against which the intervention/exposure will be compared (e.g. another intervention or a non-exposed control group). The preferred format includes details of both inclusion and exclusion criteria.

Included studies do not need to have a comparator/control group.

22. * Types of study to be included.

Give details of the study designs (e.g. RCT) that are eligible for inclusion in the review. The preferred format includes both inclusion and exclusion criteria. If there are no restrictions on the types of study, this should be stated.

We will include all types of study designs to promote identification of different types of harms and especially rare adverse events. The Cochrane Collaboration also recommends this practice, when reviewing studies of adverse events [3]. Both qualitative and quantitative studies will be included. Articles identified in the search strategy that do not represent original studies and hence do not report original data like journalism, editorials etc. will be excluded on title/abstract level. Likewise, systematic reviews will be excluded as they report data from other studies. We will scrutinize reference lists of systematic reviews deemed relevant to the research question to identify studies that might be missed in the database searches.

3. Loke YK, Price D, and Herxheimer A., Chapter 14: Adverse effects. In: Higgins JPT, Green S (editors). in Cochrane Handbook for Systematic Reviews of Interventions. Version 5.1.0 [updated March 2011]. The Cochrane Collaboration, 2011. Available from www.cochranehandbook.org.

23. Context.

Give summary details of the setting or other relevant characteristics, which help define the inclusion or exclusion criteria.

We will exclude studies taking place in settings that diverge from the normal screening setting to an extent that comparison with other studies is not meaningful. This exclusion will be on full text level with reasons noted.

24. * Main outcome(s).

Give the pre-specified main (most important) outcomes of the review, including details of how the outcome is defined and measured and when these measurement are made, if these are part of the review inclusion criteria.

Physical harm. We define harms as any bodily injury or condition. Physical harms are often divided in major adverse events and other adverse events. We include both types of physical harm. Major adverse events require medical assistance such as perforation or bleeding due to colonoscopy, anaesthesia complications, infections, cardiopulmonary complications etc. Other adverse events do not necessarily result in medical assistance such as discomfort, loss of sleep, physical symptoms due to diagnostic procedures, bloating, water-electrolyte disturbances etc.

Measures of effect

Please specify the effect measure(s) for you main outcome(s) e.g. relative risks, odds ratios, risk difference, and/or 'number needed to treat.

We only include harms which the screening participant experiences. We will exclude studies that report expected/potential harm to the screening participant. Such studies are excluded on abstract/title level. We accept any definition of physical harm and any measurement method and timing that does not conflict with

the above-described definition of the physical harm of screening.

25. * Additional outcome(s).

List the pre-specified additional outcomes of the review, with a similar level of detail to that required for main outcomes. Where there are no additional outcomes please state 'None' or 'Not applicable' as appropriate to the review

We will assess the adequacy of measurement of harms in studies. We will extract any data or analyses in studies investigating factors which modify the risk, magnitude or consequences of physical harm.

Measures of effect

Please specify the effect measure(s) for you additional outcome(s) e.g. relative risks, odds ratios, risk difference, and/or 'number needed to treat.

26. * Data extraction (selection and coding).

Describe how studies will be selected for inclusion. State what data will be extracted or obtained. State how this will be done and recorded.

Study selection process: according to the eligibility criteria defined above two review authors will independently assess all titles and abstracts of studies identified in the literature searches to identify potentially eligible studies. Subsequently the full-text of potentially eligible studies will be retrieved. If full text studies of potentially eligible papers are not available, authors of the studies will be contacted. All full-text studies retrieved will be independently assessed by two review authors for eligibility.

Multiple reports of the same study will be linked together when necessary. Disagreements will be resolved by discussion and if consensus is not reached, a third review author will be consulted to reach agreement. The main author will assess reference lists of included studies to identify studies relevant to the research questions that were not identified in the database search. Any study deemed relevant in the reference list assessment will also be independently assessed by a second review author to ensure objective inclusion/exclusion.

Once the total number of studies included for review is reached, we will perform a preliminary data extraction of study details. From the preliminary data extraction we will decide which types of studies to include for detailed data extraction as described below. The data extraction is divided in this two-step approach to ensure that studies are comparable so that data can be compared across studies.

The data extraction strategy has been developed using the recommendations in the PRISMA harm checklist and using a data collection template by the Cochrane Collaboration [4].

Data extraction strategy: The data extraction strategy is divided in five categories:

1. Study characteristics

2. Definition of harm(s)

3. Adequacy of the measurements of harms

4. Estimate(s) of harm(s)

5. Potential modifying factors on harms

A standardised pre-defined form will be developed and used to extract data from the included studies. As non-randomized studies are also included, we expect it will be necessary to revise the data extraction form during data extraction. All data are extracted by the main author and double-checked="checked" value="1" by co-authors. Any disagreements will be discussed until consensus, possibly involving a third review author to reach agreement.

Data extraction from studies will include:

1. Study characteristics: - Author, journal, year of publication, funding, study period, study design, information regarding population, setting and intervention.

2. Definition of harm(s): - How the harm domain(s) is defined; which types of harm constitute the harm domain(s).

3. Adequacy of the measurements of harms

- Who assesses/reports the harm?

- How is the harm measured? Are the measurements performed systematically?

- When is the harm evaluated? Prospectively or retrospectively? Measurements at different time points?

- How are the harms analysed, coded or grouped? Are any simplifications made?

3. Estimate(s) of harm(s)

- Any estimate of harm. Both qualitative and quantitative data are accepted

Potential modifying factors on harms:

- Are any factors identified as potentially modifying the magnitude, risk or consequence of the physical harm?

- How are those factors modifying the harm estimates assessed?

If any of the above data is not reported it will be designated as not reported (NR) in data extraction tables. Reviewers will not be blinded regarding authors and studies when extracting data as this has not been proved to affect data extraction [5].

We will prioritize to include comparative studies and RCTs of colorectal cancer screening. We prioritize these types of studies because they are the focus of two former Cochrane reviews regarding the benefit and harm of colorectal cancer screening [6, 7]. The United States Preventive Services Task Force also recently updated their review about colorectal cancer screening, which also assesses harms of different screening interventions to some extent [8]. In addition, a third review from the Cochrane Collaboration including harms of colorectal cancer screening is in the protocol stage. This review focuses on comparing the mortality reduction of sigmoidoscopy versus colonoscopy [9]. In summary, we have prioritized to include comparative studies to allow for comparison with existing reviews and to facilitate comparison of harm and harm assessment to benefit.

4. The Cochrane Collaboration. Good practice data extraction form. 21-09-2017]; Available from: http://epoc.cochrane.org/resources/epoc-resources-review-authors.

5. Higgins JPT Deeks JJ (editors), Chapter 7: Selecting studies and collecting data In: Higgins JPT, Green S (editors),, in Cochrane Handbook for Systematic Reviews of Interventions. Version 5.1.0 [updated March 2011]. The Cochrane Collaboration, 2011. Available from www.cochranehandbook.org.

6. Hewitson, P., et al., Cochrane systematic review of colorectal cancer screening using the fecal occult blood test (hemoccult): an update. Am J Gastroenterol, 2008. 103(6): p. 1541-9.

7. Holme, Ø., et al., Flexible sigmoidoscopy versus faecal occult blood testing for colorectal cancer screening in asymptomatic individuals. Cochrane Database of Systematic Reviews, 2013(9).

8. Bibbins-Domingo, K., et al., Screening for Colorectal Cancer: US Preventive Services Task Force Recommendation Statement. Jama, 2016. 315(23): p. 2564-2575.

9. Phillips, J., C. Ridd, and K. Thomas, Screening sigmoidoscopy and colonoscopy for reducing colorectal cancer mortality in asymptomatic persons. Cochrane Database of Systematic Reviews, 2013(9).

27. * Risk of bias (quality) assessment.

State which characteristics of the studies will be assessed and/or any formal risk of bias/quality assessment tools that will be used.

We will assess the risk of bias regarding harm estimates in studies using the Cochrane Collaborations ROBINS-I tool [10]. We will perform the risk of bias assessment on the study outcome level and not according to study design. This decision stems from the fact that the definition, measurement and reporting of harms is heterogeneous and often of poor quality in many clinical studies [3, 11]. In an attempt to account for the heterogeneity in studies regarding physical harm, we will expand the ROBINS-I tool to include other types of bias deemed relevant to the review question. The ROBINS-I tool will be altered using reporting guidelines from the Equator Network and various study quality checklists. We plan to report the altered ROBINS-I checklist in the final publication.

The risk of bias assessment will be performed independently by two review authors. Any disagreements will be discussed until consensus, possibly involving a third review author to reach agreement.

3. Loke YK, Price D, and Herxheimer A., Chapter 14: Adverse effects. In: Higgins JPT, Green S (editors). in Cochrane Handbook for Systematic Reviews of Interventions. Version 5.1.0 [updated March 2011]. The Cochrane Collaboration, 2011. Available from www.cochranehandbook.org.

10. Sterne, J.A.C., et al., ROBINS-I: a tool for assessing risk of bias in non-randomised studies of interventions. BMJ, 2016. 355.

11. Ioannidis, J.P., et al., Better reporting of harms in randomized trials: an extension of the CONSORT statement. Ann Intern Med, 2004. 141(10): p. 781-8.

28. * Strategy for data synthesis.

Describe the methods you plan to use to synthesise data. This **must not be generic text** but should be **specific to your review** and describe how the proposed approach will be applied to your data. If meta-analysis is planned, describe the models to be used, methods to explore statistical heterogeneity, and software package to be used.

For quantitative data we will calculate summed estimates of harms across studies. These pooled estimates will be presented via meta analyses using random effect models. Summed estimates will not be calculated in instances where heterogeneity between studies is deemed too great to allow for meaningful comparisons. In these instances harms will be presented by narrative syntheses.

Using the risk of bias assessment of studies, we expect to perform sensitivity analyses to test how the quality of studies might affect the aggregate harm estimates.

The harm domain “physical harm” is comprised of different types of harms, i.e. perforation, bleeding, pain etc. Wherever possible, we will present estimates of the magnitude of each type of harm alongside a GRADE evaluation of the quality of the evidence. GRADE evaluation of the evidence is performed by the main review author and double-checked="checked" value="1" by co-authors. Any disagreements will be discussed until consensus, possibly involving a third review author to reach agreement.

Zero events: Studies that do not report harms they have assessed or planned to assess are deemed selective in reporting. Reporting of zero occurrences of a type of harm is noted as a zero event in data extraction tables.

29. * Analysis of subgroups or subsets.

State any planned investigation of ‘subgroups’. Be clear and specific about which type of study or participant will be included in each group or covariate investigated. State the planned analytic approach.

Please view above.

30. * Type and method of review.

Select the type of review, review method and health area from the lists below.

Type of review

Cost effectiveness

No

Diagnostic

No

Epidemiologic

No

Individual patient data (IPD) meta-analysis

No

Intervention

No

Living systematic review

No

Meta-analysis

No

Methodology

No

Narrative synthesis

No

Network meta-analysis

No

Pre-clinical

No

Prevention

No

Prognostic

No

Prospective meta-analysis (PMA)

No

Review of reviews

No

Service delivery

No

Synthesis of qualitative studies

No

Systematic review

Yes

Other

No

Health area of the review

Alcohol/substance misuse/abuse

No

Blood and immune system

No

Cancer

Yes

Cardiovascular

No

Care of the elderly

No

Child health

No

Complementary therapies

No

COVID-19

No

Crime and justice

No

Dental

No

Digestive system

No

Ear, nose and throat

No

Education

No

Endocrine and metabolic disorders

No

Eye disorders

No

General interest

No

Genetics

No

Health inequalities/health equity

No

Infections and infestations

No

International development

No

Mental health and behavioural conditions

No

Musculoskeletal

No

Neurological

No

Nursing

No

Obstetrics and gynaecology

No

Oral health

No

Palliative care

No

Perioperative care

No

Physiotherapy

No

Pregnancy and childbirth

No

Public health (including social determinants of health)

No

Rehabilitation

No

Respiratory disorders

No

Service delivery

No

Skin disorders

No

Social care

No

Surgery

No

Tropical Medicine

No

Urological

No

Wounds, injuries and accidents

No

Violence and abuse

No

31. Language.

Select each language individually to add it to the list below, use the bin icon to remove any added in error.
English

There is an English language summary.

32. * Country.

Select the country in which the review is being carried out. For multi-national collaborations select all the countries involved.

Denmark

33. Other registration details.

Name any other organisation where the systematic review title or protocol is registered (e.g. Campbell, or The Joanna Briggs Institute) together with any unique identification number assigned by them. If extracted data will be stored and made available through a repository such as the Systematic Review Data Repository (SRDR), details and a link should be included here. If none, leave blank.

34. Reference and/or URL for published protocol.

If the protocol for this review is published provide details (authors, title and journal details, preferably in Vancouver format)

Add web link to the published protocol.

Or, upload your published protocol here in pdf format. Note that the upload will be publicly accessible.

Yes I give permission for this file to be made publicly available

Please note that the information required in the PROSPERO registration form must be completed in full even if access to a protocol is given.

35. Dissemination plans.

Do you intend to publish the review on completion?

Yes

Give brief details of plans for communicating review findings.?

We aim to disseminate our research findings by submitting a paper to a leading journal in the research field.

Furthermore, we plan to present both preliminary research findings and results at various conferences.

36. Keywords.

Give words or phrases that best describe the review. Separate keywords with a semicolon or new line. Keywords help PROSPERO users find your review (keywords do not appear in the public record but are included in searches). Be as specific and precise as possible. Avoid acronyms and abbreviations unless these are in wide use.

Screening

Cancer screening

Colorectal cancer

Physical harm

Harms

Adverse events

Systematic review

Quantitative research

37. Details of any existing review of the same topic by the same authors.

If you are registering an update of an existing review give details of the earlier versions and include a full bibliographic reference, if available.

38. * Current review status.

Update review status when the review is completed and when it is published.New registrations must be ongoing so this field is not editable for initial submission.

Please provide anticipated publication date

Review_Ongoing

39. Any additional information.

Provide any other information relevant to the registration of this review.

40. Details of final report/publication(s) or preprints if available.

Leave empty until publication details are available OR you have a link to a preprint (NOTE: this field is not editable for initial submission). List authors, title and journal details preferably in Vancouver format.

Give the link to the published review or preprint.
