# Supplementary material for: The risk of bleeding and perforation from sigmoidoscopy or colonoscopy in colorectal cancer screening: A systematic review and meta-analyses
Source: PLoS One. 2023 Oct 31;18(10):e0292797. doi: 10.1371/journal.pone.0292797 (PMC10617695; doi:10.1371/journal.pone.0292797)
Supplement: S1 Fig — (PDF) [file pone.0292797.s006.pdf]

S2 – Figures

Meta-analyses of all types of bleeding events

- Once-only colonoscopy
- Colonoscopy following FIT
- Sigmoidoscopy
- Colonoscopy following any screening tests

Once-only colonoscopy longterm

Study	Events	People	Weight	Point estimate per 100.000 people, 95% CI
Khalid-de Bakker 2011 (11	0	214	4.11 %	0 [0 , 1709]
Khalid-de Bakker 2011 (11	0	447	4.11 %	0 [0 , 822]
Stoop 2012	2	1276	5.81 %	157 [19 , 565]
Stock 2013	4	8658	6.25 %	46 [13 , 118]
Berhane 2009	5	11808	6.34 %	42 [14 , 99]
Lieberman 2000	6	3121	6.41 %	192 [71 , 418]
Nelson 2002	13	3196	6.59 %	407 [217 , 695]
Senore 2011	15	1198	6.61 %	1252 [702 , 2057]
Zwink 2017	35	5252	6.69 %	666 [465 , 926]
Garcia-Albeniz 2017 (58c)	62	31193	6.71 %	199 [152 , 255]
Garcia-Albeniz 2017 (58a)	75	46872	6.72 %	160 [126 , 201]
Causada-Calo 2020	76	30443	6.72 %	250 [197 , 312]
Rutter 2012	103	38472	6.73 %	268 [219 , 325]
Pedersen 2020	151	11163	6.73 %	1353 [1147 , 1585]
Wang 2018	405	645095	6.74 %	63 [57 , 69]
Wong 2017	500	833	6.74 %	60024 [56607 , 63370]
1) Subtotal (not critical)	827	785654	67.78 %	202 [106 , 386]
2) Subtotal (critical)	625	53587	32.23 %	648 [69 , 6119]
3) Total	1452	839241	100 %	268 [106 , 676]

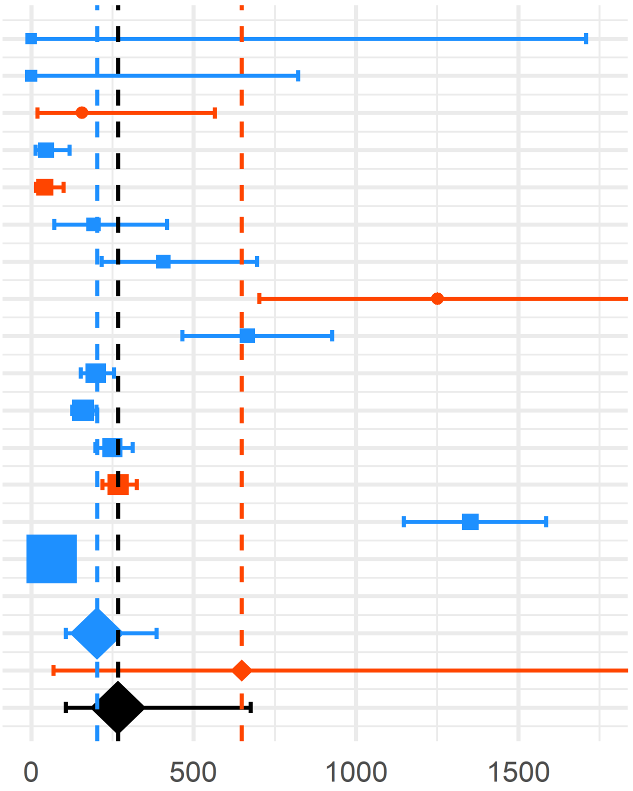

Heterogeneity:

1) $\tau^2 = 0.97$, $I^2 = 99.13$ %, $\chi^2 = 790.39$ (df = 10 , p-value = 0)

2) $\tau^2 = 6.45$, $I^2 = 99.86$ %, $\chi^2 = 3598.67$ (df = 4 , p-value = 0)

3) $\tau^2 = 3.3$, $I^2 = 99.88$ %, $\chi^2 = 5952.47$ (df = 15 , p-value = 0)

Once-only colonoscopy shortterm/not reported

Study	Events	People	Weight	Point estimate per 100.000 people, 95% CI
Dae 2007	0	4491	0.61 %	0 [0 , 82]
Schoeneld 2005	0	1483	0.61 %	0 [0 , 248]
Strul 2006	0	1177	0.61 %	0 [0 , 313]
Ahmed 2016	1	1049	1.14 %	95 [2 , 530]
Taleban 2018	1	99	1.14 %	1010 [26 , 5500]
Imperiale 2000	3	1994	2.76 %	150 [31 , 439]
Huppe 2004	4	1117	3.36 %	358 [98 , 914]
Quintero 2012	12	5059	5.9 %	237 [123 , 414]
Bretthauer 2016	18	11912	6.75 %	151 [90 , 239]
Zwink 2017	19	5252	6.86 %	362 [218 , 564]
Xirasagar 2020	24	25008	7.28 %	96 [61 , 143]
Ferlitsch 2011	54	44350	8.36 %	122 [91 , 159]
Kozbial 2015	71	59901	8.61 %	119 [93 , 149]
Crispin 2009	134	55993	9 %	239 [201 , 283]
Walsmann 2016	165	159246	9.09 %	104 [88 , 121]
Sieg 2006	183	108133	9.13 %	169 [146 , 196]
Bokemeyer 2009	442	264602	9.34 %	167 [152 , 183]
Pox 2012	2996	2773783	9.47 %	108 [104 , 112]
1) Subtotal (not critical)	724	511518	49.96 %	145 [106 , 197]
2) Subtotal (critical)	3403	3013131	50.06 %	130 [84 , 202]
3) Total	4127	3524649	100 %	140 [111 , 177]

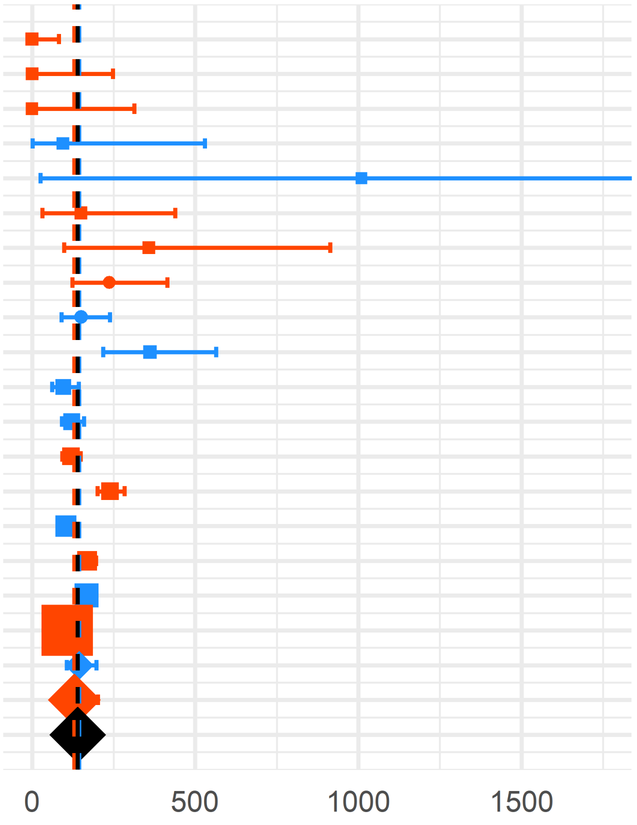

Heterogeneity:

1) $\tau^2 = 0.12$, $I^2 = 86.79$ %, $\chi^2 = 49.77$ (df = 7 , p-value = 0)

2) $\tau^2 = 0.25$, $I^2 = 92.67$ %, $\chi^2 = 117.59$ (df = 9 , p-value = 0)

3) $\tau^2 = 0.15$, $I^2 = 91.94$ %, $\chi^2 = 196.31$ (df = 17 , p-value = 0)

Colonoscopy following FIT longterm

Study	Events	People	Weight	Point estimate per 100.000 people, 95% CI
Hughes 2005	2	92	2.73 %	2174 [264 , 7633]
Ibanez 2018	10	7467	4.14 %	134 [64 , 246]
Din 2015	12	38242	4.23 %	31 [16 , 55]
Saraste 2016	18	2984	4.4 %	603 [358 , 952]
Dominitz 2019	31	14661	4.54 %	211 [144 , 300]
Denis 2021 (s4b)	43	9061	4.6 %	475 [344 , 639]
Randel 2021	44	6945	4.6 %	634 [461 , 850]
Mikkelsen 2018	60	14671	4.65 %	409 [312 , 526]
Vanaclocha-Espi 2018	63	41204	4.65 %	153 [118 , 196]
Denis 2021 (s4a)	69	17152	4.66 %	402 [313 , 509]
Denis 2013	97	10024	4.69 %	968 [785 , 1179]
Lee 2012	155	30829	4.72 %	503 [427 , 588]
Tomaszewski 2021	203	78831	4.73 %	258 [223 , 295]
Paszat 2020	219	51310	4.73 %	427 [372 , 487]
Robertson 2019	231	17485	4.73 %	1321 [1157 , 1502]
Arana-Arri 2018	245	36346	4.73 %	674 [593 , 764]
Benazzato 2020	281	99674	4.74 %	282 [250 , 317]
Portillo 2018	330	48759	4.74 %	677 [606 , 754]
Karlijn 2021	401	47270	4.74 %	848 [768 , 935]
Blanks 2015	777	112024	4.75 %	694 [646 , 744]
Rutter 2014	847	112024	4.75 %	756 [706 , 809]
Hsu 2020	2125	214955	4.76 %	989 [947 , 1031]
1) Subtotal (not critical)	6018	975664	95.28 %	436 [304 , 624]
2) Subtotal (critical)	245	36346	4.73 %	674 [593 , 764]
3) Total	6263	1012010	100 %	445 [315 , 627]

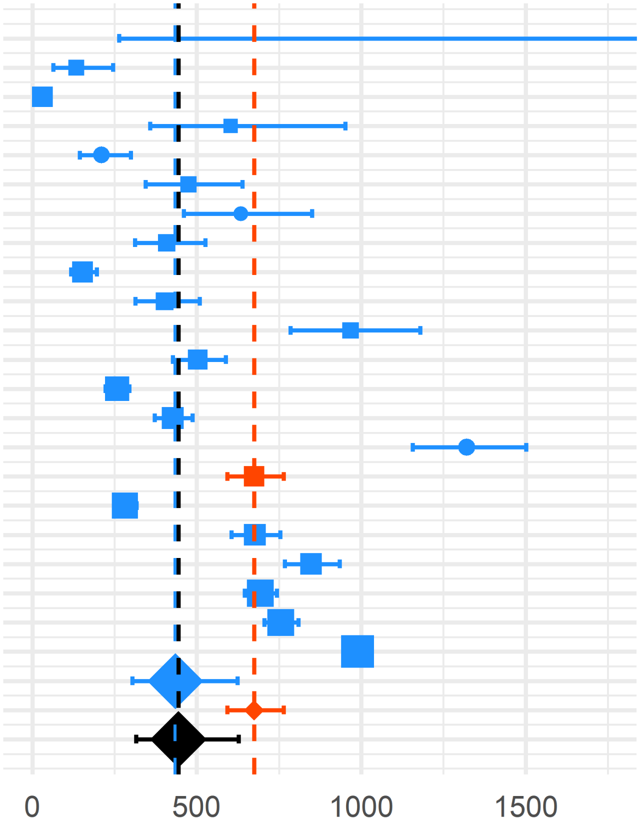

Heterogeneity:

1) $\tau^2 = 0.67$, $I^2 = 98.41$ %, $\chi^2 = 1800.4$ (df = 20 , p-value = 0)

2) τ^2 = . , I^2 = . χ^2 = .

3) $\tau^2 = 0.64$, $I^2 = 98.34$ %, $\chi^2 = 1802.21$ (df = 21 , p-value = 0)

Colonoscopy following FIT shortterm/not reported

Study	Events	People	Weight	Point estimate per 100.000 people, 95% CI
Dancourt 2008	0	1205	2.64 %	0 [0 , 306]
Faivre 2004	0	1339	2.64 %	0 [0 , 275]
Denters 2013	1	273	3.54 %	366 [9 , 2024]
Din 2017	1	780	3.54 %	128 [3 , 712]
Robinson 1999	1	1249	3.54 %	80 [2 , 445]
Sung 2003	1	476	3.54 %	210 [5 , 1165]
Ellul 2010	3	1039	4.58 %	289 [60 , 841]
Denis 2007	4	2310	4.75 %	173 [47 , 443]
Tepes 2017	4	13674	4.75 %	29 [8 , 75]
Cheng 2002	5	6266	4.86 %	80 [26 , 186]
Denters 2012	5	491	4.86 %	1018 [331 , 2360]
Garcia 2012	6	989	4.94 %	607 [223 , 1316]
Neely 2012	6	176	4.94 %	3409 [1261 , 7272]
Gupta 2012	8	1057	5.04 %	757 [327 , 1486]
Quintero 2012	8	663	5.04 %	1207 [522 , 2364]
Marino 2012	11	1000	5.12 %	1100 [550 , 1960]
Meulen 2021	13	225	5.16 %	5778 [3112 , 9678]
Binefa 2013	15	1691	5.18 %	887 [497 , 1459]
Parente 2013	29	3698	5.27 %	784 [526 , 1124]
Zorzi 2009	190	29276	5.35 %	649 [560 , 748]
Quyn 2018	213	53332	5.35 %	399 [348 , 457]
Ghanouni 2016	3865	50858	5.36 %	7600 [7371 , 7833]
1) Subtotal (not critical)	3874	52391	13.94 %	1156 [202 , 6629]
2) Subtotal (critical)	515	119676	86.05 %	340 [338 , 342]
3) Total	4389	172067	100 %	399 [203 , 782]

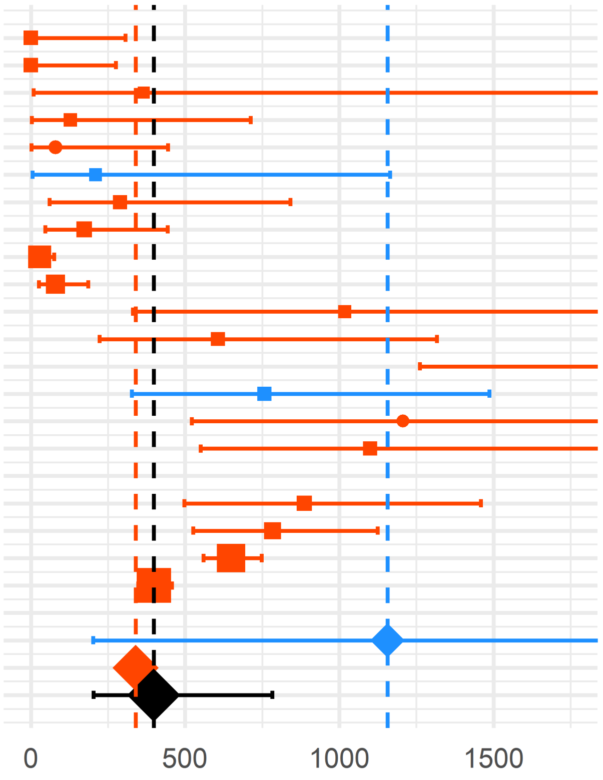

Heterogeneity:

1) $\tau^2 = 2.15$, $I^2 = 96.38$ %, $\chi^2 = 167.99$ (df = 2 , p-value = 0)

2) $\tau^2 = 2.08$, $I^2 = 91.16$ %, $\chi^2 = 268.95$ (df = 18 , p-value = 0)

3) $\tau^2 = 2.32$, $I^2 = 99.37$ %, $\chi^2 = 6850.43$ (df = 21 , p-value = 0)

Sigmoidoscopy longterm

Study	Events	People	Weight	Point estimate per 100.000 people, 95% CI
Forbes 2006	0	39	15.47 %	0 [0 , 9025]
Randel 2021	3	36065	20.31 %	8 [2 , 24]
Levin 2002	11	107704	21.28 %	10 [5 , 18]
Senore 2011	12	1502	21.32 %	799 [413 , 1391]
Aktin 2002)	77	40674	21.62 %	189 [149 , 237]
1) Subtotal (not critical)	91	184482	78.68 %	25 [5 , 134]
2) Subtotal (critical)	12	1502	21.32 %	799 [413 , 1391]
3) Total	103	185984	100 %	56 [9 , 362]

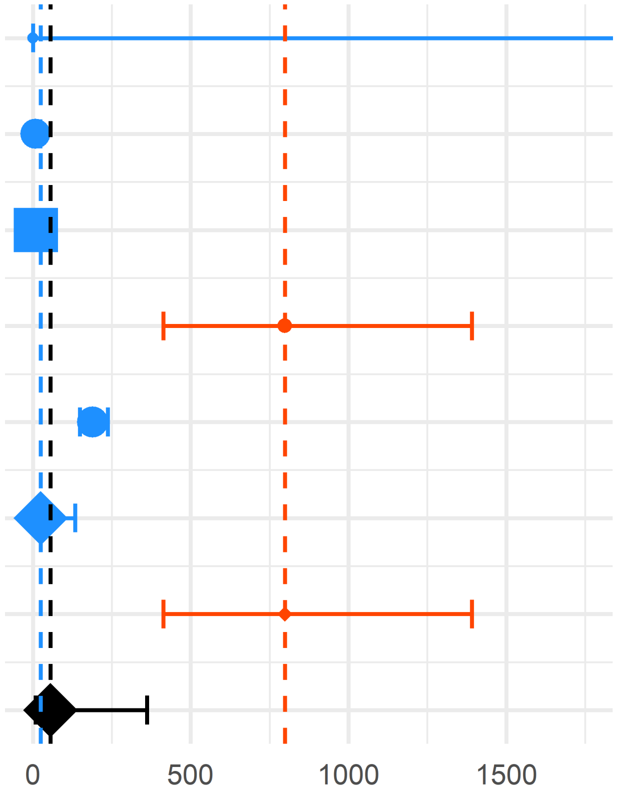

Heterogeneity:

1) $\tau^2 = 2.09$, $I^2 = 97.12$ %, $\chi^2 = 161.79$ (df = 3 , p-value = 0)

2) τ^2 = . , I^2 = . χ^2 = .

3) $\tau^2 = 3.6$, $I^2 = 97.19$ %, $\chi^2 = 204.78$ (df = 4 , p-value = 0)

Sigmoidoscopy shortterm/not reported

Study	Events	People	Weight	Point estimate per 100.000 people, 95% CI
Holme 2014	0	12955	8.72 %	0 [0 , 28]
Jain 2002	0	4229	8.72 %	0 [0 , 87]
Kewenter 1996	0	2108	8.72 %	0 [0 , 175]
Pabby 2005	3	6968	11.82 %	43 [9 , 126]
Segnan 2005	5	4466	12.16 %	112 [36 , 261]
Aktin 1998 (25b)	7	536	12.32 %	1306 [527 , 2672]
Segnan 2002	10	9911	12.43 %	101 [48 , 185]
Aktin 2002	12	40674	12.48 %	30 [15 , 52]
Aktin 1998 (25a)	33	770	12.63 %	4286 [2968 , 5966]
1) Subtotal (not critical)	15	47642	24.3 %	31 [19 , 52]
2) Subtotal (critical)	55	34975	75.7 %	35 [1 , 834]
3) Total	70	82617	100 %	46 [6 , 336]

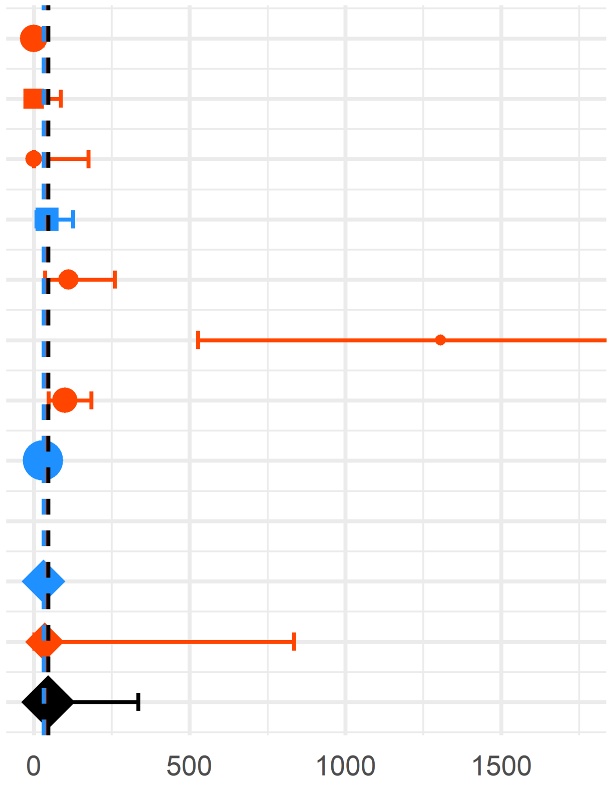

Heterogeneity:

1) $\tau^2 = 0$, $I^2 = 0$ %, $\chi^2 = 0.32$ (df = 1 , p-value = 0.5733)

2) $\tau^2 = 11.17$, $I^2 = 95.78$ %, $\chi^2 = 235.5$ (df = 6 , p-value = 0)

3) $\tau^2 = 6.97$, $I^2 = 97.4$ %, $\chi^2 = 274.15$ (df = 8 , p-value = 0)

Colonoscopy following any screening tests longterm

Study	Events	People	Weight	Point estimate per 100.000 people, 95% CI
Forbes 2006	0	112	8.51 %	0 [0 , 3240]
Castro 2013	1	3215	11.76 %	31 [1 , 173]
Kewenter 1996	1	185	11.76 %	541 [14 , 2975]
Shroff 2015	2	6152	14.54 %	33 [4 , 117]
Hol 2010	4	332	16.48 %	1205 [329 , 3056]
Rutter 2012	19	4984	18.42 %	381 [230 , 595]
Randel 2021	23	3297	18.52 %	698 [443 , 1045]
1) Subtotal (not critical)	23	3409	27.03 %	675 [448 , 1015]
2) Subtotal (critical)	27	14868	72.96 %	168 [44 , 649]
3) Total	50	18277	100 %	205 [65 , 644]

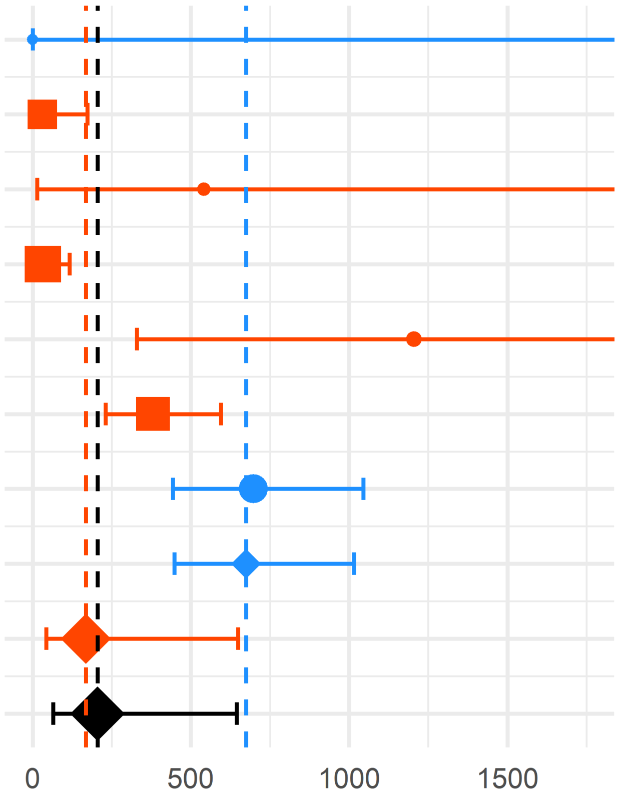

Heterogeneity:

1) $\tau^2 = 0$, $I^2 = 0$ %, $\chi^2 = 1.54$ (df = 1 , p-value = 0.2151)

2) $\tau^2 = 1.89$, $I^2 = 83.05$ %, $\chi^2 = 35.09$ (df = 4 , p-value = 0)

3) $\tau^2 = 1.7$, $I^2 = 79.74$ %, $\chi^2 = 56.02$ (df = 6 , p-value = 0)

Colonoscopy following any screening tests shortterm/not reported

Study	Events	People	Weight	Point estimate per 100.000 people, 95% CI
Gondal 2003	4	2524	11.38 %	158 [43 , 405]
Holme 2014	4	2746	11.38 %	146 [40 , 373]
Segnan 2002	8	775	12.47 %	1032 [447 , 2024]
Segnan 2005	9	332	12.61 %	2711 [1247 , 5083]
Mandel 1993	11	11941	12.81 %	92 [46 , 165]
Naumann 2021	15	5219	13.06 %	287 [161 , 474]
Aktin 2002	16	2051	13.11 %	780 [447 , 1264]
Rajemdran 2017	18	4953	13.18 %	363 [216 , 574]
1) Subtotal (not critical)	16	2051	13.11 %	780 [447 , 1264]
2) Subtotal (critical)	69	28490	86.89 %	333 [147 , 754]
3) Total	85	30541	100 %	372 [178 , 774]

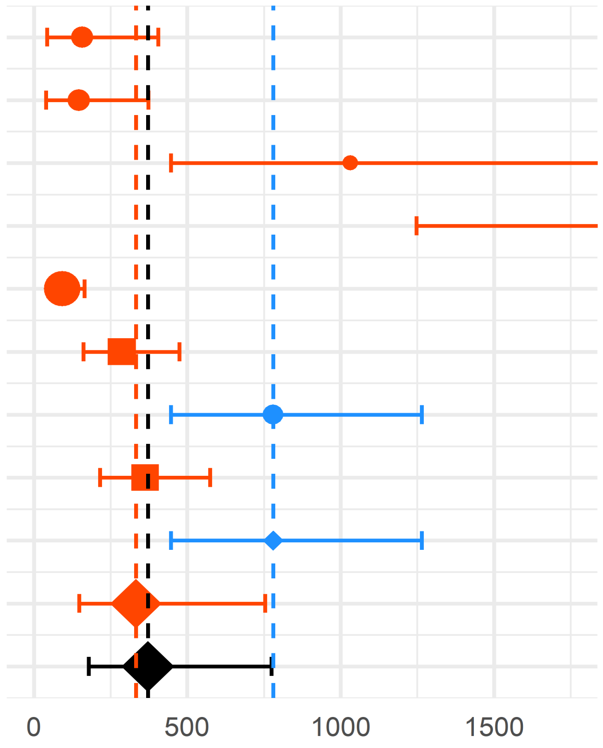

Heterogeneity:

1) τ^2 = . , I^2 = . , χ^2 = .

2) τ^2 = 1.1 , I^2 = 91.75 %, χ^2 = 57.69 (df = 6 , p-value = 0)

3) τ^2 = 1.01 , I^2 = 91.25 %, χ^2 = 71.49 (df = 7 , p-value = 0)

Meta-analyses of all types of perforations

- Once-only colonoscopy
- Colonoscopy following FIT
- Sigmoidoscopy
- Colonoscopy following any screening tests

Once-only colonoscopy longterm

Study	Events	People	Weight	Point estimate per 100.000 people, 95% CI
Khalid-de Bakker 2011 (11	0	214	3.12 %	0 [0 , 1709]
Khalid-de Bakker 2011 (12	0	447	3.12 %	0 [0 , 822]
Lieberman 2000	0	3121	3.12 %	0 [0 , 118]
Nelson 2002	0	3196	3.12 %	0 [0 , 115]
Berhane 2009	2	11808	6.43 %	17 [2 , 61]
Hamdani 2013	3	29396	7.29 %	10 [2 , 30]
Stock 2013	7	8658	8.6 %	81 [33 , 167]
Zwink 2017	8	5252	8.75 %	152 [66 , 300]
Garcia-Albaniz 2017 (58c)	11	31193	9.04 %	35 [18 , 63]
Causada-Calo 2020	12	30443	9.11 %	39 [20 , 69]
Rutter 2012	15	38472	9.26 %	39 [22 , 64]
Garcia-Albeniz 2017 (58a)	20	46872	9.42 %	43 [26 , 66]
Pedersem 2020	48	11163	9.72 %	430 [317 , 570]
Wang 2018	186	645095	9.88 %	29 [25 , 33]
1) Subtotal (not critical)	292	785654	77 %	50 [49 , 50]
2) Subtotal (critical)	20	79676	22.98 %	21 [10 , 46]
3) Total	312	865330	100 %	39 [38 , 39]

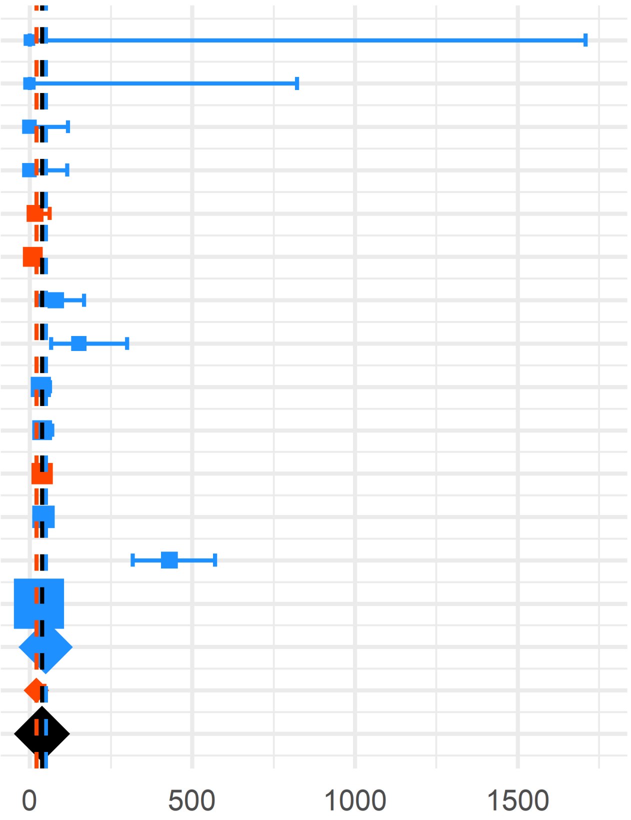

Heterogeneity:

1) $\tau^2 = 0.97$, $I^2 = 96.58$ %, $\chi^2 = 179.82$ (df = 10 , p-value = 0)

2) $\tau^2 = 0.2$, $I^2 = 61.26$ %, $\chi^2 = 6.24$ (df = 2 , p-value = 0.0442)

3) $\tau^2 = 1.02$, $I^2 = 95.71$ %, $\chi^2 = 189.3$ (df = 13 , p-value = 0)

Once-only colonoscopy shortterm/not reported

Study	Events	People	Weight	Point estimate per 100.000 people, 95% CI
Chiu 2013	0	18296	3.05 %	0 [0 , 20]
Dae 2007	0	4491	3.05 %	0 [0 , 82]
Schoenfeld 2005	0	1483	3.05 %	0 [0 , 248]
Strul 2006	0	1177	3.05 %	0 [0 , 313]
Zubarik 2002	0	281	3.05 %	0 [0 , 1304]
Ahmed 2016	0	1049	3.05 %	0 [0 , 351]
Taleban 2018	1	99	4.15 %	1010 [26 , 5500]
Huppe 2004	1	1117	4.15 %	90 [2 , 498]
Imperiale 2000	1	1994	4.15 %	50 [1 , 279]
Bretthauer 2016	1	11912	4.15 %	8 [0 , 47]
Quintero 2012	1	5059	4.15 %	20 [1 , 110]
Leventi 2021	2	2298	5.06 %	87 [11 , 314]
Ferlitsch 2011	3	44350	5.46 %	7 [1 , 20]
Zwink 2017	4	5252	5.68 %	76 [21 , 195]
Waldmann 2016	20	159246	6.31 %	13 [8 , 19]
Crispin 2009	22	55993	6.32 %	39 [25 , 59]
Sieg 2006	23	108133	6.33 %	21 [13 , 32]
Bokemeyer 2009	55	264602	6.42 %	21 [16 , 27]
Bielawska 2014	62	535286	6.42 %	12 [9 , 15]
Xirasagar 2020	260	25008	6.47 %	1040 [918 , 1173]
Pox 2012	439	2773783	6.47 %	16 [14 , 17]
1) Subtotal (not critical)	406	1046804	48.11 %	31 [9 , 109]
2) Subtotal (critical)	489	2974105	51.88 %	20 [13 , 32]
3) Total	895	4020909	100 %	22 [10 , 46]

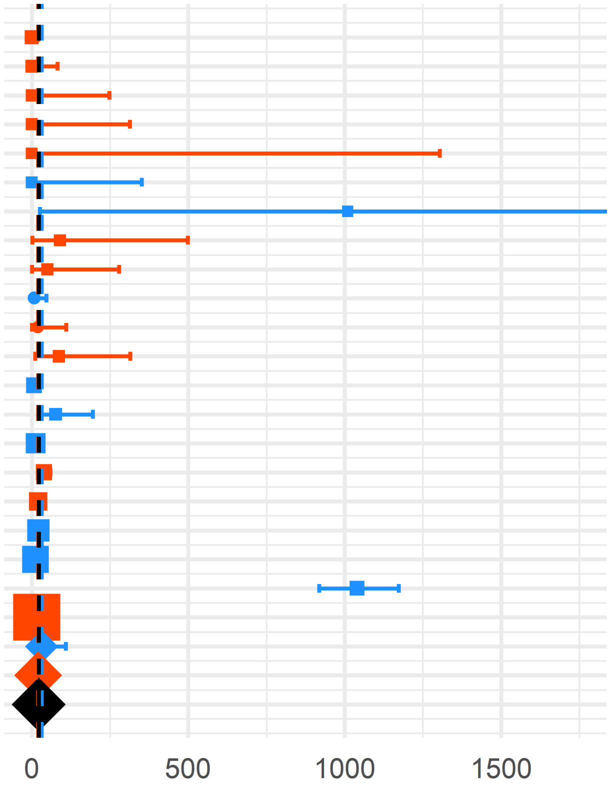

Heterogeneity:

1) $\tau^2 = 3.08$, $I^2 = 99.53$ %, $\chi^2 = 1444.89$ (df = 8 , p-value = 0)

2) $\tau^2 = 0.18$, $I^2 = 60.88$ %, $\chi^2 = 29.35$ (df = 11 , p-value = 0.00

3) $\tau^2 = 1.98$, $I^2 = 99.38$ %, $\chi^2 = 1628.91$ (df = 20 , p-value = 0)

Colonoscopy following FIT longterm

Study	Events	People	Weight	Point estimate per 100.000 people, 95% CI
Randel 2021	3	6945	3.14 %	43 [9 , 126]
Saraste 2016	3	2984	3.14 %	101 [21 , 294]
Dominitz 2019	5	14661	3.78 %	34 [11 , 80]
Robertson 2019	5	17485	3.78 %	29 [9 , 67]
Denis 2013	10	10024	4.45 %	100 [48 , 183]
Ibanez 2018	13	47270	4.65 %	28 [15 , 47]
Mikkelsen 2018	15	14671	4.74 %	102 [57 , 169]
Din 2015	16	38242	4.77 %	42 [24 , 68]
Denis 2021 (s4b)	18	9061	4.84 %	199 [118 , 314]
Denis 2021 (s4a)	20	17152	4.89 %	117 [71 , 180]
Karlijn 2021	32	47270	5.08 %	68 [46 , 96]
Lee 2012	35	30829	5.11 %	114 [79 , 158]
Tomaszewski 2021	48	78831	5.19 %	61 [45 , 81]
Paszat 2020	57	121626	5.22 %	47 [35 , 61]
Benazzato 2020	65	99674	5.25 %	65 [50 , 83]
Rutter 2014	82	112024	5.28 %	73 [58 , 91]
Vanaclovha-Espi 2018	98	41204	5.31 %	238 [193 , 290]
Arana-Arri 2018	106	36346	5.32 %	292 [239 , 353]
Derbyshire 2018	147	222488	5.35 %	66 [56 , 78]
Portillo 2018	148	48759	5.35 %	304 [257 , 356]
Hsu 2020	277	214955	5.38 %	129 [114 , 145]
1) Subtotal (not critical)	1097	1196155	94.7 %	80 [59 , 107]
2) Subtotal (critical)	106	36346	5.32 %	292 [239 , 353]
3) Total	1203	1232501	100 %	85 [62 , 115]

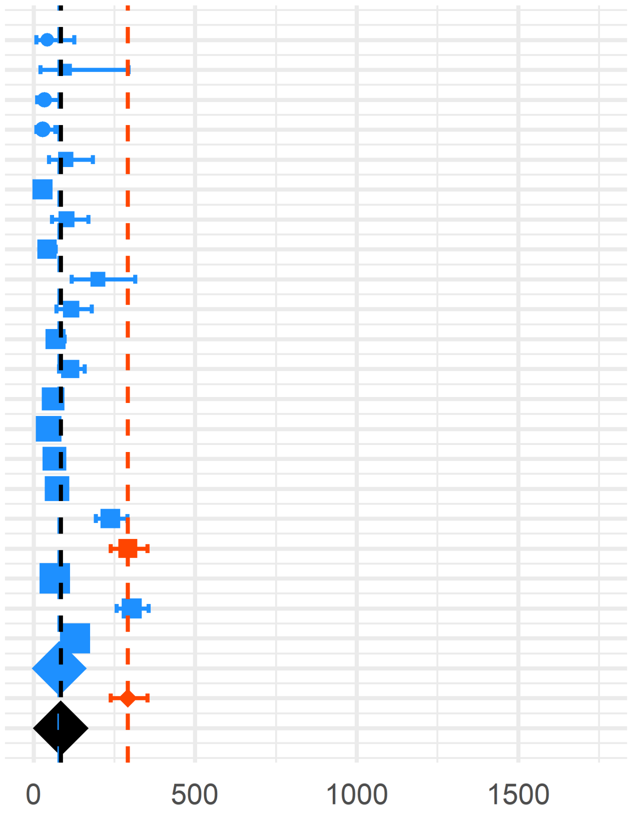

Heterogeneity:

1) $\tau^2 = 0.39$, $I^2 = 95.46$ %, $\chi^2 = 391.86$ (df = 19 , p-value = 0)

2) τ^2 = . , I^2 = . χ^2 = .

3) $\tau^2 = 0.45$, $I^2 = 96.08$ %, $\chi^2 = 487.21$ (df = 20 , p-value = 0)

Colonoscopy following FIT shortterm/not reported

Study	Events	People	Weight	Point estimate per 100.000 people, 95% CI
Dancourt 2008	0	1205	1.43 %	0 [0 , 306]
Denters 2013	0	273	1.43 %	0 [0 , 1342]
Ellul 2010	0	1039	1.43 %	0 [0 , 354]
Faivre 2004	0	1339	1.43 %	0 [0 , 275]
Meulen 2021	0	225	1.43 %	0 [0 , 1626]
Neely 2013	0	176	1.43 %	0 [0 , 2074]
Quintero 2012	0	663	1.43 %	0 [0 , 555]
Sung 2003	0	476	1.43 %	0 [0 , 772]
Denters 2012	1	491	2.61 %	204 [5 , 1129]
Gupta 2012	1	1057	2.61 %	95 [2 , 526]
Marino 2012	1	1000	2.61 %	100 [3 , 556]
Cheng 2002	2	6266	4.44 %	32 [4 , 115]
Din 2017	2	780	4.44 %	256 [31 , 923]
Florido 2017	2	842	4.44 %	238 [29 , 855]
Parente 2013	2	3698	4.44 %	54 [7 , 195]
Steele 2004	2	4116	4.44 %	49 [6 , 175]
Denis 2007	2	2310	4.44 %	87 [10 , 312]
Binefa 2013	3	1691	5.79 %	177 [37 , 518]
Garcia 2012	3	989	5.79 %	303 [63 , 884]
Robinson 1999	5	1249	7.65 %	400 [130 , 932]
Tepes 2017	7	13674	8.87 %	51 [21 , 105]
Zorzi 2009	27	29276	12.6 %	92 [61 , 134]
Quyn 2018	45	53332	13.39 %	84 [62 , 113]
1) Subtotal (not critical)	8	15207	12.91 %	53 [26 , 105]
2) Subtotal (critical)	97	110960	87.09 %	87 [72 , 107]
3) Total	105	126167	100 %	83 [69 , 101]

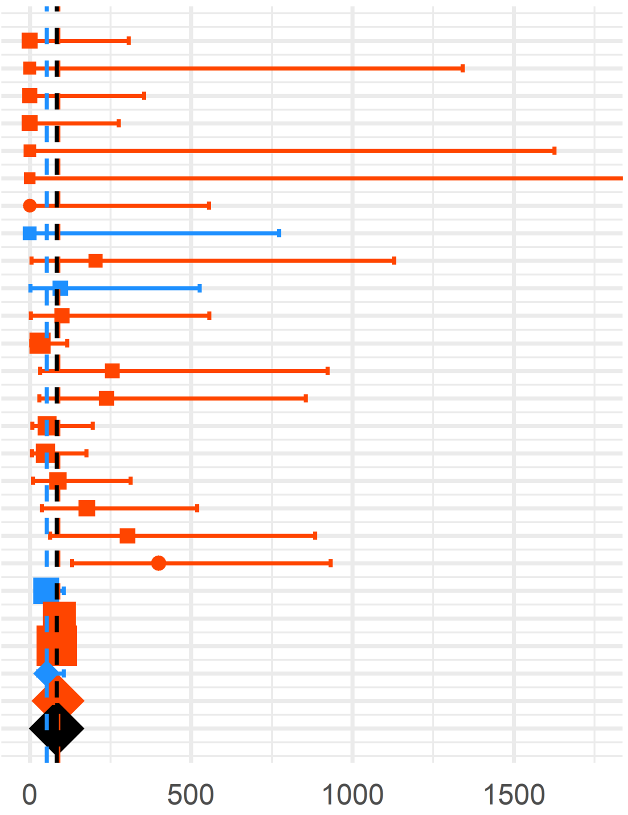

Heterogeneity:

1) $\tau^2 = 0$, $I^2 = 0$ %, $\chi^2 = 0.79$ (df = 2 , p-value = 0.673)

2) $\tau^2 = 0$, $I^2 = 20.78$ %, $\chi^2 = 28.55$ (df = 19 , p-value = 0.0733)

3) $\tau^2 = 0$, $I^2 = 18.59$ %, $\chi^2 = 31.55$ (df = 22 , p-value = 0.0854)

Sigmoidoscopy longterm

Study	Events	People	Weight	Point estimate per 100.000 people, 95% CI
Forbes 2006	0	39	21.51 %	0 [0 , 9025]
Segnan 2002	1	9911	24.64 %	10 [0 , 56]
Levin 2002	2	107704	26.57 %	2 [0 , 7]
Randel 2021	3	36065	27.28 %	8 [2 , 24]
1) Subtotal (not critical)	5	143808	75.36 %	4 [1 , 10]
2) Subtotal (critical)	1	9911	24.64 %	10 [0 , 56]
3) Total	6	153719	100 %	4 [2 , 11]

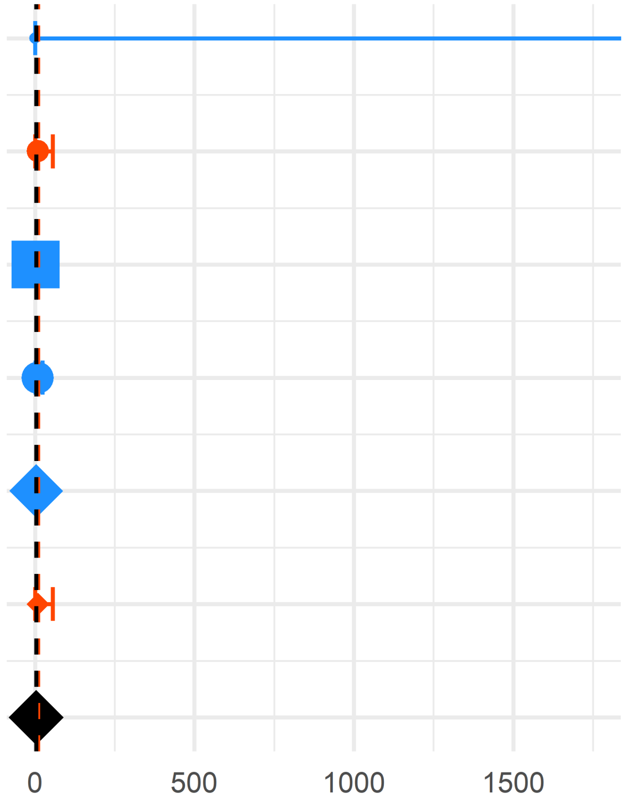

Heterogeneity:

1) $\tau^2 = 0.15$, $I^2 = 25.88$ %, $\chi^2 = 2.73$ (df = 2 , p-value = 0.256)

2) τ^2 = . , I^2 = . χ^2 = .

3) $\tau^2 = 0.16$, $I^2 = 6.9$ %, $\chi^2 = 3.47$ (df = 3 , p-value = 0.325)

Sigmoidoscopy shortterm/not reported

Study	Events	People	Weight	Point estimate per 100.000 people, 95% CI
Jain 2002	0	4229	13.79 %	0 [0 , 87]
Holme 2014	0	12955	13.79 %	0 [0 , 28]
Zubarik 2002	0	185	13.79 %	0 [0 , 1974]
Aktin 2002	1	40674	17.24 %	2 [0 , 14]
Kewenter 1996	3	2108	20.69 %	142 [29 , 415]
Schoen 2012	3	67071	20.69 %	4 [1 , 13]
1) Subtotal (not critical)	1	40674	17.24 %	2 [0 , 14]
2) Subtotal (critical)	6	86548	82.75 %	5 [0 , 84]
3) Total	7	127222	100 %	5 [1 , 36]

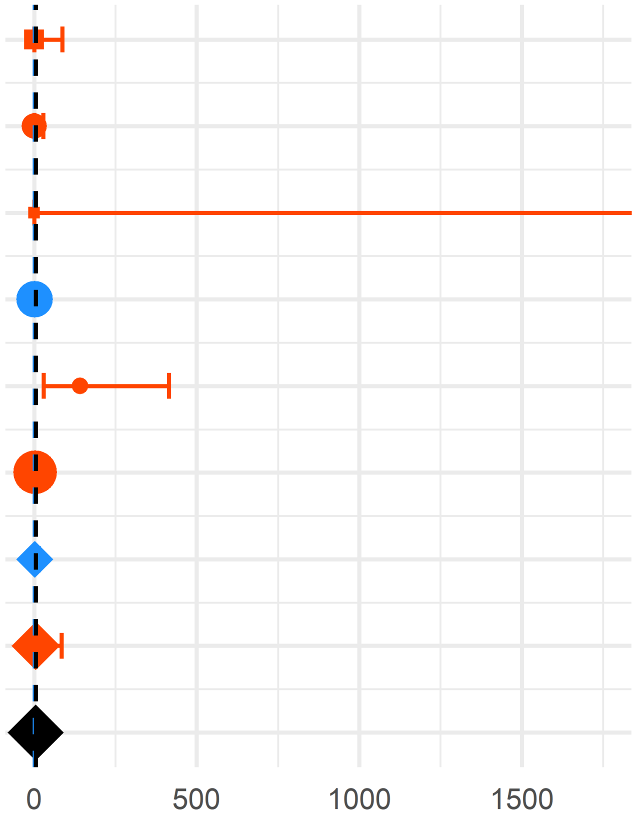

Heterogeneity:

1) τ^2 = . , I^2 = . , χ^2 = .

2) τ^2 = 3.81 , I^2 = 77.73 %, χ^2 = 15.5 (df = 4 , p-value = 0.0038

3) τ^2 = 3.14 , I^2 = 77.88 %, χ^2 = 16.66 (df = 5 , p-value = 0.005

Colonoscopy following any screening tests longterm

Study	Events	People	Weight	Point estimate per 100.000 people, 95% CI
Polter 2015	0	3059	3.33 %	0 [0 , 121]
Forbes 2006	0	112	3.33 %	0 [0 , 3240]
Shroff 2015	1	6152	6.67 %	16 [0 , 91]
Castro 2013	3	3215	20 %	93 [19 , 272]
Randel 2021	4	3297	26.67 %	121 [33 , 310]
Rutter 2012	6	4984	40 %	120 [44 , 262]
1) Subtotal (not critical)	4	3409	30 %	117 [44 , 313]
2) Subtotal (critical)	10	17410	70 %	46 [15 , 140]
3) Total	14	20819	100 %	59 [26 , 134]

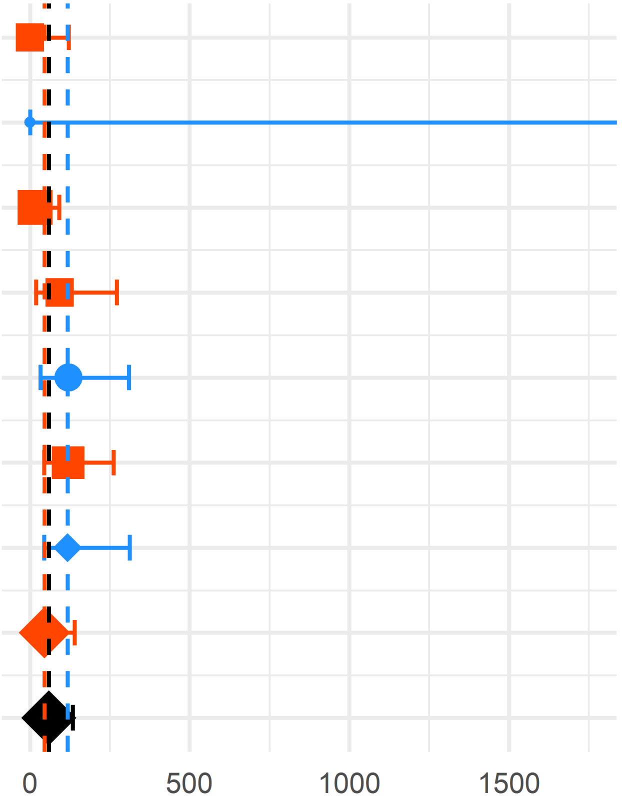

Heterogeneity:

1) $\tau^2 = 0$, $I^2 = 0$ %, $\chi^2 = 0.27$ (df = 1 , p-value = 0.6052)

2) $\tau^2 = 0.51$, $I^2 = 12.75$ %, $\chi^2 = 9.27$ (df = 3 , p-value = 0.0259

3) $\tau^2 = 0.31$, $I^2 = 0$ %, $\chi^2 = 10.83$ (df = 5 , p-value = 0.0548)

Colonoscopy following any screening tests shortterm/not reported

Study	Events	People	Weight	Point estimate per 100.000 people, 95% CI
Dellon 2009	0	3614	4.72 %	0 [0 , 102]
Segnan 2002	1	775	7.33 %	129 [3 , 717]
Naumann 2021	2	5219	10.13 %	38 [5 , 138]
Kewenter 1996	2	185	10.13 %	1081 [131 , 3850]
Mandel 1993	4	11943	12.53 %	33 [9 , 86]
Aktin 2002	4	2051	12.53 %	195 [53 , 499]
Holme 2014	6	2746	13.6 %	218 [80 , 475]
Gondal 2003	6	2524	13.6 %	238 [87 , 517]
Schoen 2012	19	17234	15.41 %	110 [66 , 172]
1) Subtotal (not critical)	4	5665	17.25 %	32 [1 , 1270]
2) Subtotal (critical)	40	40626	82.73 %	115 [56 , 233]
3) Total	44	46291	100 %	100 [50 , 201]

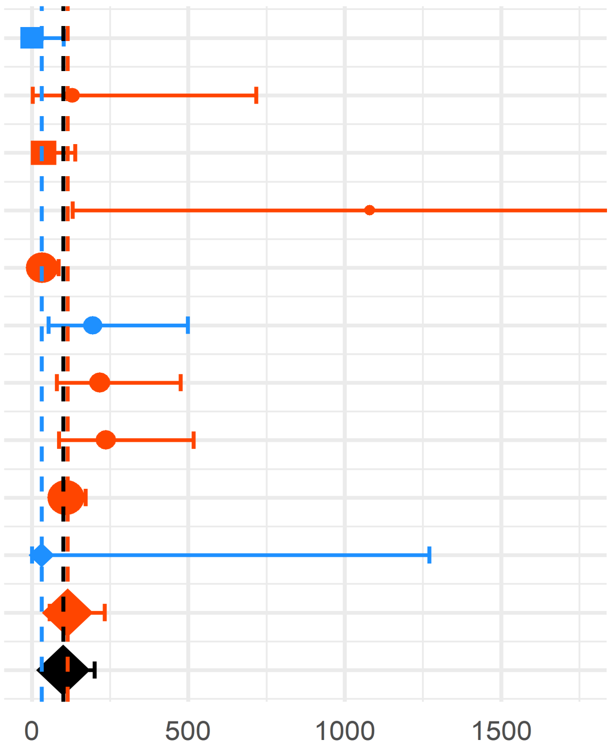

Heterogeneity:

1) $\tau^2 = 3.27$, $I^2 = 0$ %, $\chi^2 = 8.13$ (df = 1 , p-value = 0.0044)

2) $\tau^2 = 0.57$, $I^2 = 74.61$ %, $\chi^2 = 22.16$ (df = 6 , p-value = 0.001

3) $\tau^2 = 0.75$, $I^2 = 66.99$ %, $\chi^2 = 30.73$ (df = 8 , p-value = 2e-04
