# Supplementary material for: The risk of bleeding and perforation from sigmoidoscopy or colonoscopy in colorectal cancer screening: A systematic review and meta-analyses
Source: PLoS One. 2023 Oct 31;18(10):e0292797. doi: 10.1371/journal.pone.0292797 (PMC10617695; doi:10.1371/journal.pone.0292797)
Supplement: S2 Fig — (PDF) [file pone.0292797.s007.pdf]

S4 - Figures

Meta-analyses of bleeding (Subcategories)

Once-only colonoscopy

- Severe-NR
- Severe-longterm
- Mild-NR
- Mild-longterm
- ND-longterm
- ND-NR

Colonoscopy following FIT

- Severe-NR
- Severe-longterm
- Mild-NR
- Mild-longterm
- ND-longterm
- ND-NR

Sigmoidoscopy

- Severe-NR
- Mild-NR
- Mild-longterm
- ND-longterm
- ND-NR

Colonoscopy following any screening tests

- Severe-NR
- Severe-longterm
- Mild-NR
- ND-longterm
- ND-NR

Once-only colonoscopy

Once-only colonoscopy categorized as: Severe-NR

Study	Events	People	Weight	Point estimate per 100.000 people, 95% CI
Dae 2007	0	4491	7.8 %	0 [0 , 82]
Schoenfeld 2005	0	1483	7.8 %	0 [0 , 248]
Taleban 2018	1	99	10.98 %	1010 [26 , 5500]
Bretthauer 2016	18	11912	17.88 %	151 [90 , 239]
Crispin 2009	134	55993	18.47 %	239 [201 , 283]
Bokemeyer 2009	442	264602	18.53 %	167 [152 , 183]
Pox 2012	573	2773783	18.54 %	21 [19 , 22]
1) Subtotal (not critical)	461	276613	47.39 %	167 [152 , 183]
2) Subtotal (critical)	707	2835750	52.61 %	23 [2 , 226]
3) Total	1168	3112363	100 %	72 [24 , 219]

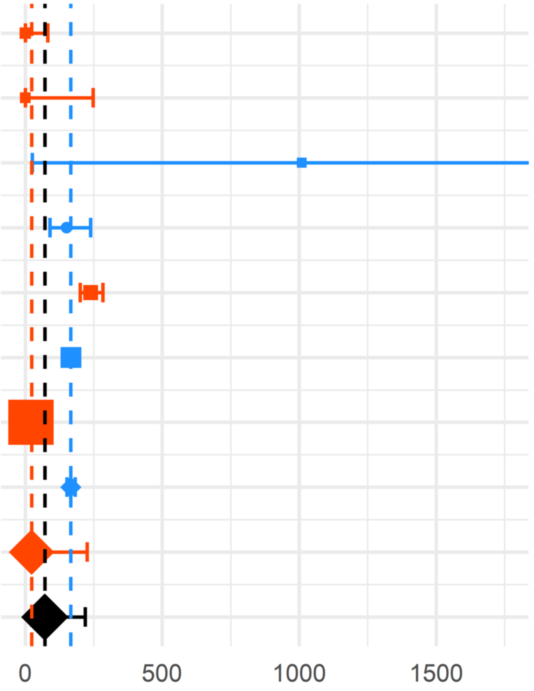

Heterogeneity:

1) $\tau^2 = 0$, $I^2 = 41.51$ %, $\chi^2 = 2.11$ (df = 2 , p-value = 0.3474)

2) $\tau^2 = 2.68$, $I^2 = 99.54$ %, $\chi^2 = 390.61$ (df = 3 , p-value = 0)

3) $\tau^2 = 1.63$, $I^2 = 99.57$ %, $\chi^2 = 1189.07$ (df = 6 , p-value = 0)

Once-only colonoscopy categorized as: Severe-longterm

Study	Events	People	Weight	Point estimate per 100.000 people, 95% CI
Pedersen 2020	3	11161	10.7 %	27 [6 , 79]
Stock 2013 (23a)	4	8658	12 %	46 [13 , 118]
Berhane 2009	5	11808	12.94 %	42 [14 , 99]
Lieberman 2000	6	3121	13.66 %	192 [71 , 418]
Nelson 2002	7	3196	14.22 %	219 [88 , 451]
Garcia-Albeniz 2017	34	78065	17.7 %	44 [30 , 61]
Wang 2018 (s28)	405	645095	18.79 %	63 [57 , 69]
1) Subtotal (not critical)	459	749296	87.07 %	68 [39 , 118]
2) Subtotal (critical)	5	11808	12.94 %	42 [14 , 99]
3) Total	464	761104	100 %	63 [39 , 103]

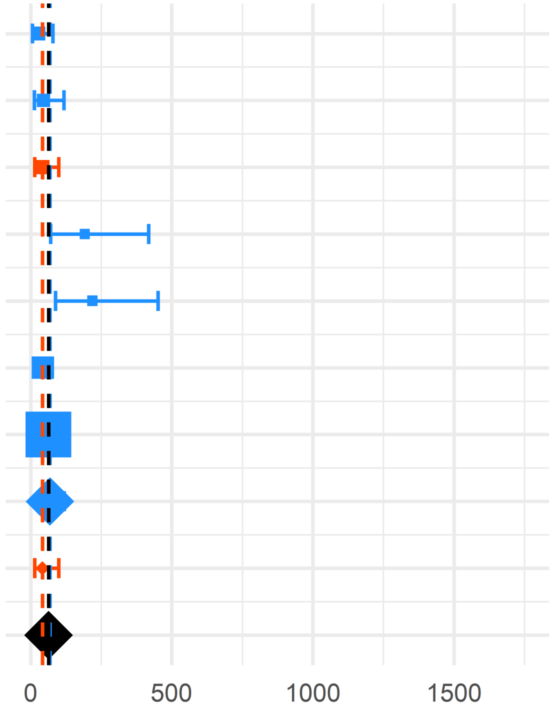

Heterogeneity:

1) $\tau^2 = 0.35$, $I^2 = 80.41$ %, $\chi^2 = 21.07$ (df = 5 , p-value = 8e-04

2) τ^2 = . , I^2 = . χ^2 = .

3) $\tau^2 = 0.29$, $I^2 = 77.17$ %, $\chi^2 = 21.84$ (df = 6 , p-value = 0.001

Once-only colonoscopy categorized as: Mild-NR

Study	Events	People	Weight	Point estimate per 100.000 people, 95% CI
Imperiale 2000	3	1994	26.75 %	150 [31 , 439]
Xirasagar 2020	6	25008	32.35 %	24 [9 , 52]
Pox 2012	2423	2773783	40.9 %	87 [84 , 91]
1) Subtotal (not critical)	6	25008	32.35 %	24 [9 , 52]
2) Subtotal (critical)	2426	2775777	67.65 %	87 [84 , 91]
3) Total	2432	2800785	100 %	62 [28 , 137]

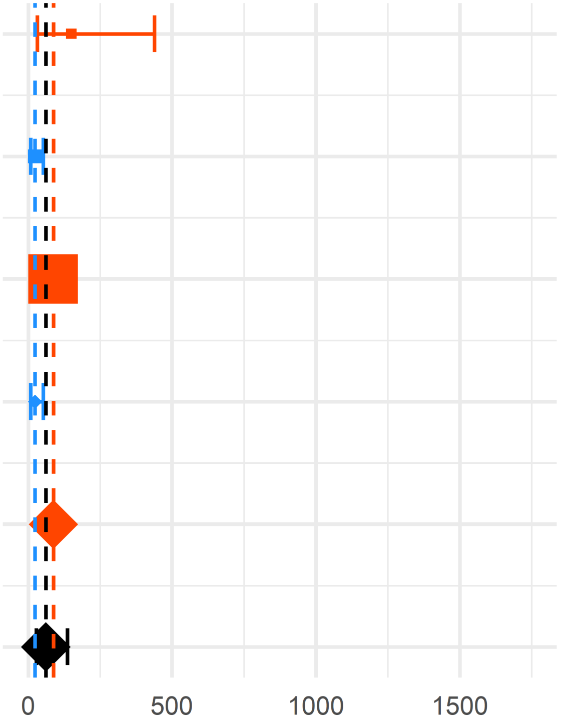

Heterogeneity:

1) τ^2 = . , I^2 = . , χ^2 = .

2) $\tau^2 = 0$, $I^2 = 0$ %, $\chi^2 = 0.75$ (df = 1 , p-value = 0.388)

3) $\tau^2 = 0.35$, $I^2 = 81.64$ %, $\chi^2 = 16.84$ (df = 2 , p-value = 2e-04

Once-only colonoscopy categorized as: Mild-longterm

Study	Events	People	Weight	Point estimate per 100.000 people, 95% CI
Nelson 2002	6	3196	22.93 %	188 [69 , 408]
Garcia-Albeniz 2017 (58c)	48	31193	25.58 %	154 [113 , 204]
Garcia-Albeniz 2017 (58a)	55	46872	25.63 %	117 [88 , 153]
Pedersen 2020	148	11163	25.86 %	1326 [1122 , 1556]
1) Subtotal (not critical)	257	92424	100 %	258 [97 , 685]
2) Total	257	92424	100 %	258 [97 , 685]

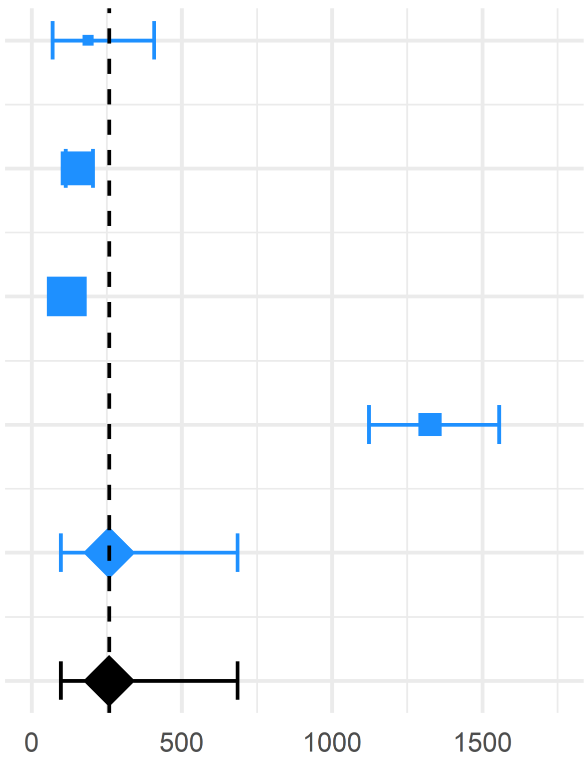

Heterogeneity:

1) $\tau^2 = 0.95$, $I^2 = 99.09$ %, $\chi^2 = 305.91$ (df = 3 , p-value = 0)

2) $\tau^2 = 0.95$, $I^2 = 99.09$ %, $\chi^2 = 305.91$ (df = 3 , p-value = 0)

Once-only colonoscopy categorized as: ND-longterm

Study	Events	People	Weight	Point estimate per 100.000 people, 95% CI
Khalid-de Bakker 2011	0	214	9.28 %	0 [0 , 1709]
Khalid-de Bakker 2011b	0	447	9.28 %	0 [0 , 822]
Stoop 2012 (13b)	2	1276	12.36 %	157 [19 , 565]
Senore 2011 (42b)	15	1198	13.67 %	1252 [702 , 2057]
Zwink 2017	35	5252	13.8 %	666 [465 , 926]
Causada-Calo 2020	76	20443	13.85 %	372 [293 , 465]
Rutter 2012 (7a)	103	38472	13.86 %	268 [219 , 325]
Wong 2017	500	833	13.89 %	60024 [56607 , 63370]
1) Subtotal (not critical)	111	26356	46.21 %	423 [264 , 677]
2) Subtotal (critical)	620	41779	53.78 %	1314 [128 , 13544]
3) Total	731	68135	100 %	461 [85 , 2504]

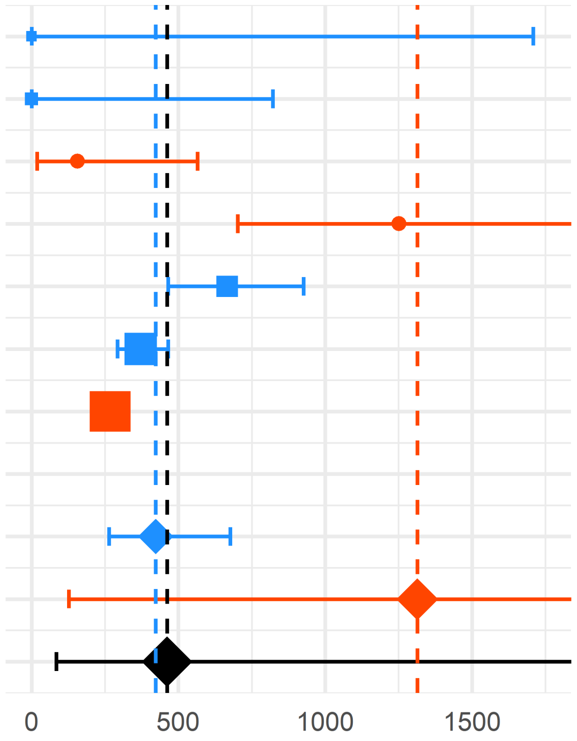

Heterogeneity:

1) $\tau^2 = 0.08$, $I^2 = 63.25$ %, $\chi^2 = 13.16$ (df = 3 , p-value = 0.004

2) $\tau^2 = 5.57$, $I^2 = 99.89$ %, $\chi^2 = 3333.13$ (df = 3 , p-value = 0)

3) $\tau^2 = 4.93$, $I^2 = 99.83$ %, $\chi^2 = 3540.97$ (df = 7 , p-value = 0)

Once-only colonoscopy categorized as: ND-NR

Study	Events	People	Weight	Point estimate per 100.000 people, 95% CI
Strul 2006	0	1177	1.43 %	0 [0 , 313]
Ahmed 2016	1	1049	2.63 %	95 [2 , 530]
Huppe 2004	4	1117	7.18 %	358 [98 , 914]
Quintero 2012 (27a)	12	5059	11.66 %	237 [123 , 414]
Zwink 2017	19	5252	13.17 %	362 [218 , 564]
Ferlitsch 2011 (3c)	54	44350	15.38 %	122 [91 , 159]
Kozibal 2015 (3b)	71	59901	15.73 %	119 [93 , 149]
Waldmann 2016 (3a)	165	159246	16.39 %	104 [88 , 121]
Sieg 2006	183	108133	16.44 %	169 [146 , 196]
1) Subtotal (not critical)	239	209897	47.57 %	150 [87 , 257]
2) Subtotal (critical)	270	175387	52.44 %	155 [119 , 201]
3) Total	509	385284	100 %	154 [113 , 211]

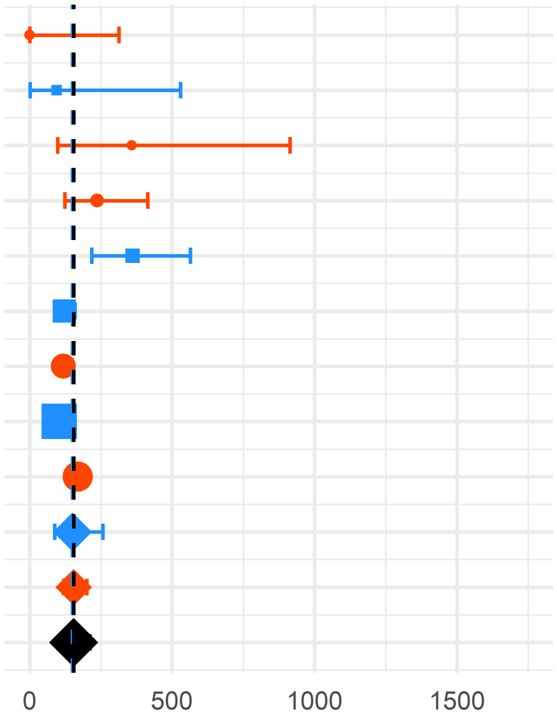

Heterogeneity:

1) $\tau^2 = 0.22$, $I^2 = 88.78$ %, $\chi^2 = 19.67$ (df = 3 , p-value = 2e-04

2) $\tau^2 = 0.03$, $I^2 = 64.88$ %, $\chi^2 = 14.66$ (df = 4 , p-value = 0.005

3) $\tau^2 = 0.14$, $I^2 = 83.54$ %, $\chi^2 = 45.9$ (df = 8 , p-value = 0)

Colonoscopy following FIT

Colonoscopy following FIT categorized as: Severe-NR

Study	Events	People	Weight	Point estimate per 100.000 people, 95% CI
Robinson 1999	1	1249	8.27 %	80 [2 , 445]
Denters 2013	1	273	8.27 %	366 [9 , 2024]
Tepes 2017	4	13674	12.51 %	29 [8 , 75]
Garcia 2012	6	989	13.27 %	607 [223 , 1316]
Gupta 2012	8	1057	13.68 %	757 [327 , 1486]
Binefa 2013	15	1691	14.3 %	887 [497 , 1459]
Parente 2013	29	3698	14.67 %	784 [526 , 1124]
Quyn 2018	213	53332	15.03 %	399 [348 , 457]
1) Subtotal (not critical)	8	1057	13.68 %	757 [327 , 1486]
2) Subtotal (critical)	269	74906	86.32 %	281 [112 , 705]
3) Total	277	75963	100 %	324 [145 , 728]

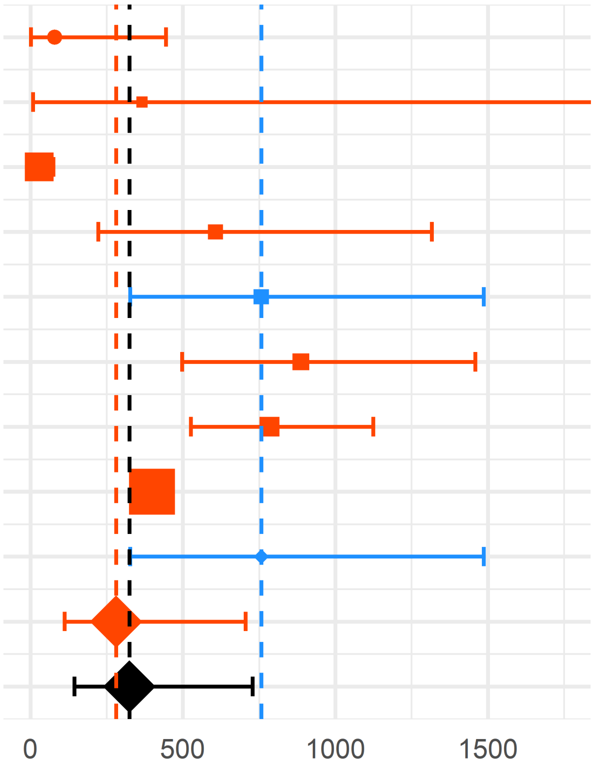

Heterogeneity:

1) τ^2 = . , I^2 = . , χ^2 = .

2) τ^2 = 1.27 , I^2 = 88.45 %, χ^2 = 100.97 (df = 6 , p-value = 0)

3) τ^2 = 1.15 , I^2 = 87.12 %, χ^2 = 104.43 (df = 7 , p-value = 0)

Colonoscopy following FIT categorized as: Severe-longterm

Study	Events	People	Weight	Point estimate per 100.000 people, 95% CI
Lee 2012 (2b)	4	30829	5.67 %	13 [4 , 33]
IbÃ¡nez 2018	10	7467	6.34 %	134 [64 , 246]
Din 2015 (2d)	12	38242	6.42 %	31 [16 , 55]
Robertson 2019	31	17485	6.69 %	177 [120 , 252]
Dominitz 2019	31	14661	6.69 %	211 [144 , 300]
Denis 2021 (s4b)	43	9061	6.74 %	475 [344 , 639]
Randel 2021 (s23b)	44	6945	6.75 %	634 [461 , 850]
Mikkelsen 2018	60	14671	6.78 %	409 [312 , 526]
Vanaclocha-Espi 2018	63	41204	6.78 %	153 [118 , 196]
Tomaszewski 2021	203	78831	6.85 %	258 [223 , 295]
Paszat 2020	219	51310	6.85 %	427 [372 , 487]
Arana-Arri 2018	245	36346	6.85 %	674 [593 , 764]
Benazzato 2020	281	99674	6.85 %	282 [250 , 317]
Portillo 2018	330	48759	6.86 %	677 [606 , 754]
Hsu 2020	2125	214955	6.87 %	989 [947 , 1031]
1) Subtotal (not critical)	3456	674094	93.14 %	229 [129 , 408]
2) Subtotal (critical)	245	36346	6.85 %	674 [593 , 764]
3) Total	3701	710440	100 %	247 [142 , 429]

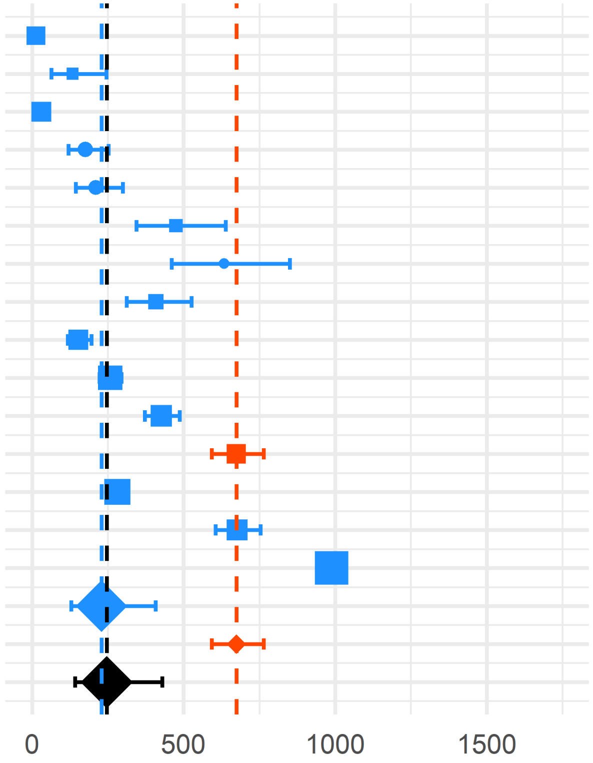

Heterogeneity:

1) τ^2 = 1.17 , I^2 = 98.93 %, χ^2 = 1866.23 (df = 13 , p-value = 0)

2) τ^2 = . , I^2 = . χ^2 = .

3) τ^2 = 1.16 , I^2 = 98.85 %, χ^2 = 1882.08 (df = 14 , p-value = 0)

Colonoscopy following FIT categorized as: Mild-NR

Study	Events	People	Weight	Point estimate per 100.000 people, 95% CI
Cheng 2002	5	6266	49.52 %	80 [26 , 186]
Ghanouni 2016 (2c)	3865	50858	50.48 %	7600 [7371 , 7833]
1) Subtotal (not critical)	3865	50858	50.48 %	7600 [7371 , 7833]
2) Subtotal (critical)	5	6266	49.52 %	80 [26 , 186]
3) Total	3870	57124	100 %	776 [31 , 19343]

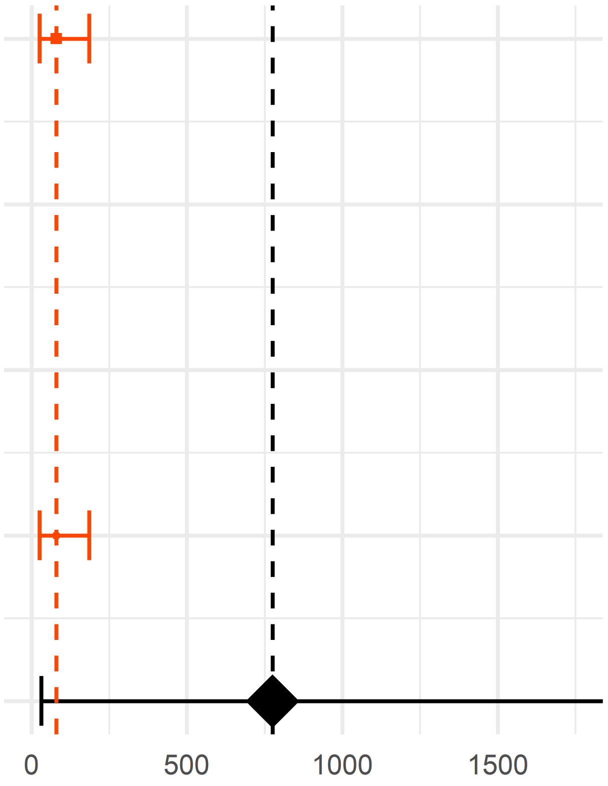

Heterogeneity:

1) τ^2 = . , I^2 = . , χ^2 = .

2) τ^2 = . , I^2 = . , χ^2 = .

3) τ^2 = 5.31 , I^2 = 99.04 %, χ^2 = 843.72 (df = 1 , p-value = 0)

Colonoscopy following FIT categorized as: Mild-longterm

Study	Events	People	Weight	Point estimate per 100.000 people, 95% CI
Denis 2021 (s4a)	69	17152	23.98 %	402 [313 , 509]
Lee 2012 (2b)	151	30829	25.03 %	490 [415 , 574]
Robertson 2019	182	17485	25.19 %	1041 [896 , 1203]
Rutter 2014 (2a)	847	112024	25.81 %	756 [706 , 809]
1) Subtotal (not critical)	1249	177490	100 %	631 [440 , 905]
2) Total	1249	177490	100 %	631 [440 , 905]

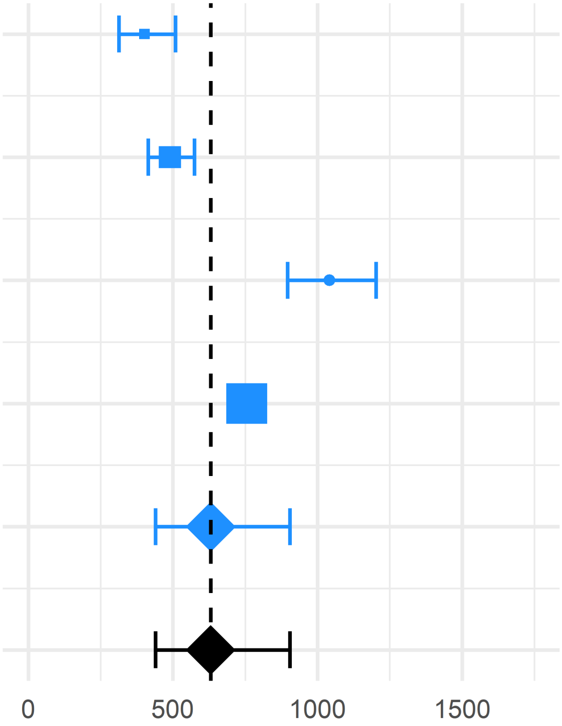

Heterogeneity:

1) $\tau^2 = 0.13$, $I^2 = 95.86$ %, $\chi^2 = 77.53$ (df = 3 , p-value = 0)

2) $\tau^2 = 0.13$, $I^2 = 95.86$ %, $\chi^2 = 77.53$ (df = 3 , p-value = 0)

Colonoscopy following FIT categorized as: ND-longterm

Study	Events	People	Weight	Point estimate per 100.000 people, 95% CI
Hughes 2005	2	92	1.5 %	2174 [264 , 7633]
Saraste 2016	18	2984	10.06 %	603 [358 , 952]
Denis 2013	97	10024	23.95 %	968 [785 , 1179]
Karlijn 2021	401	47270	31.44 %	848 [768 , 935]
Blanks 2015 (2e)	777	112024	33.04 %	694 [646 , 744]
1) Subtotal (not critical)	1295	172394	100 %	793 [687 , 914]
2) Total	1295	172394	100 %	793 [687 , 914]

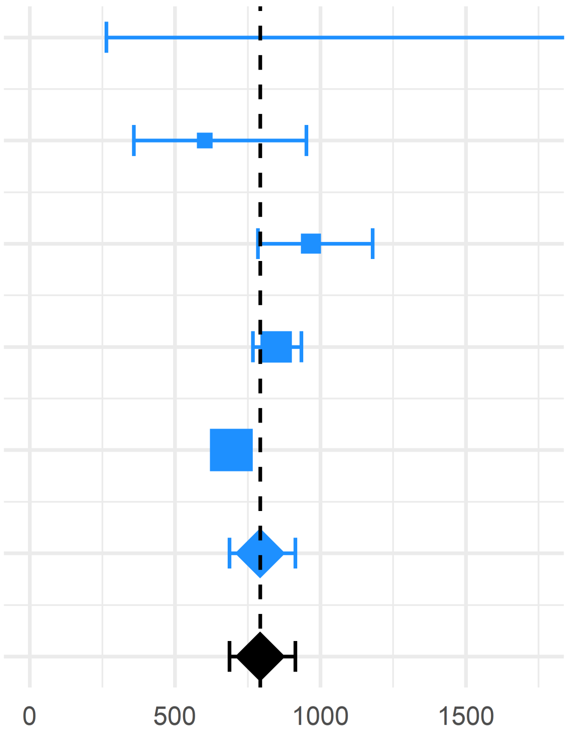

Heterogeneity:

1) $\tau^2 = 0.01$, $I^2 = 80.15$ %, $\chi^2 = 19.07$ (df = 4 , p-value = 8e-04

2) $\tau^2 = 0.01$, $I^2 = 80.15$ %, $\chi^2 = 19.07$ (df = 4 , p-value = 8e-04

Colonoscopy following FIT categorized as: ND-NR

Study	Events	People	Weight	Point estimate per 100.000 people, 95% CI
Dancourt 2008	0	1205	4.75 %	0 [0 , 306]
Faivre 2004	0	1339	4.75 %	0 [0 , 275]
Din 2017	1	780	6.55 %	128 [3 , 712]
Sung 2003	1	476	6.55 %	210 [5 , 1165]
Ellul 2010 (2f)	3	1039	8.78 %	289 [60 , 841]
Denis 2007	4	2310	9.16 %	173 [47 , 443]
Denters 2012	5	491	9.41 %	1018 [331 , 2360]
Neely 2013	6	176	9.59 %	3409 [1261 , 7272]
Quintero 2012 (27b)	8	663	9.82 %	1207 [522 , 2364]
Marino 2012	11	1000	10.01 %	1100 [550 , 1960]
Meulen 2021	13	225	10.09 %	5778 [3112 , 9678]
Zorzi 2009	190	29276	10.53 %	649 [560 , 748]
1) Subtotal (not critical)	1	476	6.55 %	210 [5 , 1165]
2) Subtotal (critical)	241	38504	93.44 %	443 [160 , 1232]
3) Total	242	38980	100 %	416 [160 , 1087]

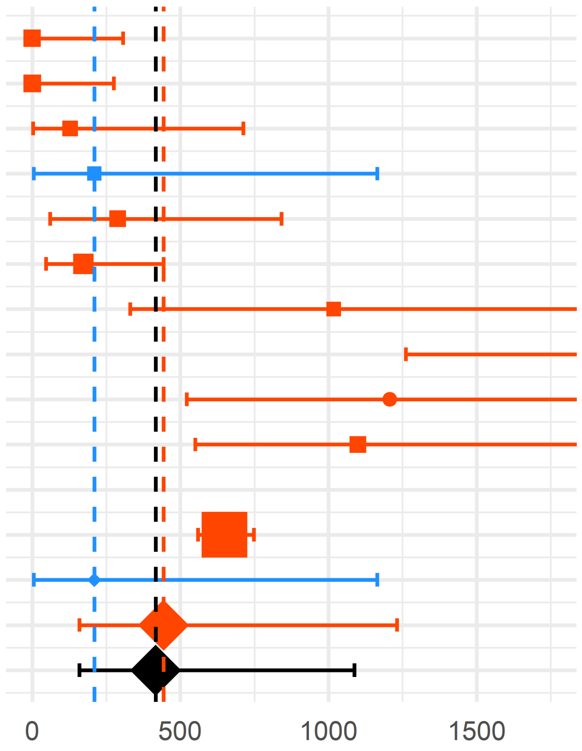

Heterogeneity:

1) τ^2 = . , I^2 = . , χ^2 = .

2) τ^2 = 2.5 , I^2 = 88.89 %, χ^2 = 101.57 (df = 10 , p-value = 0)

3) τ^2 = 2.35 , I^2 = 88.01 %, χ^2 = 103.33 (df = 11 , p-value = 0)

Sigmoidoscopy

Sigmoidoscopy categorized as: Severe-NR

Study	Events	People	Weight	Point estimate per 100.000 people, 95% CI
Segnan 2002 (50a)	0	9911	13.92 %	0 [0 , 37]
Segnan 2005 (51a)	0	4466	13.92 %	0 [0 , 83]
Holme 2014 (s34a)	0	12955	13.92 %	0 [0 , 28]
Aktin 2002 (36a)	12	40674	58.25 %	30 [15 , 52]
1) Subtotal (not critical)	12	40674	58.25 %	30 [15 , 52]
2) Subtotal (critical)	0	27332	41.76 %	0 [0 , 13]
3) Total	12	68006	100 %	1 [0 , 32421]

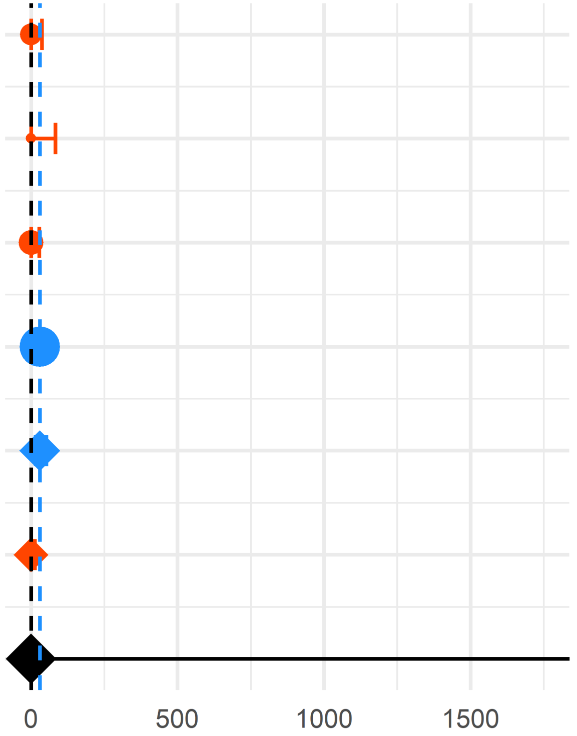

Heterogeneity:

1) τ^2 = . , I^2 = . , χ^2 = .

2) τ^2 = 0 , I^2 = 0 %, χ^2 = 0 (df = 0 , p-value = 1)

3) τ^2 = 4.64 , I^2 = 0 %, χ^2 = 12.34 (df = 3 , p-value = 0.0063)

Sigmoidoscopy categorized as: Mild-NR

Study	Events	People	Weight	Point estimate per 100.000 people, 95% CI
Segnan 2005 (51a)	5	4466	33.33 %	112 [36 , 261]
Segnan 2002 (50a)	10	9911	66.67 %	101 [48 , 185]
1) Subtotal (critical)	15	14377	100 %	104 [63 , 173]
2) Total	15	14377	100 %	104 [63 , 173]

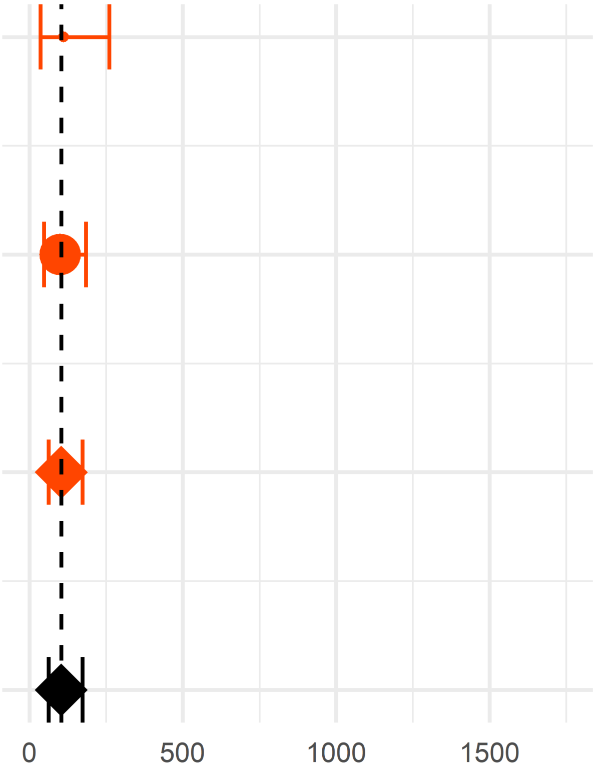

Heterogeneity:

1) $\tau^2 = 0$, $I^2 = 0$ %, $\chi^2 = 0.04$ (df = 1 , p-value = 0.8503)

2) $\tau^2 = 0$, $I^2 = 0$ %, $\chi^2 = 0.04$ (df = 1 , p-value = 0.8503)

Sigmoidoscopy categorized as: Mild-longterm

Study	Events	People	Weight	Point estimate per 100.000 people, 95% CI
Levin 2002	9	107704	49.5 %	8 [4 , 16]
Aktin 2013 (36a)	77	40674	50.5 %	189 [149 , 237]
1) Subtotal (not critical)	77	40674	50.5 %	189 [149 , 237]
2) Subtotal (critical)	9	107704	49.5 %	8 [4 , 16]
3) Total	86	148378	100 %	40 [4 , 356]

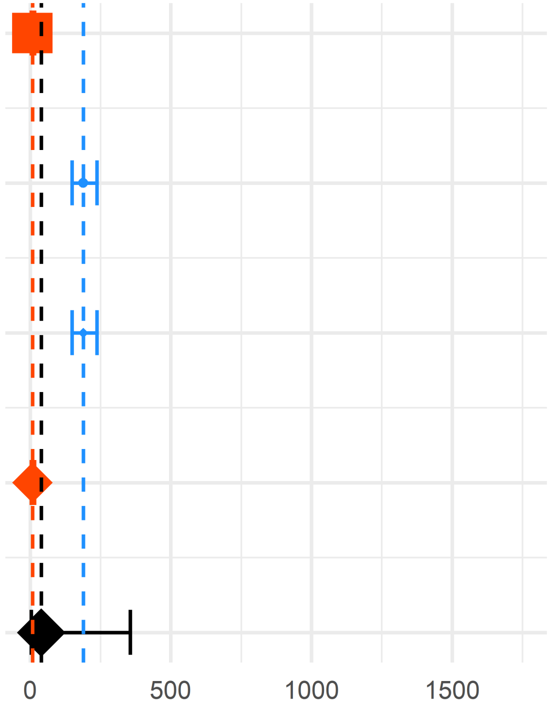

Heterogeneity:

1) τ^2 = . , I^2 = . , χ^2 = .

2) τ^2 = . , I^2 = . , χ^2 = .

3) τ^2 = 2.44 , I^2 = 98.73 %, χ^2 = 147.42 (df = 1 , p-value = 0)

Sigmoidoscopy categorized as: ND-longterm

Study	Events	People	Weight	Point estimate per 100.000 people, 95% CI
Forbes 2006 (63b)	0	39	30.64 %	0 [0 , 9025]
Levin 2002	2	107704	34.12 %	2 [0 , 7]
Senore 2011 (42a)	12	1502	35.24 %	799 [413 , 1391]
1) Subtotal (not critical)	2	107743	64.76 %	2 [0 , 7]
2) Subtotal (critical)	12	1502	35.24 %	799 [413 , 1391]
3) Total	14	109245	100 %	29 [1 , 1530]

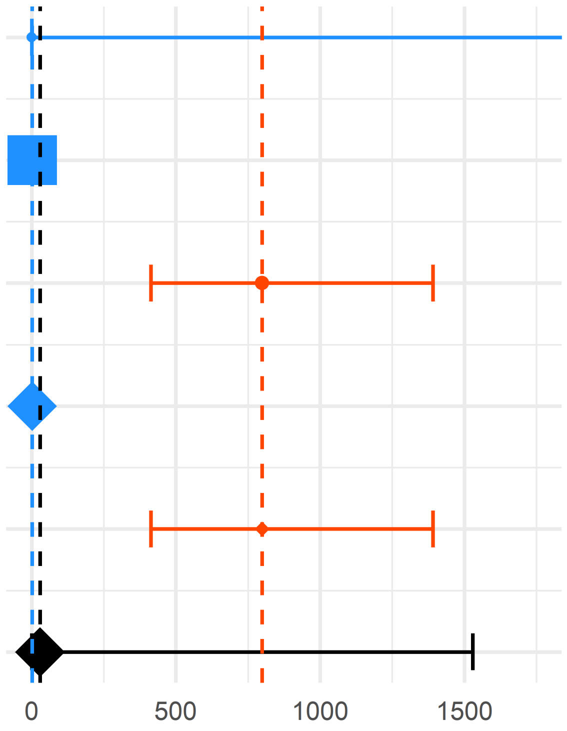

Heterogeneity:

1) $\tau^2 = 0$, $I^2 = 0$ %, $\chi^2 = 0$ (df = 1 , p-value = 0.9696)

2) $\tau^2 = .$, $I^2 = .$ $\chi^2 = .$

3) $\tau^2 = 8.75$, $I^2 = 96.83$ %, $\chi^2 = 91.46$ (df = 2 , p-value = 0)

Sigmoidoscopy categorized as: ND-NR

Study	Events	People	Weight	Point estimate per 100.000 people, 95% CI
Jain 2002	0	4229	21.89 %	0 [0 , 87]
Kewenter 1996 (85a)	0	2108	21.89 %	0 [0 , 175]
Pabby 2005	3	6968	27.44 %	43 [9 , 126]
Aktin 1998	40	1306	28.79 %	3063 [2197 , 4147]
1) Subtotal (not critical)	3	6968	27.44 %	43 [9 , 126]
2) Subtotal (critical)	40	7643	72.57 %	0 [0 , 2225]
3) Total	43	14611	100 %	15 [0 , 3474]

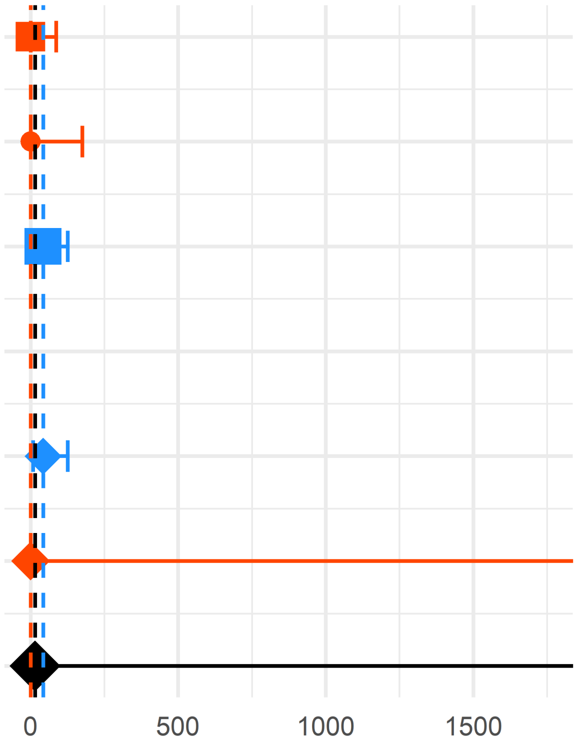

Heterogeneity:

1) τ^2 = . , I^2 = . , χ^2 = .

2) τ^2 = 30.79 , I^2 = 0 %, χ^2 = 141.35 (df = 2 , p-value = 0)

3) τ^2 = 11.94 , I^2 = 94.09 %, χ^2 = 175.87 (df = 3 , p-value = 0)

Colonoscopy following any screening tests

Colonoscopy following any screening tests categorized as: Severe-NR

Study	Events	People	Weight	Point estimate per 100.000 people, 95% CI
Segnan 2002 (50b)	1	775	10.16 %	129 [3 , 717]
Segnan 2005 (51b)	1	332	10.16 %	301 [8 , 1667]
Holme 2014 (s34b)	4	2746	22.08 %	146 [40 , 373]
Aktin 2002 (36b	9	2051	28.21 %	439 [201 , 831]
Mandel 1993	11	11941	29.39 %	92 [46 , 165]
1) Subtotal (not critical)	9	2051	28.21 %	439 [201 , 831]
2) Subtotal (critical)	17	15794	71.79 %	108 [67 , 173]
3) Total	26	17845	100 %	170 [87 , 330]

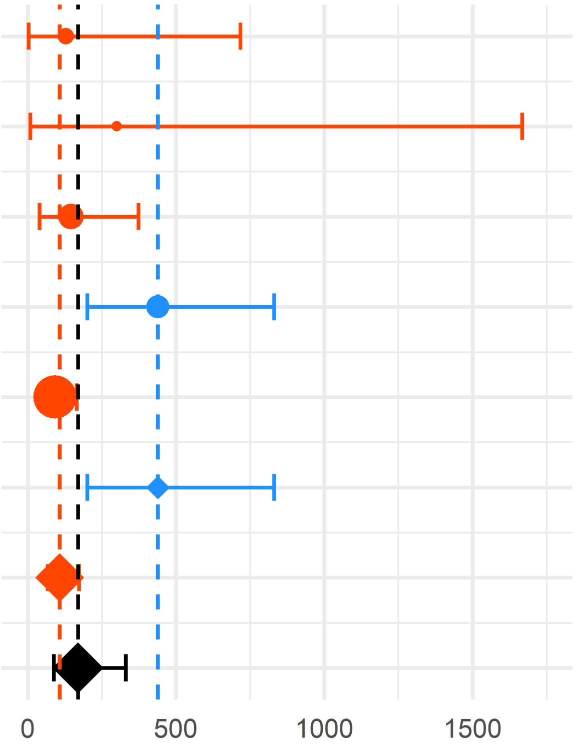

Heterogeneity:

1) τ^2 = . , I^2 = . , χ^2 = .

2) τ^2 = 0 , I^2 = 0 %, χ^2 = 1.42 (df = 3 , p-value = 0.7017)

3) τ^2 = 0.27 , I^2 = 68.35 %, χ^2 = 10.97 (df = 4 , p-value = 0.026

Colonoscopy following any screening tests categorized as: Severe-longterm

Study	Events	People	Weight	Point estimate per 100.000 people, 95% CI
Kewenter 1996 (85b)	1	185	28.26 %	541 [14 , 2975]
Shroff 2015	2	6152	32.94 %	33 [4 , 117]
Randel 2021 (s23c)	23	3297	38.8 %	698 [443 , 1045]
1) Subtotal (not critical)	23	3297	38.8 %	698 [443 , 1045]
2) Subtotal (critical)	3	6337	61.2 %	47 [15 , 147]
3) Total	26	9634	100 %	198 [36 , 1082]

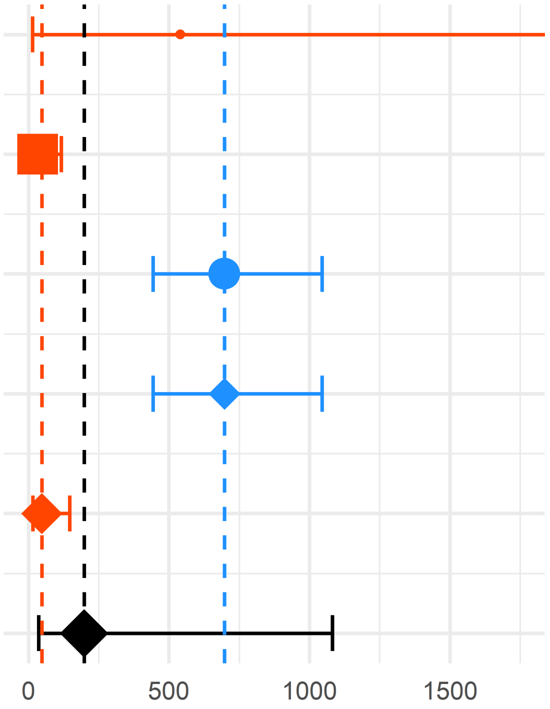

Heterogeneity:

1) τ^2 = . , I^2 = . , χ^2 = .

2) τ^2 = 0 , I^2 = 81.02 %, χ^2 = 3.37 (df = 1 , p-value = 0.0665)

3) τ^2 = 1.77 , I^2 = 88.44 %, χ^2 = 36.61 (df = 2 , p-value = 0)

Colonoscopy following any screening tests categorized as: Mild-NR

Study	Events	People	Weight	Point estimate per 100.000 people, 95% CI
Gondal 2003 (60b)	4	2524	23.52 %	158 [43 , 405]
Segnan 2002 (50b)	7	775	25.38 %	903 [364 , 1852]
Aktin 2002 (36b)	7	2051	25.38 %	341 [137 , 702]
Segnan 2005 (51b)	8	332	25.72 %	2410 [1046 , 4693]
1) Subtotal (not critical)	7	2051	25.38 %	341 [137 , 702]
2) Subtotal (critical)	19	3631	74.62 %	676 [192 , 2381]
3) Total	26	5682	100 %	562 [208 , 1515]

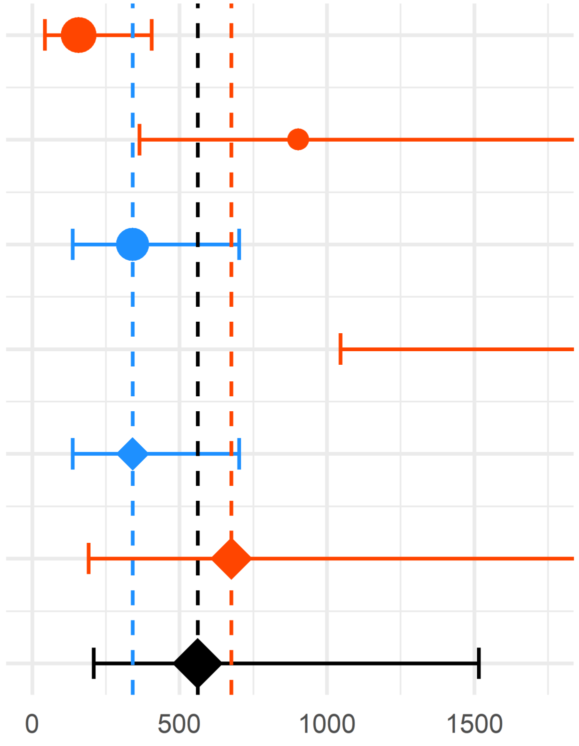

Heterogeneity:

1) τ^2 = . , I^2 = . , χ^2 = .

2) τ^2 = 1.09 , I^2 = 89.89 %, χ^2 = 22.52 (df = 2 , p-value = 0)

3) τ^2 = 0.88 , I^2 = 88.05 %, χ^2 = 23.51 (df = 3 , p-value = 0)

Colonoscopy following any screening tests categorized as: ND-longterm

Study	Events	People		Weight	Point estimate per 100.000 people, 95% CI
Forbes 2006 (63a)	0	112		20.91 %	0 [0 , 3240]
Castro 2013	1	3215		29.7 %	31 [1 , 173]
Rutter 2012 (7b)	19	4984		49.39 %	381 [230 , 595]
1) Subtotal (not critical)	0	112		20.91 %	0 [0 , 3240]
2) Subtotal (critical)	20	8199		79.09 %	128 [21 , 787]
3) Total	20	8311		100 %	113 [18 , 708]

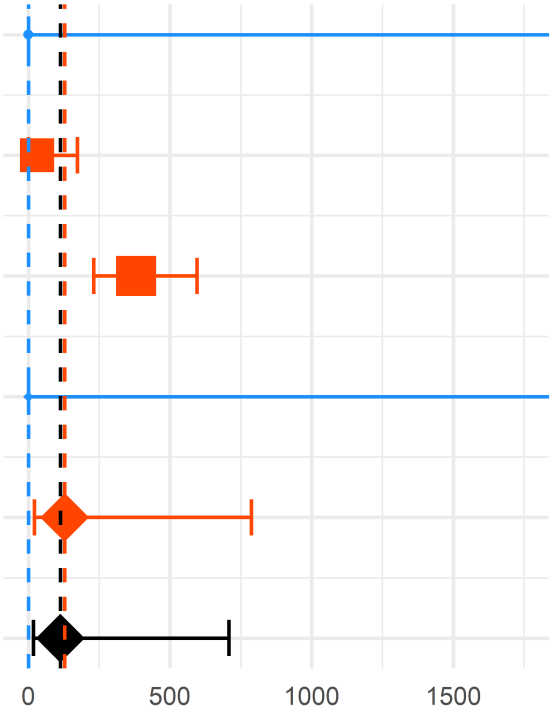

Heterogeneity:

1) τ^2 = . , I^2 = . , χ^2 = .

2) τ^2 = 1.28 , I^2 = 83.24 %, χ^2 = 12.85 (df = 1 , p-value = 3e-04

3) τ^2 = 1.24 , I^2 = 66.48 %, χ^2 = 13.39 (df = 2 , p-value = 0.001

Colonoscopy following any screening tests categorized as: ND-NR

Study	Events	People	Weight	Point estimate per 100.000 people, 95% CI
Naumann 2021	15	5219	45.45 %	287 [161 , 474]
Rajendran 2017	18	4953	54.55 %	363 [216 , 574]
1) Subtotal (critical)	33	10172	100 %	324 [231 , 456]
2) Total	33	10172	100 %	324 [231 , 456]

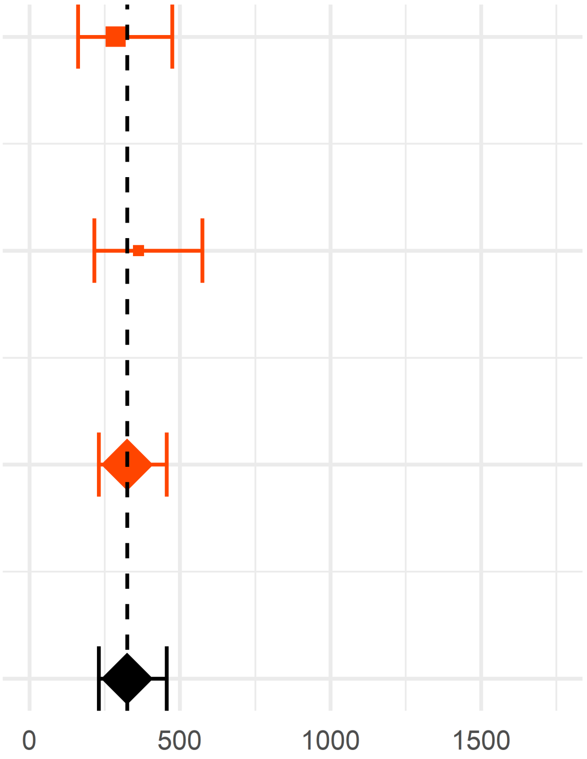

Heterogeneity:

1) $\tau^2 = 0$, $I^2 = 0$ %, $\chi^2 = 0.45$ (df = 1 , p-value = 0.5011)

2) $\tau^2 = 0$, $I^2 = 0$ %, $\chi^2 = 0.45$ (df = 1 , p-value = 0.5011)
