# Supplementary material for: The risk of bleeding and perforation from sigmoidoscopy or colonoscopy in colorectal cancer screening: A systematic review and meta-analyses
Source: PLoS One. 2023 Oct 31;18(10):e0292797. doi: 10.1371/journal.pone.0292797 (PMC10617695; doi:10.1371/journal.pone.0292797)
Supplement: S3 Fig — (PDF) [file pone.0292797.s008.pdf]

S5 - Figures

Meta-analyses of perforation (Subcategories)

Once-only colonoscopy

- Severe-NR
- Severe-longterm
- ND-longterm
- ND-NR

Colonoscopy following FIT

- Severe-NR
- Severe-longterm
- Mild-longterm
- ND-longterm
- ND-NR

Sigmoidoscopy

- Mild-longterm
- ND-longterm
- ND-NR

Colonoscopy following any screening tests

- Severe-NR
- Severe-longterm
- ND-longterm
- ND-NR

Once-only colonoscopy

Once-only colonoscopy categorized as: Severe-NR

Study	Events	People	Weight	Point estimate per 100.000 people, 95% CI
Taleban 2018	1	99	10.51 %	1010 [26 , 5500]
Leventi 2021	2	2298	13.63 %	87 [11 , 314]
Crispin 2009	22	55993	18.68 %	39 [25 , 59]
Sieg 2006	23	108133	18.71 %	21 [13 , 32]
Bokemeyer 2009	55	264602	19.11 %	21 [16 , 27]
Pox 2012	439	2773783	19.36 %	16 [14 , 17]
1) Subtotal (not critical)	56	264701	29.62 %	70 [5 , 937]
2) Subtotal (critical)	486	2940207	70.38 %	24 [15 , 39]
3) Total	542	3204908	100 %	23 [16 , 34]

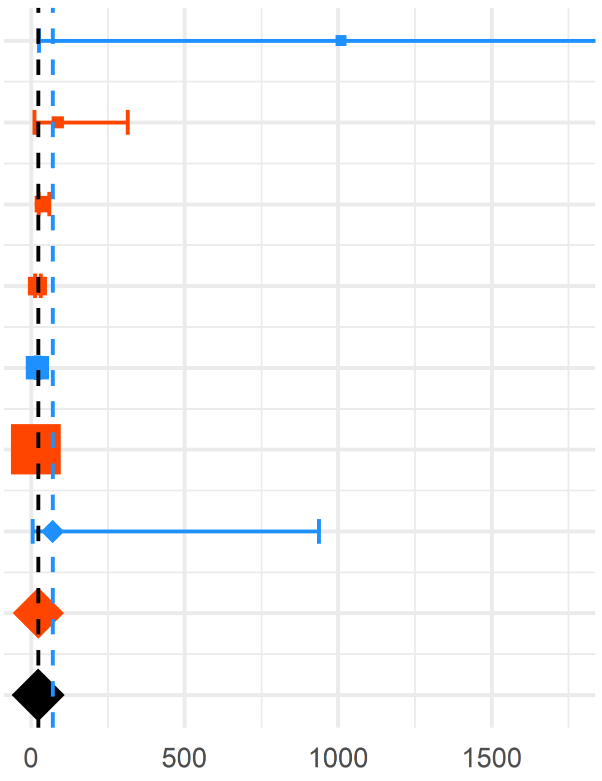

Heterogeneity:

1) $\tau^2 = 2.2$, $I^2 = 93.25$ %, $\chi^2 = 5.79$ (df = 1 , p-value = 0.0161)

2) $\tau^2 = 0.13$, $I^2 = 87.57$ %, $\chi^2 = 18.2$ (df = 3 , p-value = 4e-04)

3) $\tau^2 = 0.09$, $I^2 = 88.34$ %, $\chi^2 = 26.86$ (df = 5 , p-value = 1e-04

Once-only colonoscopy categorized as: Severe-longterm

Study	Events	People	Weight	Point estimate per 100.000 people, 95% CI
Nelson 2002	0	3196	4.52 %	0 [0 , 115]
Stock 2013	7	8658	14.44 %	81 [33 , 167]
Garcia-Albeniz 2017 (58c)	11	31193	15.39 %	35 [18 , 63]
Causada-Calo 2020	12	30443	15.54 %	39 [20 , 69]
Garcia-Albeniz 2017 (58a)	20	46872	16.23 %	43 [26 , 66]
Pedersen 2020	32	11163	16.64 %	287 [196 , 404]
Wang 2018 (s28)	186	645095	17.25 %	29 [25 , 33]
1) Subtotal (not critical)	268	776620	100 %	50 [26 , 97]
2) Total	268	776620	100 %	50 [26 , 97]

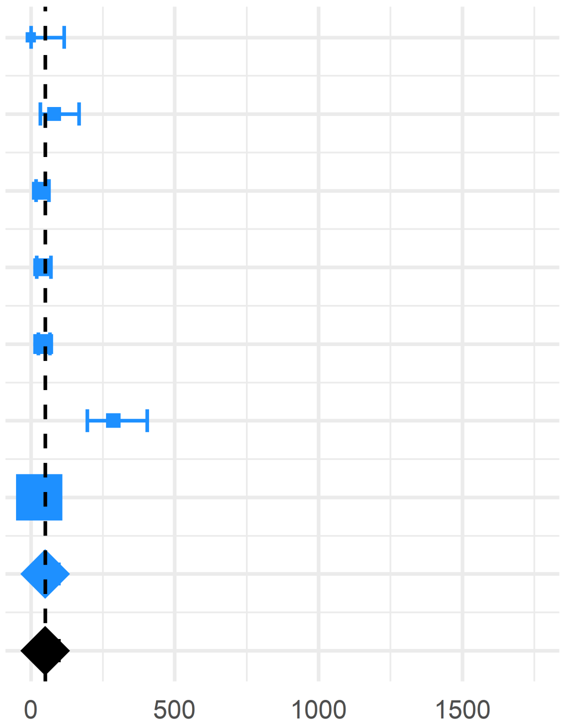

Heterogeneity:

1) $\tau^2 = 0.65$, $I^2 = 95.94$ %, $\chi^2 = 92.73$ (df = 6 , p-value = 0)

2) $\tau^2 = 0.65$, $I^2 = 95.94$ %, $\chi^2 = 92.73$ (df = 6 , p-value = 0)

Once-only colonoscopy categorized as: ND-longterm

Study	Events	People	Weight	Point estimate per 100.000 people, 95% CI
Khalid-de Bakker 2011	0	214	7.65 %	0 [0 , 1709]
Khalid-de Bakker 2011b	0	447	7.65 %	0 [0 , 822]
Lieberman 2000	0	3121	7.65 %	0 [0 , 118]
Berhane 2009	2	11808	15.66 %	17 [2 , 61]
Hamdani 2013	3	29396	17.72 %	10 [2 , 30]
Zwink 2017	8	5252	21.22 %	152 [66 , 300]
Rutter 2012	15	38472	22.46 %	39 [22 , 64]
1) Subtotal (not critical)	8	9034	44.17 %	21 [0 , 1812]
2) Subtotal (critical)	20	79676	55.84 %	21 [10 , 46]
3) Total	28	88710	100 %	25 [9 , 72]

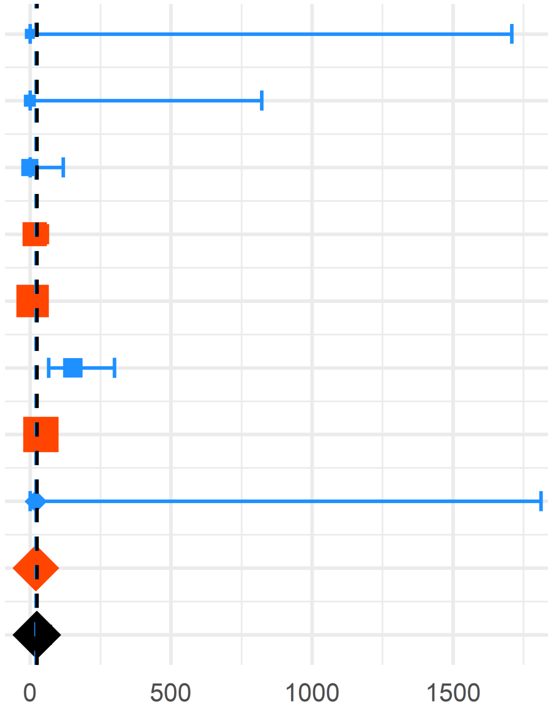

Heterogeneity:

1) $\tau^2 = 2.17$, $I^2 = 0$ %, $\chi^2 = 8.68$ (df = 3 , p-value = 0.0339)

2) $\tau^2 = 0.2$, $I^2 = 61.26$ %, $\chi^2 = 6.24$ (df = 2 , p-value = 0.0442)

3) $\tau^2 = 0.93$, $I^2 = 70.97$ %, $\chi^2 = 22.26$ (df = 6 , p-value = 0.001

Once-only colonoscopy categorized as: ND-NR

Study	Events	People	Weight	Point estimate per 100.000 people, 95% CI
Dae 2007	0	4491	2.89 %	0 [0 , 82]
Schoenfeld 2005	0	1483	2.89 %	0 [0 , 248]
Chiu 2013	0	18296	2.89 %	0 [0 , 20]
Strul 2006	0	1177	2.89 %	0 [0 , 313]
Zubarik 2002 (29a)	0	281	2.89 %	0 [0 , 1304]
Ahmed 2016	0	1049	2.89 %	0 [0 , 351]
Huppe 2004	1	1117	4.92 %	90 [2 , 498]
Imperiale 2000	1	1994	4.92 %	50 [1 , 279]
Quintero 2012 (27a)	1	5059	4.92 %	20 [1 , 110]
Bretthauer 2016	1	11912	4.92 %	8 [0 , 47]
Ferlitsch 2011	3	44350	9.27 %	7 [1 , 20]
Zwink 2017	4	5252	10.43 %	76 [21 , 195]
Xirasagar 2020	7	25008	12.41 %	28 [11 , 58]
Waldmann 2016	20	159246	14.87 %	13 [8 , 19]
Bielawska 2014	62	535286	16.02 %	12 [9 , 15]
1) Subtotal (not critical)	97	782103	70.81 %	12 [10 , 15]
2) Subtotal (critical)	3	33898	29.21 %	8 [2 , 43]
3) Total	100	816001	100 %	12 [10 , 15]

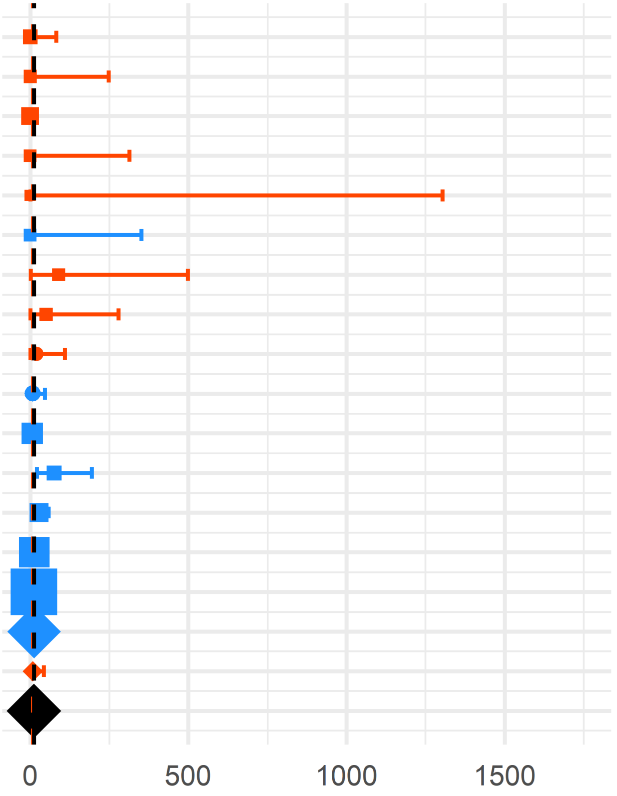

Heterogeneity:

1) $\tau^2 = 0$, $I^2 = 68.24$ %, $\chi^2 = 13.52$ (df = 6 , p-value = 0.0355)

2) $\tau^2 = 0.98$, $I^2 = 0$ %, $\chi^2 = 9.7$ (df = 7 , p-value = 0.2059)

3) $\tau^2 = 0$, $I^2 = 42.42$ %, $\chi^2 = 23.59$ (df = 14 , p-value = 0.0513)

Colonoscopy following FIT

Colonoscopy following FIT categorized as: Severe-NR

Study	Events	People		Weight	Point estimate per 100.000 people, 95% CI
Ellul 2010	0	1039		5.26 %	0 [0 , 354]
Marino 2012	1	1000		10.53 %	100 [3 , 556]
Denis 2007	2	2310		21.05 %	87 [10 , 312]
Binefa 2013	3	1691		31.58 %	177 [37 , 518]
Garcia 2012	3	989		31.58 %	303 [63 , 884]
1) Subtotal (critical)	9	7029		100 %	128 [67 , 246]
2) Total	9	7029		100 %	128 [67 , 246]

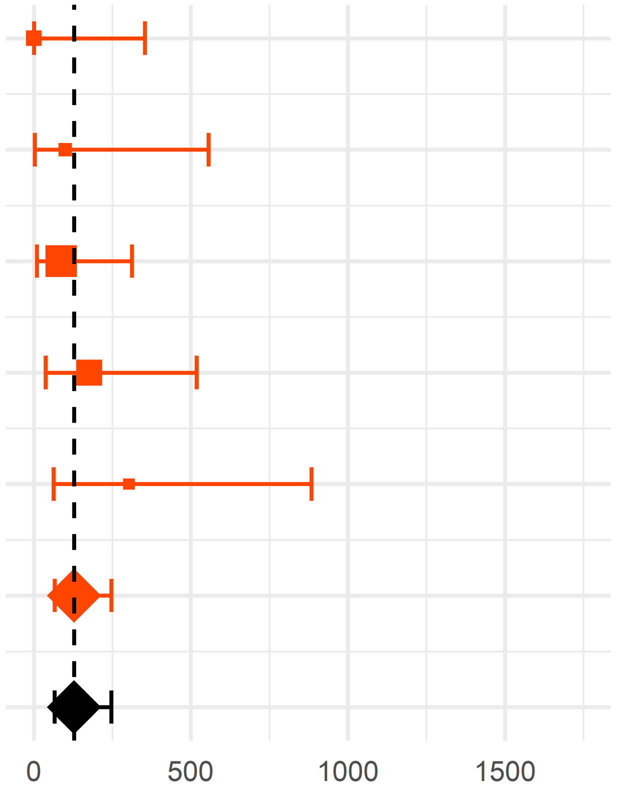

Heterogeneity:

1) $\tau^2 = 0$, $I^2 = 0$ %, $\chi^2 = 5.07$ (df = 4 , p-value = 0.28)

2) $\tau^2 = 0$, $I^2 = 0$ %, $\chi^2 = 5.07$ (df = 4 , p-value = 0.28)

Colonoscopy following FIT categorized as: Severe-longterm

Study	Events	People	Weight	Point estimate per 100.000 people, 95% CI
Saraste 2016	3	2984	5.56 %	101 [21 , 294]
Robertson 2019	5	17485	6.44 %	29 [9 , 67]
Denis 2013	10	10024	7.29 %	100 [48 , 183]
Ibanez 2018	13	47270	7.53 %	28 [15 , 47]
Din 2015 (2d)	16	38242	7.68 %	42 [24 , 68]
Denis 2021 (s4b)	18	9061	7.75 %	199 [118 , 314]
Lee 2012 (2b)	35	30829	8.06 %	114 [79 , 158]
Paszat 2020	57	121626	8.19 %	47 [35 , 61]
Benazzato 2020	65	99674	8.22 %	65 [50 , 83]
Vanaclocha-Espi 2018	98	41204	8.28 %	238 [193 , 290]
Arana-Arri 2018	106	36346	8.29 %	292 [239 , 353]
Portillo 2018	148	48759	8.33 %	304 [257 , 356]
Hsu 2020	277	214955	8.37 %	129 [114 , 145]
1) Subtotal (not critical)	745	682113	91.7 %	88 [56 , 138]
2) Subtotal (critical)	106	36346	8.29 %	292 [239 , 353]
3) Total	851	718459	100 %	97 [62 , 152]

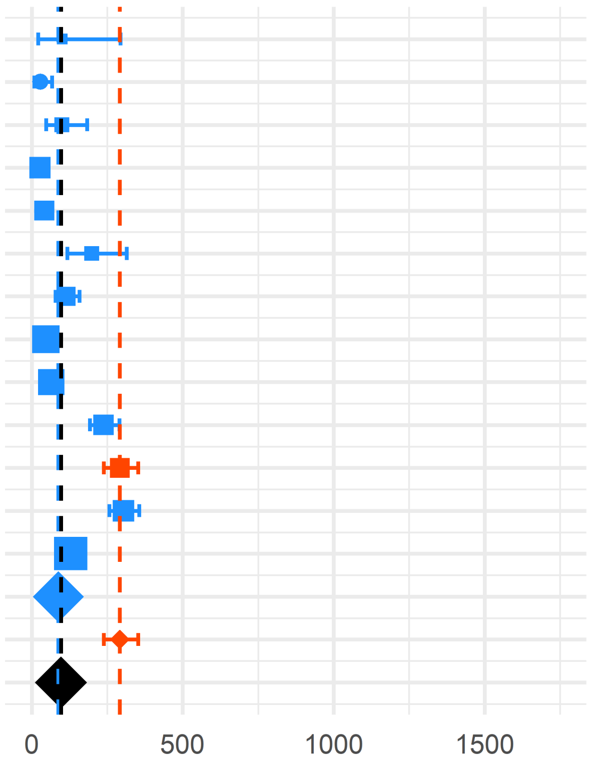

Heterogeneity:

1) $\tau^2 = 0.56$, $I^2 = 96.32$ %, $\chi^2 = 325.2$ (df = 11 , p-value = 0)

2) τ^2 = . , I^2 = . χ^2 = .

3) $\tau^2 = 0.62$, $I^2 = 96.6$ %, $\chi^2 = 395.36$ (df = 12 , p-value = 0)

Colonoscopy following FIT categorized as: Mild-longterm

Study	Events	People	Weight	Point estimate per 100.000 people, 95% CI
Randel 2021 (s23b)	3	6945	5.88 %	43 [9 , 126]
Tomaszewski 2021	48	78831	94.12 %	61 [45 , 81]
1) Subtotal (not critical)	51	85776	100 %	59 [45 , 78]
2) Total	51	85776	100 %	59 [45 , 78]

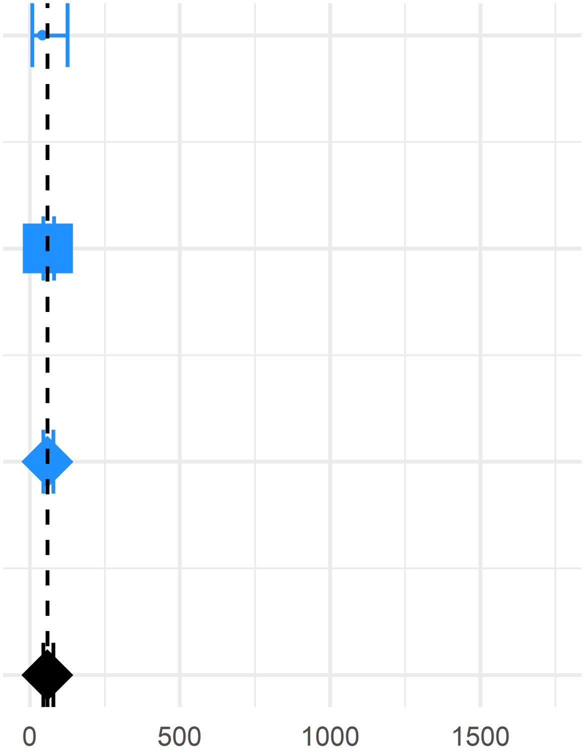

Heterogeneity:

1) $\tau^2 = 0$, $I^2 = 0$ %, $\chi^2 = 0.37$ (df = 1 , p-value = 0.5438)

2) $\tau^2 = 0$, $I^2 = 0$ %, $\chi^2 = 0.37$ (df = 1 , p-value = 0.5438)

Colonoscopy following FIT categorized as: ND-longterm

Study	Events	People	Weight	Point estimate per 100.000 people, 95% CI
Dominitz 2019	5	14661	3.66 %	34 [11 , 80]
Mikkelsen 2018	15	14671	9.44 %	102 [57 , 169]
Denis 2021 (s4a)	20	17152	11.76 %	117 [71 , 180]
Karlijn 2021	32	47270	16.25 %	68 [46 , 96]
Rutter 2014 (2a)	82	112024	26.54 %	73 [58 , 91]
Derbyshire 2018	147	222488	32.35 %	66 [56 , 78]
1) Subtotal (not critical)	301	428266	100 %	70 [63 , 79]
2) Total	301	428266	100 %	70 [63 , 79]

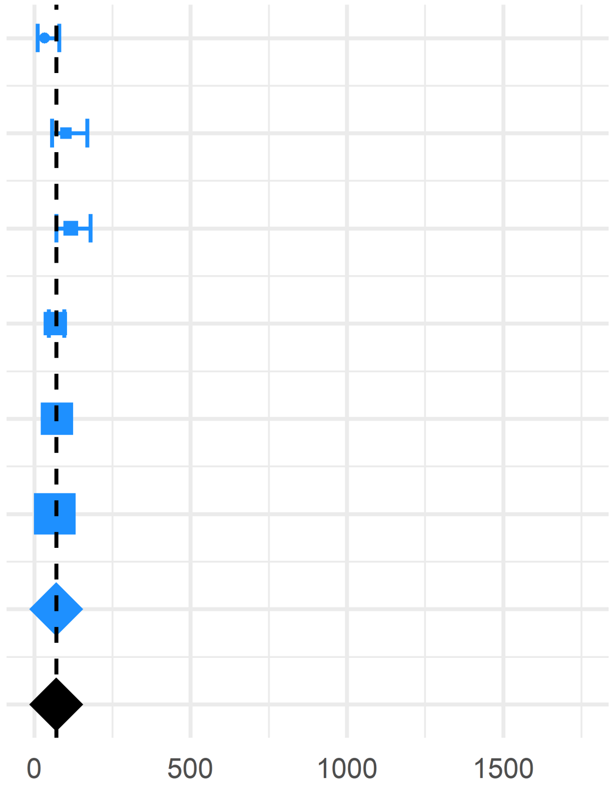

Heterogeneity:

1) $\tau^2 = 0$, $I^2 = 52.38$ %, $\chi^2 = 10.36$ (df = 5 , p-value = 0.0657)

2) $\tau^2 = 0$, $I^2 = 52.38$ %, $\chi^2 = 10.36$ (df = 5 , p-value = 0.0657)

Colonoscopy following FIT categorized as: ND-NR

Study	Events	People	Weight	Point estimate per 100.000 people, 95% CI
Denters 2013	0	273	1.88 %	0 [0 , 1342]
Sung 2003	0	476	1.88 %	0 [0 , 772]
Neely 2013	0	176	1.88 %	0 [0 , 2074]
Dancourt 2008	0	1205	1.88 %	0 [0 , 306]
Faivre 2004	0	1339	1.88 %	0 [0 , 275]
Quintero 2012 (27b)	0	663	1.88 %	0 [0 , 555]
Meulen 2021	0	225	1.88 %	0 [0 , 1626]
Gupta 2012	1	1057	3.39 %	95 [2 , 526]
Denters 2012	1	491	3.39 %	204 [5 , 1129]
Din 2017	2	780	5.68 %	256 [31 , 923]
Cheng 2002	2	6266	5.68 %	32 [4 , 115]
Parente 2013	2	3698	5.68 %	54 [7 , 195]
Steele 2004	2	4116	5.68 %	49 [6 , 175]
Florido 2017	2	842	5.68 %	238 [29 , 855]
Robinson 1999	5	1249	9.55 %	400 [130 , 932]
Tepes 2017	7	13674	10.97 %	51 [21 , 105]
Zorzi	27	29276	15.16 %	92 [61 , 134]
Quyn 2018	45	53332	16.01 %	84 [62 , 113]
1) Subtotal (not critical)	8	15207	16.24 %	53 [26 , 105]
2) Subtotal (critical)	88	103931	83.79 %	85 [69 , 104]
3) Total	96	119138	100 %	81 [66 , 98]

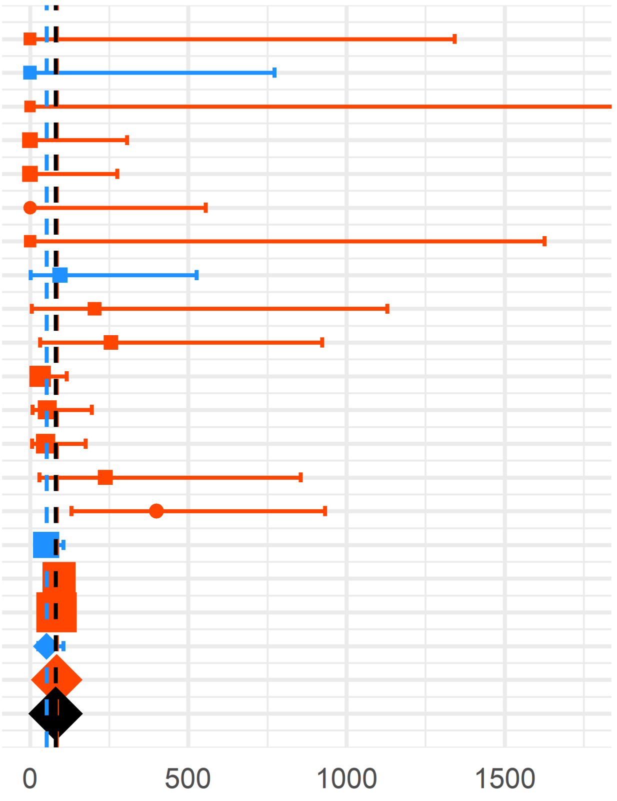

Heterogeneity:

1) $\tau^2 = 0$, $I^2 = 0$ %, $\chi^2 = 0.79$ (df = 2 , p-value = 0.673)

2) $\tau^2 = 0$, $I^2 = 26.84$ %, $\chi^2 = 22.23$ (df = 14 , p-value = 0.074)

3) $\tau^2 = 0$, $I^2 = 21.68$ %, $\chi^2 = 24.92$ (df = 17 , p-value = 0.0965)

Sigmoidoscopy

Sigmoidoscopy categorized as: Mild-longterm

Study	Events	People	Weight	Point estimate per 100.000 people, 95% CI
Segnan 2002 (50a)	1	9911	25 %	10 [0 , 56]
Randel 2021 (s23a)	3	36065	75 %	8 [2 , 24]
1) Subtotal (not critical)	3	36065	75 %	8 [2 , 24]
2) Subtotal (critical)	1	9911	25 %	10 [0 , 56]
3) Total	4	45976	100 %	9 [3 , 23]

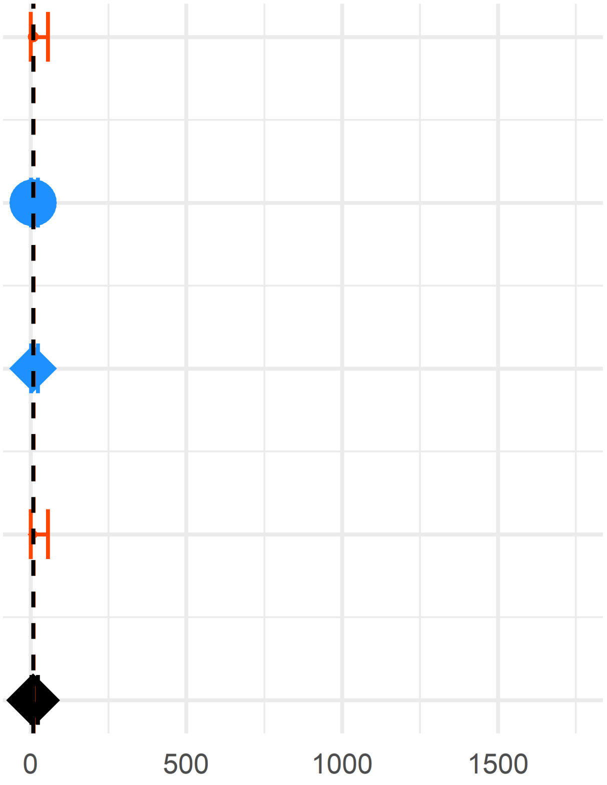

Heterogeneity:

1) τ^2 = . , I^2 = . , χ^2 = .

2) τ^2 = . , I^2 = . , χ^2 = .

3) τ^2 = 0 , I^2 = 0 %, χ^2 = 0.03 (df = 1 , p-value = 0.8694)

Sigmoidoscopy categorized as: ND-longterm

Study	Events	People	Weight	Point estimate per 100.000 people, 95% CI
Forbes 2006 (63b)	0	39	48.25 %	0 [0 , 9025]
Levin 2002	2	107704	51.75 %	2 [0 , 7]
1) Subtotal (not critical)	2	107743	100 %	2 [0 , 7]
2) Total	2	107743	100 %	2 [0 , 7]

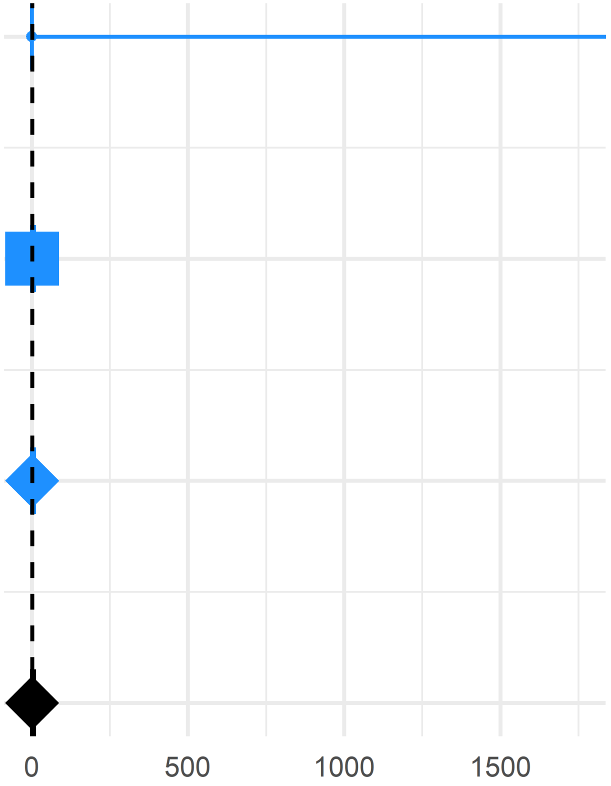

Heterogeneity:

1) $\tau^2 = 0$, $I^2 = 0$ %, $\chi^2 = 0$ (df = 1 , p-value = 0.9696)

2) $\tau^2 = 0$, $I^2 = 0$ %, $\chi^2 = 0$ (df = 1 , p-value = 0.9696)

Sigmoidoscopy categorized as: ND-NR

Study	Events	People	Weight	Point estimate per 100.000 people, 95% CI
Jain 2002	0	4229	13.79 %	0 [0 , 87]
Zubarik 2002 (29b)	0	185	13.79 %	0 [0 , 1974]
Holme 2014 (s34a)	0	12955	13.79 %	0 [0 , 28]
Aktin 2002 (36a)	1	40674	17.24 %	2 [0 , 14]
Schoen 2012 (72a)	3	67071	20.69 %	4 [1 , 13]
Kewenter 1996 (85a)	3	2108	20.69 %	142 [29 , 415]
1) Subtotal (not critical)	1	40674	17.24 %	2 [0 , 14]
2) Subtotal (critical)	6	86548	82.75 %	5 [0 , 84]
3) Total	7	127222	100 %	5 [1 , 36]

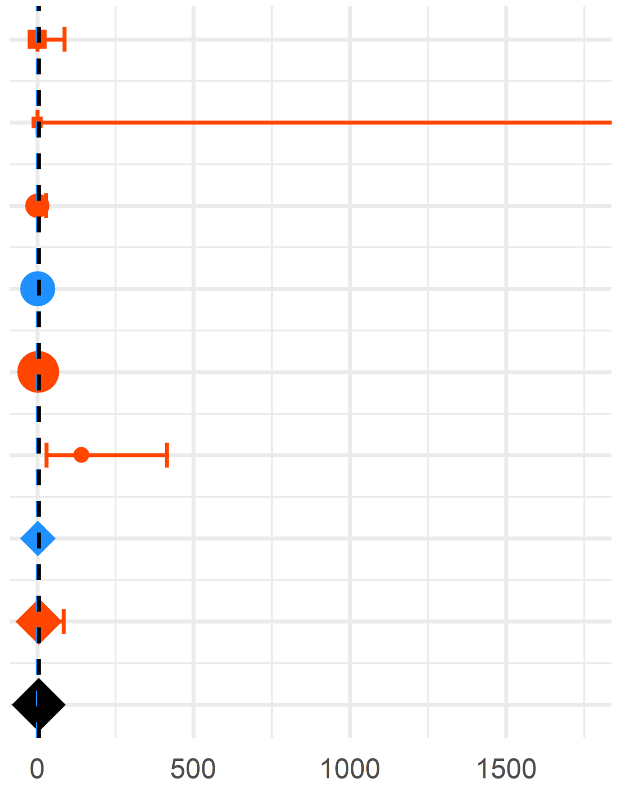

Heterogeneity:

1) τ^2 = . , I^2 = . , χ^2 = .

2) τ^2 = 3.81 , I^2 = 77.73 %, χ^2 = 15.5 (df = 4 , p-value = 0.0038

3) τ^2 = 3.14 , I^2 = 77.88 %, χ^2 = 16.66 (df = 5 , p-value = 0.005

Colonoscopy following any screening tests

Colonoscopy following any screening tests categorized as: Severe-NR

Study	Events	People	Weight	Point estimate per 100.000 people, 95% CI
Segnan 2002 (50b)	1	775	29.39 %	129 [3 , 717]
Mandel 1993	4	11943	70.61 %	33 [9 , 86]
1) Subtotal (critical)	5	12718	100 %	39 [16 , 94]
2) Total	5	12718	100 %	39 [16 , 94]

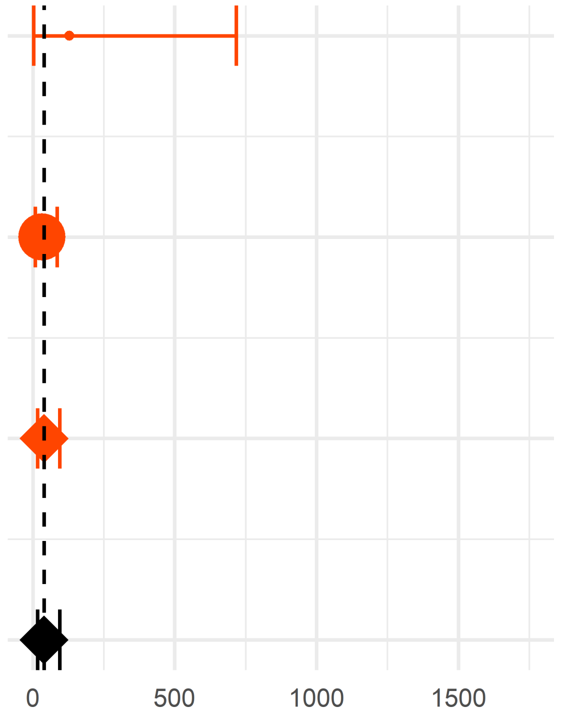

Heterogeneity:

1) $\tau^2 = 0$, $I^2 = 31.29$ %, $\chi^2 = 1.09$ (df = 1 , p-value = 0.2954)

2) $\tau^2 = 0$, $I^2 = 31.29$ %, $\chi^2 = 1.09$ (df = 1 , p-value = 0.2954)

Colonoscopy following any screening tests categorized as: Severe-longterm

Study	Events	People	Weight	Point estimate per 100.000 people, 95% CI
Shroff 2015	1	6152	39.09 %	16 [0 , 91]
Castro 2013	3	3215	60.91 %	93 [19 , 272]
1) Subtotal (critical)	4	9367	100 %	42 [13 , 138]
2) Total	4	9367	100 %	42 [13 , 138]

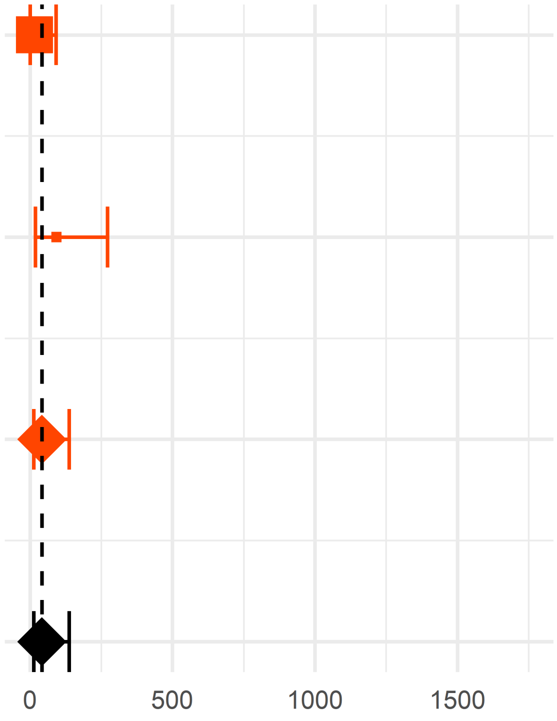

Heterogeneity:

1) $\tau^2 = 0.19$, $I^2 = 56.34$ %, $\chi^2 = 2.76$ (df = 1 , p-value = 0.0967

2) $\tau^2 = 0.19$, $I^2 = 56.34$ %, $\chi^2 = 2.76$ (df = 1 , p-value = 0.0967

Colonoscopy following any screening tests categorized as: ND-longterm

Study	Events	People	Weight	Point estimate per 100.000 people, 95% CI
Polter 2015	0	3059	7.14 %	0 [0 , 121]
Forbes 2006 (63a)	0	112	7.14 %	0 [0 , 3240]
Rutter 2012 (7b)	6	4984	85.71 %	120 [44 , 262]
1) Subtotal (not critical)	0	112	7.14 %	0 [0 , 3240]
2) Subtotal (critical)	6	8043	92.85 %	43 [3 , 552]
3) Total	6	8155	100 %	40 [3 , 595]

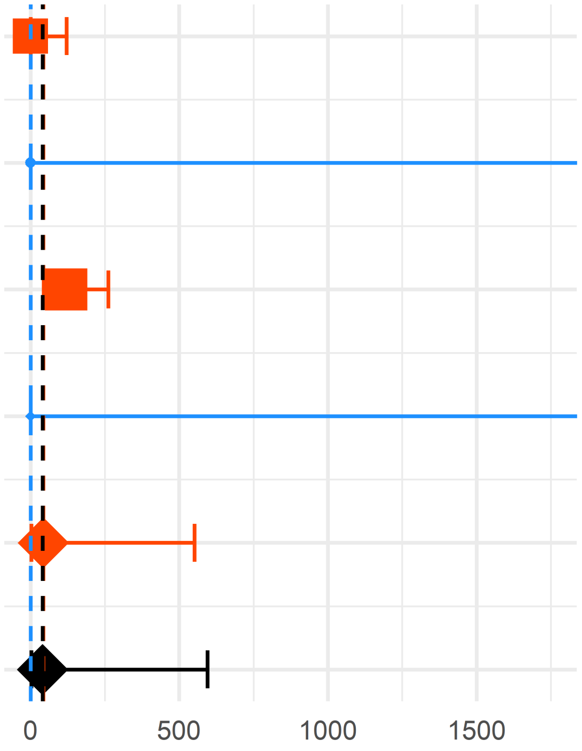

Heterogeneity:

1) τ^2 = . , I^2 = . , χ^2 = .

2) τ^2 = 1.05 , I^2 = 0 %, χ^2 = 5.74 (df = 1 , p-value = 0.0166)

3) τ^2 = 1.01 , I^2 = 0 %, χ^2 = 5.91 (df = 2 , p-value = 0.0521)

Colonoscopy following any screening tests categorized as: ND-NR

Study	Events	People	Weight	Point estimate per 100.000 people, 95% CI
Dellon 2009	0	3614	5.18 %	0 [0 , 102]
Kewenter 1996 (85b)	2	185	12.14 %	1081 [131 , 3850]
Naumann 2021	2	5219	12.14 %	38 [5 , 138]
Aktin 2002 (36b)	4	2051	15.65 %	195 [53 , 499]
Gondal 2003 (60b)	6	2524	17.31 %	238 [87 , 517]
Holme 2014 (s34b)	6	2746	17.31 %	218 [80 , 475]
Schoen 2012 (72b)	19	17234	20.27 %	110 [66 , 172]
1) Subtotal (not critical)	4	5665	20.83 %	32 [1 , 1270]
2) Subtotal (critical)	35	27908	79.17 %	147 [145 , 150]
3) Total	39	33573	100 %	121 [58 , 256]

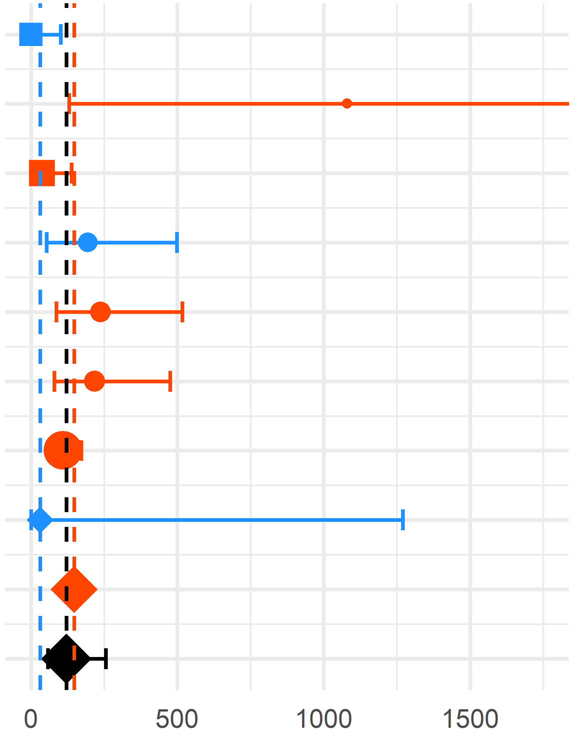

Heterogeneity:

1) $\tau^2 = 3.27$, $I^2 = 0$ %, $\chi^2 = 8.13$ (df = 1 , p-value = 0.0044)

2) $\tau^2 = 0.32$, $I^2 = 74.1$ %, $\chi^2 = 13.31$ (df = 4 , p-value = 0.0098

3) $\tau^2 = 0.69$, $I^2 = 61.71$ %, $\chi^2 = 22.82$ (df = 6 , p-value = 9e-04
