# Supplementary material for: The risk of bleeding and perforation from sigmoidoscopy or colonoscopy in colorectal cancer screening: A systematic review and meta-analyses
Source: PLoS One. 2023 Oct 31;18(10):e0292797. doi: 10.1371/journal.pone.0292797 (PMC10617695; doi:10.1371/journal.pone.0292797)
Supplement: Attachment — Submitted filename: 2023 04 03 Kindt and Martiny plus colleagues present an interesting systematic review on physical harms following endoscopy.docx [file pone.0292797.s009.docx]

Kindt and Martiny plus colleagues present an interesting systematic review on physical harms following endoscopy (plus or minus biopsies) of the colon in humans. In a very detailed and coherently reported systematic review they develop a new overview on how such harms can be separated in severity and proximity to the endoscopy as well as how often bleeding and perforation occurs. Moreover, they meta-analyse the literature and report that the occurrence of these complications seems to be more often occurring than described in previous attempts to systematically review comparable or similar topics. The messages are in general clearly presented and the topics described in detail. This systematic review provides important insights into the inadequate reporting and measurement of harms in studies of colorectal cancer screening and highlights the need for better reporting and measurement of harms in future studies. In the file I managed to download, I only have access to the first 8/25 appendixes.

I have the following comments and suggestions for amendments:

1. The Prospero protocol and the systematic review seem a bit like representing two universes. Have I missed an appendix entitled: changes from protocol to the review? I have full understanding that a protocol cannot be fully compatible with a systematic review after several hundred of studies and six or more years of work. I suggest that the authors develop an appendix that tries to describe the modifications that have been made on the journey so one understands the developments and potential weaknesses.
2. In the protocol, the authors write: “In summary, we have prioritized to include comparative studies to allow for comparison with existing reviews and to facilitate comparison of harm and harm assessment to benefit.” I fully accept that the authors do not assess benefits, but in their discussion they need to have a paragraph dealing with the balance between harms and benefits. The endoscopies are conducted to identify tumors and later to try to treat such tumors. I know that it has been hard to prove benefits of endoscopies, but think that the more recent trials and studies support some benefits.
3. I had to read the text several times and I did not become fully enlightened. What were the reasons for subdividing the endoscopy into ‘after FIT’ etc.? It should become fully clear why these subdivisions were conceived and if it is reasonable to keep them after the present finding. Moreover, how does the authors interpret similarities and dissimilarities in harm outcomes between the different subdivisions?
4. In a similar vein, what was the reason for using the subdivision into short term and long term? And why is short-term part of long term? Maybe the readability could be improved by deleting this rather not so informative latter division?
5. For the outcome death, I understand that the included studies do in fact not report on deaths that often. I would assume that death (all-cause mortality) would have been a natural part of such a review, why not choose death as an outcome? When I read the protocol, the authors are talking about severe adverse events, and here death is a natural component. And for all composite outcomes, one should analyse also the individual components. Moreover, having death as a separate outcome, the authors will have better abilities to highlight the lousy reporting of this important outcome.
6. In the discussion, you do not mention the risks of psychological harms as a limitation when one decides only to look at physical harms.
7. In the discussion, the authors write: “Any bias assessment may overlook, underestimate, or overestimate the risk of bias.” This is correct. However, our primary interest should be ‘Any assessed bias risk may overestimate or underestimate the reported findings’ ─ and especially if higher risk of bias is associated with underestimation of harms?
8. In an appendix, please compare observations from intervention groups of randomised trials to that of observational studies.
9. Please do not use any abbreviations in the abstract.
10. The title “The risk of physical harms from sigmoidoscopy or colonoscopy in colorectal cancer screening: a systematic review with meta-analyses on the risk of bleeding and perforation” should rather become “The risks of physical harms from sigmoidoscopy or colonoscopy in colorectal cancer screening: a systematic review of observational evidence”.
